# Experimental determination of the effects of pretreatment on selected Nigerian lignocellulosic biomass in bioethanol production

**DOI:** 10.1038/s41598-020-78105-8

**Published:** 2021-01-12

**Authors:** Adeolu A. Awoyale, David Lokhat

**Affiliations:** 1 Reactor Technology Research Group, School of Engineering, University of KwaZulu-Natal, Durban, South Africa; 2 Petroleum and Natural Gas Processing Department, Petroleum Training Institute, Effurun, Nigeria

**Keywords:** Energy science and technology, Engineering

## Abstract

In the present study, five lignocellulosic biomass namely, corn cobs (*Zea mays*), rice husks (*Oryza sativa*), cassava peels (*Manihot esculenta*), sugar cane bagasse (*Saccharum officinarum*), and white yam peels (*Dioscorea rotundata*) of two mesh sizes of 300 and 425 microns and a combination of some and all of the biomass were pretreated using combined hydrothermal and acid-based, combined hydrothermal and alkali-based and hydrothermal only processes. The raw and pretreated biomass were also characterized by Fourier transform infrared spectroscopy (FT-IR), Brunauer–Emmett–Teller (BET), X-Ray diffraction (XRD), and Scanning electron microscopy (SEM) to determine the effects of the various pretreatments on the biomass being studied. The cellulose values of the raw biomass range from 25.8 wt% for cassava peels biomass to 40.0 wt% for sugar cane bagasse. The values of the cellulose content increased slightly with the pretreatment, ranging from 33.2 to 43.8 wt%. The results of the analysis indicate that the hydrothermal and alkaline-based pretreatment shows more severity on the different biomass being studied as seen from the pore characteristics results of corn cobs + rice husks biomass, which also shows that the combination of feedstocks can effectively improve the properties of the biomass in the bioethanol production process. The FTIR analysis also showed that the crystalline cellulose present in all the biomass was converted to the amorphous form after the pretreatment processes. The pore characteristics for mixed corn cobs and rice husks biomass have the highest specific surface area and pore volume of 1837 m^2^/g and 0.5570 cc/g respectively.

## Introduction

The use of non-environmentally friendly fossil fuel as a source of energy has continued to pose serious challenges to the environment coupled with their non-renewability. Researchers worldwide have been seeking alternative sources of fuels for energy generation^1,2^. In the recent past, fuel bioethanol used to be produced from carbohydrate-rich foodstuffs like cassava, yam, wheat, sugar cane molasses, rice, barley, guinea corn etc. However, the production of bioethanol from food sources impacts negatively on the food chain for humans and animals. The processing of crops leaves a huge mass of wastes that were hitherto burnt off or disposed of indiscriminately, thereby causing environmental pollution^3^. The waste material left behind from processing the carbohydrate-rich crops is known as biomass. The biomass is rich in lignin, cellulose, and hemicellulose and is therefore more commonly referred to as lignocellulosic biomass^4^. Bioethanol produced from lignocellulosic biomass has been established to be a good alternative to fuels produced from fossil fuels. Examples of lignocellulosic biomass from which bioethanol can be produced include corn cobs, rice husks, cassava peels, yam peels, mango peels, sorghum straw, pineapple peels, potatoes peels, pawpaw peels, sugar cane bagasse among others^5^. Significant research is ongoing globally to develop industrial processes for bioethanol production from lignocellulosic biomass^6^. The major constituents in the make-up of lignocellulosic biomass are lignin, cellulose, and hemicellulose. As shown in the previous study^3^, the cellulose content of most of the lignocellulosic biomass found in Nigeria is very high, thereby making them potential raw materials for bioethanol production. Lignocellulosic biomass by nature is extremely resistant to being disintegrated to its component sugars owing to the lignin and hemicellulose parts of the biomass molecule hindering the access of the enzyme to the cellulose by joining themselves to the enzyme’s structure, thereby acting as an inhibitor. Cellulose, hemicellulose, and lignin are usually connected tightly in a macromolecular structure, thereby making the pore size of the molecules to be reduced, this reduction in the pore size enhances the inhibiting characteristics of the lignin and hemicellulose. Pretreatment or ‘prehydrolysis’ is the process through which the cellulose constituent is subjected to and made susceptible to enzymatic hydrolysis. Lignocellulosic biomass requires suitable and adequate pretreatment for the resultant hydrolysis to take place since they are recalcitrant. The efficacy of the pretreatment process influences both the up-stream choice of feedstock, cellulose, hemicellulose, and lignin components recovery efficiently, the chemical and morphological features of the ensuing cellulosic component, which consequently controls downstream hydrolysis and ultimately fermentation to bioethanol^7^.

Lignocellulosic biomass feedstocks are diverse; therefore, it is not easy to specify a general pretreatment process for all of them. Several pretreatment technologies have been proposed in recent times. The pretreatment method selected for pretreating the biomass has a significant effect on bioethanol production cost and yield^8^. Pretreatment technologies can be categorized into physical, chemical, biological, and physio-chemical or a combination depending on the diverse forces or resources expended in the pretreatment process^9^. The liquid hot water (LHW) pretreatment is a form of hydrothermal pretreatment which requires no rapid decompression and no catalyst or chemical addition. Temperature and pressure range of between 170 and 230 °C and > 5 Mpa respectively are used in LHW pretreatment^10^. The LHW pretreatment eliminates hemicellulose from lignocellulosic materials and exposing the cellulose thereby making it available for fermentation. After the pretreatment, the resulting slurry can then be sieved to acquire a solid rich in cellulose and another liquid portion with hemicellulose sugars^11^. Acid pretreatment is a form of chemical pretreatment that involves chemical hydrolyses which solubilizes hemicellulose and lignin, thereby making the cellulose more open for enzymes action during the fermentation process. The acid pretreatment can be carried out with concentrated or dilute acid, but the use of concentrated acid comes with a drawback of the formation of impeding compounds such as furfural and phenolic acids, besides, concentrated acids are noxious, corrosive, and usually hazardous. The pretreatment should, therefore, be carried out in corrosion-resistant equipment. Dilute acid pretreatment is mainly suitable for large scale bioethanol production. Various reactors like the plug flow, shrinking bed, batch, flow-through, among others have been developed for this process^12^.

An alkaline pretreatment is also a form of chemical pretreatment whereby a base like potassium, sodium, ammonium, and calcium hydroxides at standard pressure and temperature is used to treat the biomass. This pretreatment method has the advantage of being more efficient in removing lignin from the biomass. The method also eliminates acetyl and uronic acid groups existing on hemicellulose and therefore boosts the accessibility of enzyme that breaks down hemicellulose^13^. Alkali pretreatment can also be applied at low temperatures, pressure, and time. Sodium hydroxide is more effective than others^14^. A few researchers have also attempted to combine two pretreatment processes for substantially increasing the yield of reducing sugars such as the combination of alkaline pretreatment (lime) with the oxidative delignification process^15^. To assess the efficacies of the various pretreatment techniques in the preparation of the lignocellulosic biomass for the transformation of the enzymes, the results of a standard cellulase treatment on the pretreated biomass can be compared^16^. The severity factor is frequently employed to explain lignin reduction and xylan solubility^17,18^. Pereira et al.^19^ worked on the ‘physical–chemical–morphological characterization of the whole sugarcane lignocellulosic biomass used for second-generation (2G) ethanol production by spectroscopy and microscopy techniques’. Their work entailed detailed analysis of the bagasse, straw, and tops of sugarcane through NMR, FTIR, XRD, and SEM. The result from their work shows that skeletal aromatic and methoxyl groups attributable to lignin structure are present in sugarcane lignocellulosic biomass. In the work of Zhang et al.^20^, ‘XRD was used to study the interactions of cellulose in lignocellulosic biomass with ionic liquids’. The result obtained from the study shows that with an increment in pretreatment temperature using an ionic liquid, there is a drop-in crystallinity index of the biomass, a phenomenon ascribed to the swelling of the crystalline cellulose.

Modern pre-treatment methods can take above 40% of the entire production costs and is the most energy-consuming part of converting lignocellulosic biomass to biofuels. Furthermore, the pre-treatment processes involve the use of substantial amounts of corrosive reagents as well as heating methods which can gravely impact negatively on the environment. It is therefore of utmost importance to reduce the pre-treatment costs and the negative impact they might have on the environment. A novel appraisal means currently being utilized for conversion studies is the life cycle assessment (LCA), which helps in the identification and evaluation of the environmental performance and sustainability of different pre-treatment techniques. The LCA measures and decode information over a product and process life cycle, in terms of their production, use, and end-of-life. Numerous researches have been done in the past using the LCA procedure to analyze the environmental impact of bioethanol production from various lignocellulosic feedstocks^21^.

The main highlight of this work is to bring home the pre-treatment technologies available in the research space to Nigeria which is just coming up in renewable energy development with slight modifications to the existing processes. To this end, multiple biomass readily sourced from Nigeria were used for this research and a combination of the biomass feedstock also gave valuable information that can be utilized in the lignocellulosic bioethanol development of the country. Moreover, there are not many reports about one of the biomass in this study, which is yam peels, in literature. 96% of the world’s yam production is from West Africa and two-thirds of these are from Nigeria. The data supplied by this manuscript on yam peels biomass as a feedstock for bioethanol production will go a long way in providing a reference for future research in this area. The same goes for the combinations of feedstocks which is more representative of a commercial application where a single processing plant would obtain a variety of different agricultural residues as reported in this study. There are no significant studies reported on these hybrid feedstocks and the characteristics of the pre-treated materials. This study also elaborately compares the effects of the three pre-treatment processes adopted on several biomass.

## Experimental

Materials and Biomass preparation. The rice husks in this study were sourced from a rice mill in Ekperi Etsako Central Local government Area of Edo State Nigeria, the corn cobs were obtained from Ogume, Ndokwa West Local Government Area of Delta State Nigeria, the cassava peels, yam peels, and sugar cane bagasse were all sourced from Effurun, Uvwie Local Government Area of Delta State Nigeria. All the sourced biomass were then sundried for about 7 days and then taken to the grinding mill for grounding after which they were sieved into two particle sizes of 300 and 425 microns respectively. Analytical grade chemical reagents such as sodium hydroxide pellets, hydrogen peroxide, and tetraoxosulphate (VI) acid were used. 1500 g each of the yam peels biomass, cassava peels biomass, rice husks biomass, corn cobs biomass, and 1000 g of sugarcane bagasse biomass were measured and kept inside different vessels. To study the effects of biomass combinations, 300 g each of the 300 microns for all the five biomass was measured and mixed in a vessel. Also, 750 g each of 300 microns particle size cassava and yam peels biomass and corn cobs and rice husks biomass was measured and transferred into different vessels.

### Pretreatment methods

In this study, three pretreatment methods were adopted to pretreat the biomass for comparison purposes. The pretreatment methods used include combined hydrothermal and acid-based pretreatment, combined hydrothermal and alkaline-based pretreatment, and hydrothermal only pretreatment.

#### Combined hydrothermal and acid pretreatment

The method used by Utama et al.^22^ was adopted in this work with slight modification. H_2_SO_4_ (98% analytical grade JHD) with 98.08 g/mol molecular weight was used. 80 ml of the sulphuric acid was measured and transferred into a 2000 ml volumetric flask and distilled water was used to make up the volume to 2000 ml. 0.75 M solution of the sulphuric acid was thus obtained. The prepared biomass (individual and combinations) were then soaked with the prepared acid solution in batches and for each case, about 4 to 6 L of distilled water was used alongside the 2 L of the 0.75 M solution of the H_2_SO_4_ based on the absorbing capacity of the biomass. The mixture was then thoroughly mixed in the vessel before being transferred to a pressure pot and allowed to boil for about an hour. It was allowed to cool to ambient temperature before filtering and then kept inside sampling plastics. The filtrate from each batch was also stored separately.

#### Combined hydrothermal and alkaline pretreatment

160 g of NaOH pellets were dissolved in a beaker and then moved to a 2000 ml volumetric flask containing 60 ml of H_2_O_2_ and then thoroughly mixed in the volumetric flask before making it up to the 2000 ml mark with distilled water. The same procedure as that of acid pretreatment was then followed on all the biomass.

#### Hydrothermal pretreatment

Hydrothermal only Pretreatment was also done on all the biomass as described in the combined hydrothermal and acid pretreatment as control.

### Characterization of the raw and pretreated biomass

Characterization of biomass is essential to establish its capability for bioethanol production^23^. The raw and pretreatment biomass samples were then subjected to characterization to establish the impacts of the different pretreatment methods on the biomass meant for fermentation for bioethanol production. The following physicochemical analysis was carried out on the raw and pretreated biomass: proximate analysis, ultimate analysis, FT-IR, XRD, BET, and SEM. The characterization was done at the multiuser laboratory of Ahmadu Bello University, Zaria Nigeria, and the Chemical Engineering Department laboratory of Federal University of Technology Minna, Nigeria.

#### Proximate analysis

The gross composition of the biomass pre and post pretreatment was determined using the proximate analysis to ascertain their moisture content, ash content, lignin content, cellulose content, and hemicellulose content.

##### Determination of lignin content

The acid detergent fiber (ADF) residue earlier obtained was immersed in chill sulphuric acid. The mixture was blended to a smooth paste to breakdown all the arms. The residue in the crucible was dehydrated for 24 h at 100 °C and then allowed to cool to around ambient temperature. It was then weighed and labeled (W1). The crucible plus oven-dried residue was moved to a muffle furnace fixed at 550 °C to ash for 3 h till a white greyish residue was obtained, cooled in a desiccator, and then weighed and labeled (W2). The lignin content was then calculated using the equation;

1 $$ Lignin\;content= \frac{W1-W2}{weight \;of \;sample}\times 100\% $$

##### Determination of holocellulose

A solution of 80 ml acetic acid and 1 g of sodium chloride were put into 2.5 g of extractive free sample (after extracting the sample) in a water bath hourly for six (6) h in a process known as chlorinating. Subsequently, after six (6) h of chlorinating, the samples were then allowed to stay for a while in a water bath to lower the temperature, and then the holocellulose was filtered using a Buchner funnel. The initial and final weight of the holocellulose were taken and the holocellulose content was calculated using the following formula:

2 $$Holocellulos \;content= \frac{W1-W2}{weight \;of \;sample}\times 100\%$$

where: W1—the weight of the sample before the process, W2—the weight of the sample after the process.

##### Determination of hemicellulose

2 g of holocelloluse which had earlier been dried in the oven was transferred into a 25 ml glass beaker, afterwards, 10 ml of 17.5% sodium hydroxide (NaOH) solution was mixed with the holocelloluse, it was then made to stay in a water bath, a flat end glass rod was employed in stirring it for it to be soaked with the NaOH, after adding of the initial portion of 17.5% (NaOH) solution every five (5) min, additional 5 ml of NaOH solution was then introduced and thoroughly agitated using a glass rod. Sodium hydroxide was continuously added until all the NaOH solution was used up. Thereafter, the mixture was made to stay in a water bath for thirty (30) min.

##### Determination of cellulose

The cellulose content of the biomass was determined using the formula:

$${\text{Holocellulose }} = {\text{ Cellulose }} + {\text{ Hemicellulose}}$$

$${\text{Cellulose }} = {\text{ Holocellulose }}{-}{\text{ Hemicellulose}}$$

##### Determination of moisture content

The moisture content of the biomass was gotten by the method of oven drying. This was done at a temperature of 103 ± 2 °C following ASTM D1037 (1991). The moisture content was thereafter determined by using the equation:

3 $$M= \frac{Wf-Wi}{W1}\times 100\%$$

where Wi—the initial mass of the sample, Wf—the final mass of the sample.

##### Determination of the ash matter

The Ash matter in the biomass samples was determined by the method described in ASTM D2017 (1998). 1 g each of the samples was put in a pre-weighed crucible and was then burnt in a muffle furnace at 760 °C until ashing was completed after which the container was then moved into a desiccator to lower the temperature. Three replicates were made. The samples were then weighed after cooling. The ash matter was determined by using the following equation:

4 $$Ash \;content (\%)= \frac{W2-W0}{W1-W0}\times 100\%$$

where: W0 = Weight of the container, W1 = weight of the container + biomass sample before burning, W2 = weight of the container + biomass sample after burning.

#### FT-IR, SEM, BET and XRD characterization of the raw and pretreated samples

The virgin and pretreated biomass were also characterized by Fourier transform infrared spectroscopy (FT-IR), Brunauer–Emmett–Teller (BET), X-Ray diffraction (XRD), and Scanning electron microscopy (SEM).

##### Fourier transform infrared (FT-IR) spectroscopy

The main function of FT-IR spectroscopy is for the detection of the different functional groups present in the virgin and pretreated biomass^24^. The main result of FTIR assays mainly deals with the lignin content of the biomass^25^. For this work, Agilent Cary 630 FTIR Spectrometer was used to characterize the virgin and the pretreated biomass. The FTIR was analyzed using Agilent MicroLab PC software equipped with the equipment.

##### Scanning electron microscopy (SEM)

This is an analytical procedure that scans a sample with an electron beam to produce a magnified image for assessment. SEM analysis makes the samples structures to be assessed and their elemental make-up determined^26^. The Phenom ProX desktop SEM with a magnification range of 20–100,000× and element detection range of C–Am and acceleration voltage of 10 kV was used in this study.

##### Brunauer–Emmett–Teller (BET) analysis

The specific surface area of a biomass sample and the pore size distribution can be measured with this analysis, which can be used to forecast the dissolution rate as this rate is proportional to the specific surface area and the surface area, in turn, can be used to forecast bioavailability. The Nova 4200e BET analyzer was used in this study. The BET analyzer used nitrogen as an analysis gas. The outgas time was 3 h at a temperature of 250 °C. The pressure tolerance for the analysis was 0.100/0.100 (ads/des). The equilibrium time was 60/60 s and the equilibrium time out was 240/240 s. The analysis time was 111.8 min.

##### X-ray diffraction (XRD) analysis

This is a swift analytical procedure used to detect phases of lignocellulosic biomass and can also provide information on the cell composition of the biomass. The lignocellulosic biomass meant for analysis should be in fine particulate form and thoroughly mixed before the mean bulk components are determined. This test is centered on the applied intrusion of monochromatic X-rays and a lignocellulosic biomass sample. The X-ray is generated by the Cathode ray tube, which has a monochromatic radiation production. The X-rays are also collimated to meet and directed at the lignocellulosic biomass sample. The incident rays interact with the lignocellulosic biomass sample to enable the creation of positive interference and a diffracted ray after conditions meet Bragg’s law (nλ = 2 d sin θ). The diffracted X-rays are then detected, sorted out and calculations carried out. The lignocellulosic biomass samples, with a range of 2θ angles for all likely diffraction paths of the lattice to be achieved, as the grounded lignocellulosic biomass is unsystematically orientated. As a result of every mineral having a set of exclusive d-spacings, diffraction peaks were converted to d-spacing to spot the minerals existing in the biomass. A comparison of the d-spacings with a standard reference pattern was then carried out.

### Life cycle assessment (LCA)

The LCA is equipped with the data collection section, which is meant for the identification and accounting of all the input and output of a process. In building and analyzing LCA models, thorough, holistic, and generally admitted stock data is needed for the materials and processes used. SimaPro has been a leading LCA software package that is used to extract considerable amounts of related data from diverse production handling reports, which includes chemical and food production facilities and brings in the data from a varied collection of accessible databanks. Ecoinvent v3 LCI Databank was also employed in this study. This is the most frequently consulted and referenced^27^.

### Economic consideration of the bioethanol production process

Bioethanol production process economic assessment usually includes the estimation of yields, financial considerations, and costs related to the investment and operating costs. To determine the efficiency of the pre-treatment process, the bioethanol yield from the hybrid biomass (cassava peels and yam peels biomass) for the acid-based and alkali-based pre-treatments were evaluated using the model used by Solarte-Toro et al.^28^. The assessment also included obtaining the utility costs, which comprise the cooling water, steam, fuel, and electricity required for pre-treatment, fermentation, and distillation of the fermented hydrolysates, cost of raw materials and transportation, labour costs, operating charges, Fermentation and distillation equipment Costs, administrative costs, cost of enzymes and depreciation. The data were evaluated using engineering economics and descriptive statistics. The bioethanol cost was then compared with the current market price of bioethanol as obtainable in Nigeria.

## Results and discussion

### Effects of different pretreatment rigorousness on the physicochemical properties of biomass

The results of the proximate analysis on the raw and pretreated biomass samples of different particle sizes are as shown in Tables 1, 2, 3, 4. The cellulose values of the raw biomass range from 25.8 wt% for cassava peels biomass to 40.0 wt% for sugar cane bagasse. This result conforms with what is obtainable in literature as reported by Salihu et al.^29^. The pretreated biomass shows a significant difference from the raw biomass with values ranging from 33.2 to 43.8 wt%. The values of the cellulose content increased slightly with the pretreatment and that of the lignin content decreasing significantly with pretreatment. High cellulose and low lignin contents is a very desirable quality in lignocellulosic biomass for bioethanol production^30^. The hydrothermal and alkaline-based pretreatment shows more severity on the different biomass being studied with the lignin content reducing significantly with values ranging from 12.4 wt% for cassava peels to 22.8 wt% for a mixture of all the biomass. This also conforms to the findings of Sabiha-Hanim et al.^31^ and Chang et al.^13^ that both found out from their works that alkaline pretreatment is more efficient in lignin removal and considerably increases the degradability of cellulose even if only some part of the lignin is removed.

**Table 1:** Proximate analysis result for unpretreated (raw) biomass samples.

Biomass	Moisture content (%)		Ash content (%)		Lignin content (%)		Hemi cellulose (%)		Cellulose (%)	
	300 μm	425 μm	300 μm	425 μm	300 μm	425 μm	300 μm	425 μm	300 μm	425 μm
Cassava peels	18.3	15.0	8.0	2.0	20.2	21.6	36.0	35.3	25.8	33.2
Corn cobs	21.0	20.0	3.0	4.0	19.2	19.2	34.0	36.0	36.0	34.0
Rice husks	21.0	18.0	10.0	2.0	24.4	25.2	34.5	31.5	33.5	31.0
Sugar cane bagasse	22.0	17.0	6.0	6.0	9.2	12.4	45.0	45.0	39.8	40.0
Yam peels	26.0	27.0	6.0	10.0	21.6	20.0	39.0	28.0	36.8	38.1

**Table 2:** Proximate analysis result for hydrothermal and acid-based pretreated samples.

Biomass	Moisture content (%)		Ash content (%)		Lignin content (%)		Hemi cellulose (%)		Cellulose (%)	
	300 μm	425 μm	300 μm	425 μm	300 μm	425 μm	300 μm	425 μm	300 μm	425 μm
Cassava peels	19.0	18.0	4.0	10.0	20.8	24.4	35.0	34.5	33.0	36.5
Corn cobs	19.0	25.0	4.0	2.7	20.4	20.8	34.8	35.0	34.7	34.5
Rice husks	28.0	27.0	10.0	9.0	20.0	18.8	33.5	36.0	35.0	34.5
Sugar cane bagasse	19.0		10.0		17.6		43.5		42.0	
Yam peels	20.0		2.0		22.4		36.0		34.5	
Cassava peels + yam peels	25.0		3.0		24.0		37.5		36.5	
Corn cobs + rice husks	22.0		2.0		24.4		36.0		35.5	
The mixture of all the five biomass	22.0		10.0		24.0		37.9		36.1

**Table 3:** Proximate analysis result for hydrothermal and alkali-based pretreated samples.

Biomass	Moisture content (%)	Ash content (%)	Lignin content (%)	Hemi cellulose (%)	Cellulose (%)
	300 μm	300 μm	300 μm	300 μm	300 μm
Cassava peels	19.0	2.0	12.4	43.8	42.2
Corn cobs	27.0	6.0	20.8	34.0	35.0
Rice husks	27.0	10.0	19.6	35.3	33.2
Sugar cane bagasse	29.0	8.0	16.0	41.8	43.7
Cassava peels + yam peels	25.0	8.0	22.0	35.0	36.0
Corn cobs + rice husks	22.0	4.0	21.2	34.3	34.2
The mixture of all the five biomass	24.0	10.0	22.8	35.3	36.7

**Table 4:** Proximate analysis result for hydrothermal only pretreated samples.

Biomass	Moisture content (%)		Ash content (%)		Lignin content (%)		Hemicellulose (%)		Cellulose (%)	
	300 μm	425 μm	300 μm	425 μm	300 μm	425 μm	300 μm	425 μm	300 μm	425 μm
Cassava peels	17.0	20.0	1.8	4.2	17.6	20.0	41.5	38.3	43.5	35.2
Corn cobs	26.0	20.0	10.0	10.0	17.6	15.6	43.5	43.2	41.5	43.8
Rice husks	21.0	20.0	10.0	7.0	21.6	21.6	33.5	35.0	34.5	33.5
Sugar cane bagasse	25.0	–	10.0	–	17.6	–	43.5	–	41.4	–
Yam peels	17.0	–	10.0	–	24.0	–	35.9	–	35.6	–
Cassava peels + yam peels	23.0	–	3.0	–	24.4	–	35.5	–	36.5	–
Corn cobs + rice husks	22.0	–	7.0	–	22.8	–	35.8	–	35.7	–
The mixture of all the five biomass	23.0	–	3.0	–	22.0	–	35.0	–	35.5	–

The ash content of the pretreated biomass except for rice husks biomass which remained the same were all reduced. It is worthy of note that elevated ash content is a problem as the ash particles in the biomass take up more steam, H_2_O, dilute acid solution, or diluent than the relatively larger lignocellulosic fibers^32^. The consequence of the ash content of the lignocellulosic biomass could also be seen on the moisture content results for the pretreated biomass. Since the ash particles in the biomass absorb the steam, water, dilute acid, and other solvents during pretreatment, the moisture content of the biomass increased slightly after pretreatment with the alkali pretreated biomass having the highest moisture content, again confirming the superior severity of the alkali pretreatment over the other pretreatment methods adopted in this study. The hemicellulose content also shows the same pattern by increasing slightly after the different pretreatments, with the values of the pretreated biomass ranging from 33.5 to 43.5 wt%. For the mixed biomass (cassava peels plus yam peels, corn cobs plus rice husks, and all five biomass combined), there is not much difference in the outcome of the different pretreatment methods. The values obtained for their cellulose and hemicellulose content for the three pretreatments falls within a very narrow range. Total delignification of the biomass is not easy due to the position of lignin in the macromolecular structure^33^.

The proximate analysis parameters for the three pretreatment methods adopted in this study were analyzed statistically using analysis of variance (ANOVA) of Microsoft Excel and the results are as shown in Table 5a–d. The p-values in the ANOVA results for the ash content, lignin content, hemicellulose content, and cellulose content were all greater than 0.05, showing that there is no significant difference in the effects of the different pretreatments on the biomass in this study. The statistical significance of the data obtained for the proximate analysis parameters was tested by F-test and shows that the effects of the three pretreatment methods was highly significant as suggested by the model F values on the tables.

**Table 5:** Statistical analysis of the pretreatment parameters.

a. Ash content						
Groups	Count	Sum	Average	Variance		
Acid pretreatment	8	45	5.625	13.69643		
Alkaline pretreatment	7	48	6.857143	9.142857		
Hot water	8	54.8	6.85	13.55143		

ANOVA						
Source of variation	SS	df	MS	F	P-value	F crit
Between groups	7.872205	2	3.936102	0.32054	0.729417	3.492828
Within groups	245.59214	20	12.27961			
Total	253.46435	22				

b. Lignin content						
Groups	Count	Sum	Average	Variance		
Acid pretreatment	8	173.6	21.7	5.794286		
Alkaline pretreatment	7	134.8	19.25714	13.99619		
Hot water	8	167.6	20.95	8.545714		

ANOVA						
Source of variation	SS	df	MS	F	P-value	F crit
Between groups	23.07242	2	11.53621	1.251507	0.307534	3.492828
Within groups	184.3571	20	9.217857			
Total	207.4296	22				

c. Hemicellulose content						
Groups	Count	Sum	Average	Variance		
Acid pretreatment	8	294.2	36.775	9.427857		
Alkaline pretreatment	7	259.5	37.07143	15.88571		
Hot water	8	304.2	38.025	16.785		

ANOVA						
Source of variation	SS	df	MS	F	P-value	F crit
Between groups	6.775714	2	3.387857	0.243028	0.786532	3.492828
Within groups	278.8043	20	13.94021			
Total	285.58	22				

d. Cellulose content						
Groups	Count	Sum	Average	Variance		
Acid pretreatment	8	287.3	35.912	7.198		
Alkaline pretreatment	7	261	37.285	16.454		
Hot water	8	304.2	38.025	12.265		

ANOVA						
Source of variation	SS	df	MS	F	P-value	F crit
Between groups	18.339	2	9.169	0.780	0.471	3.492
Within groups	234.972	20	11.748			
Total	253.312	22				

### Effect of pretreatment on the composition of biomass

The major purpose of pretreatment is the breaking down of the lignin make-up and the disruption of the crystalline make-up of cellulose for improving enzyme ease of access to the cellulose in the hydrolysis step^34^. Tables 6, 7, 8, 9, 10, 11, 12, 13 show the pore characteristics of the different biomass being analyzed in this study. The BET adsorption isotherms are also shown in Figures S1 to 8. The results show that generally for all the biomass under consideration, there is a significant reduction in specific surface area and pore volume with the different pretreatment methods carried out on them, with hydrothermal only pretreatment showing the least values. The results also show that cassava peels biomass of 300 microns particle size has the highest specific surface area and pore volume of 819.6 m^2^/g and 0.2031 cc/g respectively for individual pretreated biomass, thereby upholding the assertion by many researchers that cassava peel biomass is a very good feedstock for bioethanol production^35,36^. For the combined biomass, the combined corn cobs and rice husks biomass with specific surface area and pore volume of 1837 m^2^/g and 0.5570 cc/g respectively show promising potential for improved yield of bioethanol after the fermentation process.

**Table 6:** Pore characteristics of cassava peels biomass (raw and pretreated).

Sample	Specific surface area (m^2^/g)			Pore volume (cc/g)		Pore diameter (nm)	V_micro_/V_Total_ (%)
	S_BET_	S_micro_	S_Ext_	V_Total_	V_micro_		
Raw (300 μm)	541.8	819.6	574.5	0.2552	0.2031	2.647	0.7958
Raw (425 μm)	293.0	525.7	530.4	0.1364	0.0743	2.647	0.5447
Acid pretreated (300 μm)	252.4	470.5	475.1	0.1201	0.0655	2.647	0.5453
Acid pretreated (425 μm)	301.1	502.1	399.3	0.1429	0.0890	2.647	0.6228
Alkali pretreated (300 μm)	278.8	448.9	360.5	0.1319	0.0815	2.647	0.6178
Hot water pretreated (300 μm)	276.1	440.2	355.4	0.1316	0.0812	2.647	0.6170
Hot water pretreated (425 μm)	242.9	415.6	349.4	0.1150	0.0676	2.647	0.5878

**Table 7:** Pore characteristics of corn cobs biomass (raw and pretreated).

Sample	Specific surface area (m^2^/g)			Pore volume (cc/g)		Pore diameter (nm)	V_micro_/V_Total_ (%)
	S_BET_	S_micro_	S_Ext_	V_Total_	V_micro_		
Raw (300 μm)	336.2	571.0	471.3	0.1599	0.0934	2.647	0.5841
Raw (425 μm)	304.3	498.8	434.9	0.1437	0.0817	2.647	0.5685
Acid pretreated (300 μm)	234.2	402.3	331.9	0.1104	0.0658	2.647	0.5960
Acid pretreated (425 μm)	239.1	405.5	360.7	0.1123	0.0646	2.647	0.5752
Alkali pretreated (300 μm)	310.3	500.7	393.6	0.1473	0.0927	2.647	0.6293
Hot water pretreated (300 μm)	264.5	436.6	345.2	0.1255	0.0778	2.647	0.6199
Hot water pretreated (425 μm)	237.9	410.3	379.5	0.1112	0.0629	2.647	0.5656

**Table 8:** Pore characteristics of rice husks biomass (raw and pretreated).

Sample	Specific surface area (m^2^/g)			Pore volume (cc/g)		Pore diameter (nm)	V_micro_/V_Total_ (%)
	S_BET_	S_micro_	S_Ext_	V_Total_	V_micro_		
Raw (300 μm)	288.0	503.7	464.9	0.1343	0.0757	2.647	0.5636
Raw (425 μm)	227.2	871.4	418.3	0.1056	0.0575	2.647	0.5445
Acid pretreated (300 μm)	275.7	463.0	376.5	0.1303	0.0788	2.647	0.6047
Acid pretreated (425 μm)	279.1	453.3	363.2	0.1326	0.0822	2.647	0.6199
Alkali pretreated (300 μm)	253.0	446.9	416.0	0.1198	0.0664	2.647	0.5542
Hot water pretreated (300 μm)	246.5	432.3	413.5	0.1151	0.0639	2.647	0.5551
Hot water pretreated (425 μm)	262.8	433.4	371.9	0.1234	0.0725	2.647	0.5875

**Table 9:** Pore characteristics of sugar cane bagasse biomass (raw and pretreated).

Sample	Specific surface area (m^2^/g)			Pore volume (cc/g)		Pore diameter (nm)	V_micro_/V_Total_ (%)
	S_BET_	S_micro_	S_Ext_	V_Total_	V_micro_		
Raw (300 μm)	278.6	491.3	456.1	0.1322	0.0728	2.647	0.5506
Raw (425 μm)	241.9	422.8	392.5	0.1143	0.0629	2.647	0.5503
Acid pretreated (300 μm)	310.0	531.9	486.4	0.1469	0.0813	2.647	0.5534
Alkali pretreated (300 μm)	252.5	407.1	341.0	0.1184	0.0699	2.647	0.5903
Hot water pretreated (300 μm)	229.6	411.4	385.3	0.1087	0.0600	2.647	0.5519

**Table 10:** Pore characteristics of yam peels biomass (raw and pretreated).

Sample	Specific surface area (m^2^/g)			Pore volume (cc/g)		Pore diameter (nm)	V_micro_/V_Total_ (%)
	S_BET_	S_micro_	S_Ext_	V_Total_	V_micro_		
Raw (300 μm)	269.1	488.1	475.1	0.1281	0.0701	2.647	0.5472
Raw (425 μm)	244.4	409.1	356.3	0.1147	0.0660	2.647	0.5754
Acid pretreated (300 μm)	239.1	406.2	348.6	0.1125	0.0650	2.647	0.5777
Alkali pretreated (300 μm)	242.9	408.4	354.2	0.1156	0.0671	2.647	0.5804
Hot water pretreated (300 μm)	232.4	400.3	340.7	0.1098	0.064	2.647	0.5828

**Table 11:** Pore characteristics of cassava plus yam peels biomass.

Sample	Specific surface area (m^2^/g)			Pore volume (cc/g)		Pore diameter (nm)	V_micro_/V_Total_ (%)
	S_BET_	S_micro_	S_Ext_	V_Total_	V_micro_		
Acid pretreated (300 μm)	499.9	864.1	818.2	0.2337	0.1325	2.647	0.5669
Alkali pretreated (300 μm)	738.3	1118.0	760.5	0.3478	0.3003	1.847	0.8634
Hot water pretreated (300 μm)	518.6	888.4	719.3	0.2455	0.1447	2.647	0.5894

**Table 12:** Pore characteristics of corn cobs plus rice husks biomass.

Sample	Specific surface area (m^2^/g)			Pore volume (cc/g)		Pore diameter (nm)	V_micro_/V_Total_ (%)
	S_BET_	S_micro_	S_Ext_	V_Total_	V_micro_		
Acid pretreated (300 μm)	1181.0	1837.0	1257.0	0.5570	0.4481	2.647	0.8044
Alkali pretreated (300 μm)	440.9	811.3	833.2	0.2057	0.1124	2.647	0.5464
Hot water pretreated (300 μm)	402.2	748.4	800.5	0.2022	0.1004	2.647	0.4965

**Table 13:** Pore characteristics of the combination of all the biomass.

Sample	Specific surface area (m^2^/g)			Pore volume (cc/g)		Pore diameter (nm)	V_micro_/V_Total_ (%)
	S_BET_	S_micro_	S_Ext_	V_Total_	V_micro_		
Acid pretreated (300 μm)	471.7	870.1	879.4	0.2223	0.1188	2.647	0.5344
Alkali pretreated (300 μm)	489.4	893.3	889.9	0.2283	0.1252	2.647	0.5484
Hot water pretreated (300 μm)	516.8	895.0	791.5	0.2449	0.1411	2.647	0.5761

### FTIR spectroscopy of raw and pretreated biomass

**Figure 1:**
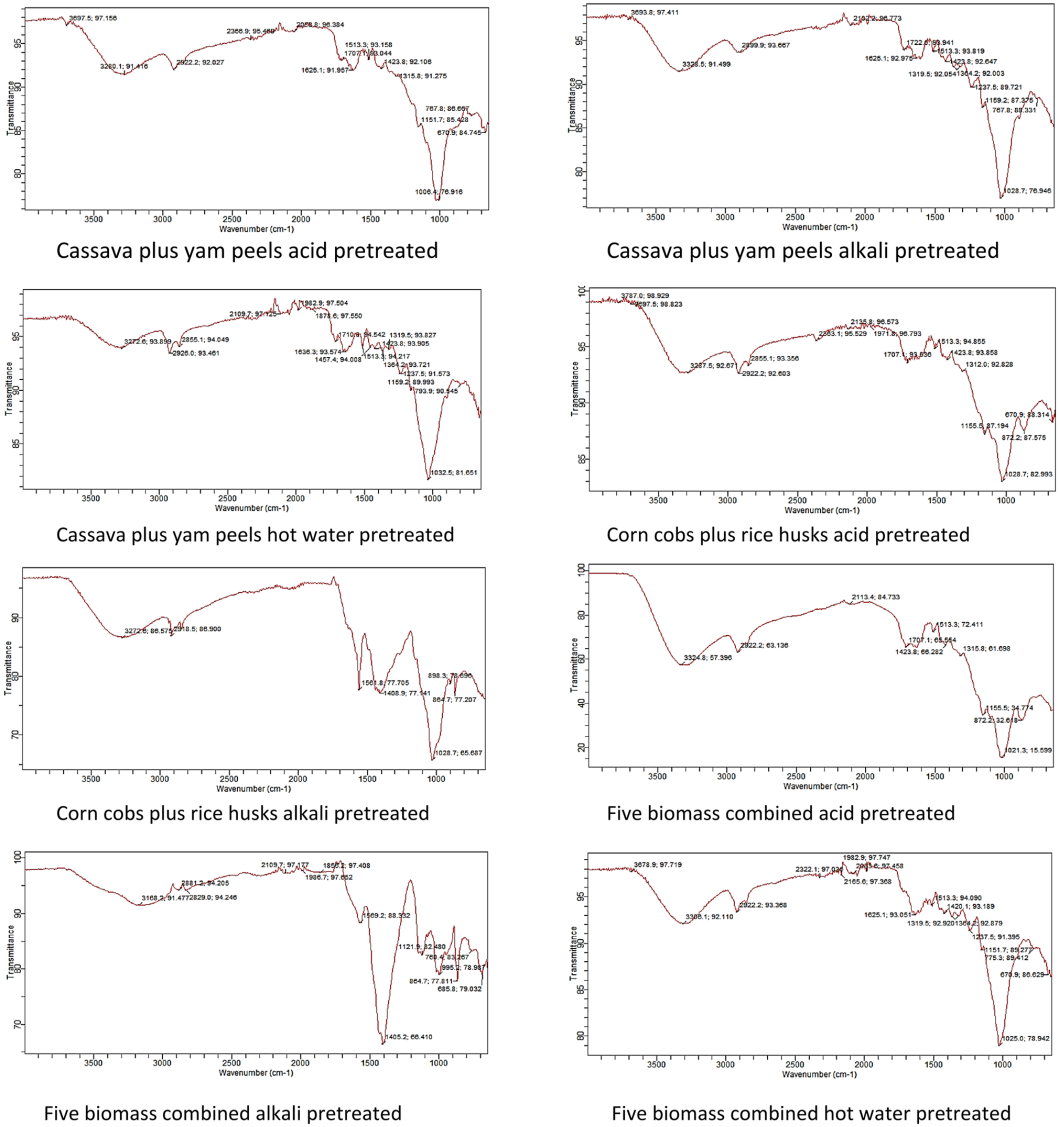
FTIR of combined biomass (300 microns).

FTIR is mainly used to characterize the biomass based on the organic groups present^37^. The focal point of FTIR analysis is the lignin content, which is an aromatic biopolymer composed chiefly of phenylpropane substituted components attached to form a giant molecule of non-consistent crystallinity and optical activity^38^. The FTIR spectra of the untreated (raw) as well as the treated biomass are shown in Figs. 1 and S9 to S13. Table 14 displays the functional groups and vibration modes of the raw and pretreated biomass at standard temperature. The absorption band of the raw and pretreated biomass between 3200 and 3600 cm^−1^ is usually attributed to the O–H stretching vibrations of alcohols, carboxylic acids, and hydroperoxides^39,40^. The FTIR spectrum shows a fingerprint region of 1420–670 for the source identification of the biomass. The results show that the O–H stretching of the hydroxy group of alcohol falls between 3693 and 3008 cm^−1^ for acid pretreated cassava peels and acid pretreated rice husks respectively. The methyl group of alkanes has a band of 2926 to 2855 cm^−1^ for water pretreated corn cobs and alkali pretreated corn cobs respectively. C=O stretching vibration can be attributed to ketones with a wavenumber of between 1636 and 1606 cm^−1^. The band 1457 cm^−1^ shows the C–H bending or scissoring of alkanes found in acid pretreated corn cobs, cassava peels, and a combination of yam and cassava peels. Other organic compounds detected include ether, ether, and Β-glucosidic bonds (864 cm^−1^) between sugars^41^. For all the biomass, a very sharp peak of between 1006 to 1028 cm^−1^ of C–O–C is noted. The strong and sharp peak is attributed to the ether group. The hemicellulose content of the biomass can be ascribed to the wavebands at 1710 and 1028 cm^−1^. There are also very sharp peaks noticed on the pretreated combined corn cobs and rice husks biomass, this is an indication of the fortification of the properties of the biomass by their combination.

**Table 14:** Organic functional groups detected in the raw and pretreated biomass.

Wavenumber (cm^−1^)	Organic functional groups	Description of vibration
3693–3008	Hydroxyl	This is due to the O–H stretching of the alcohol group present
2926–2855	Alkanes	This is as a result of the stretching of methyl (C–H) group
2855	Methoxy	The assigned peak is due to OCH_3_
1710–1718	Carboxylic acids	This band is due to the stretching vibration of C=O
1606–1636	Ketones	C=O stretching vibration attributed to ketones
1560–1610	Furan	C=C ring stretching vibrations
1420–670	Fingerprint region	
1457	Alkanes	C–H bending or scissoring of alkanes
1364–1367	Phenolic	O–H phenolic group assigned peak
1159	Esters	O–C=O stretching of the esters group
1006–1028	Ethers	C–O–C The strong and sharp peak is credited to the ethers group
864	Β-glucosidic bonds between sugars	

### Inhibitor analysis of the different pretreatment process

The results of the proximate analysis as shown in Table 2 indicate that the combined hydrothermal and acid pretreated biomass has a very high recovery of hemicellulosic sugars with increased enzymatic convertibility. However, the process brings about the formation of inhibitory derivatives such as aliphatic carboxylic acids, furans, among others. The combined hydrothermal and alkali pretreatment was able to remove lignin and a small fraction of hemicelluloses, however with the formation of some side products such as acetic acid, hydroxy acids among others. Table 15 shows a summary of the inhibitory side products formed during the different pretreatment methods in this study.

**Table 15:** Summary of inhibitory side products formed during the different pretreatment methods.

Pretreatment method	Main effects	Inhibitory products detected (from FTIR analysis)
Hydrothermal and acid-based pretreatment	Hydrolysis of hemicelluloses to monosaccharides	Carboxylic acids, phenylic compounds
Hydrothermal and alkali based pretreatment	Removal of lignin and a minor part of hemicelluloses	Acetic acid, hydroxy acids, phenolic compounds
Hydrothermal only based pretreatment	Solubilization of hemicelluloses	Acetic acid, furan, aldehydes

In the work by Olsson et al.^42^, to reduce the effects of the inhibitors on the hydrolyzates for fermentation, two methods were proposed namely; detoxification and adaptation of the microorganism to the lignocellulosic hydrolysate. The latter is more cost-saving than the former. Other methods include overliming^43^, charcoal adsorption, and ion exchange^44^.

### Structural changes in the raw and pretreated biomass

**Figure 2:**
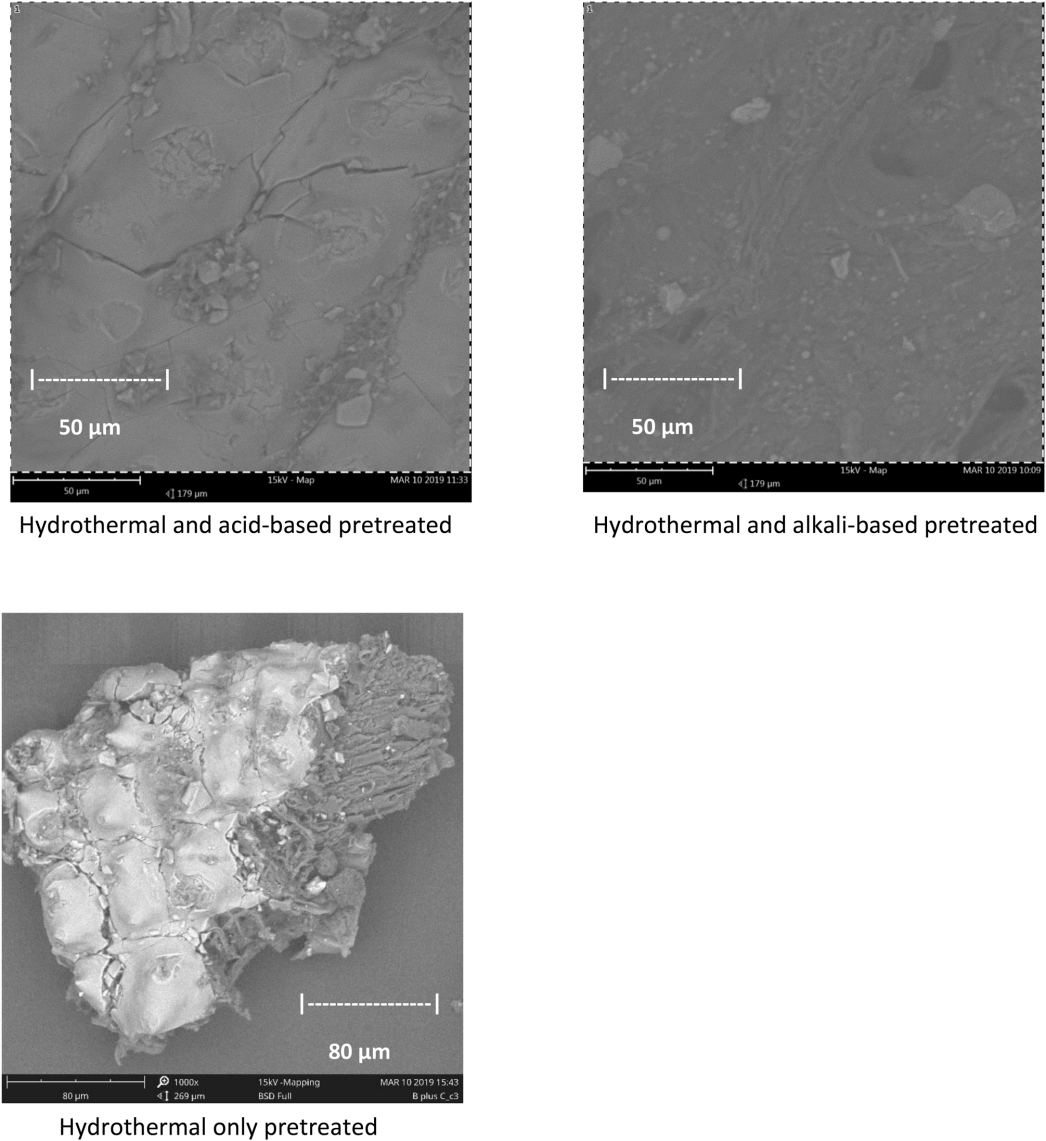
SEM of pretreated corn cobs plus rice husks biomass (300 microns).

The scanning electron micrograph (SEM) was employed to compare, study and analyze the untreated samples as well as morphological changes that had occurred on the pretreated samples as a result of the different pretreatments carried out on them. Figures 2 and S14 to S20 show the images of the morphological analysis of the raw and pretreated biomass by SEM. The images show that all the raw biomass had a smooth intact structure with a rigid and fibrillary morphology, which had not been damaged by the crushing and grinding of the biomass. The images also revealed interesting transformations after the pretreatment processes were carried out on them. The morphologies of the pretreated biomass show that they were broken down and fragmented with the hitherto compacted and finely divided surfaces unsettled. The SEM images of the pretreated biomass also showed a cluster of globe-like micro grains deposited over copious particles, this is an indication that cellulose and hemicellulose were disintegrated to a reasonable extent during the pretreatment processes^45^. However, it can be observed that the images of the alkali pretreated biomass show more of the globe like clusters, indicating the severity of alkali pretreatment over the other pretreatment processes carried out in this study.

This result conforms with the pattern of results obtained by Chowdhury^46^ in their work on lignocellulosic biomass. Lignin, which is a non-saccharide fraction of biomass is more chemically rigid than the saccharide fraction, composed of cellulose and hemicellulose. The lignin was moderately disintegrated, and the initial properties of the grains were conserved. The images also show that pore volume with the surface area was considerably enlarged after the pretreatment process with the alkali pretreatment showing the highest severity. This observation was also supported by the results obtained from the BET analysis.

### X-ray diffraction (XRD)

**Figure 3:**
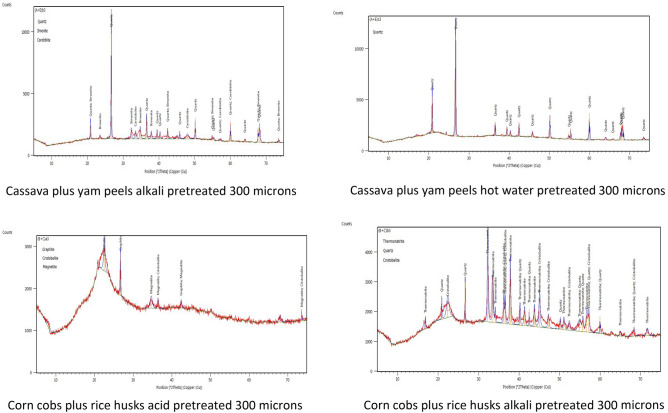
XRD diffractograms of mixed biomass.

The X-ray diffraction patterns of the raw and pretreated biomass are illustrated in Fig. 3 and Figures S21 to S26. As can be seen, all the diffractograms showed the typical XRD peaks of cellulose. For the raw biomass, a sharp peak at 2θ values of between 18 and 22° was observed owing to the presence of crystalline cellulose in the biomass samples. This conforms to the results obtained from previous research^47^. The pretreated biomass samples showed diffractograms with peaks of somewhat reduced intensities, a demonstration of incomplete degradation of the cellulose with pretreatment^48^. The pretreated biomass samples had a broader peak at 2θ values of between 22 and 24°, which is an indication of atomic order in them. The non-existence of sharp peaks proves the amorphous texture of the pretreated biomass samples^49^. As opposed to amorphous cellulose, crystalline cellulose is more recalcitrant to enzymatic and microbial reactions^50,51^.

### Environmental considerations

Many factors influence the determination of the life cycle effects from bioethanol production from different biomass; this includes cultivation of the feedstock, production of the enzymes and chemical, pre-treatment, fermentation, delivery, and utilization of the bioethanol. Emissions to air, soil, and water during the pre-treatment process were assessed using SimaPro. The environmental impact categories include global warming potential (GWP)/climate change, eutrophication (EP), acidification (AP), photochemical oxidation demand (POD), and marine and human ecotoxicity. The emissions from the pre-treatment processes can be classified in terms of corresponding quantities using the CML (Centre of Environmental Science at Leiden University) method^52^. Potential for global warming is measured in kg of CO_2_ (eq), eutrophication is measured in kg of PO_4_^–3^ (eq), acidification is measured in kg of SO_2_ (eq), photochemical oxidation demand is measured in kg of C_2_H_4−_ (eq), while marine and human ecotoxicity are measured in kg of dichlorobenzene (1,4 C_6_H_4_Cl_2_) (eq). The GWP and the AP were considered on the mixed biomass of cassava peels and yam peels in this work.

#### Global warming potential (GWP)

The global warming potential (GWP) of a process or product and its consequent effect on the climate, have been the current subject of debate and regulation in the environmental performance assessment. GWP is an enumerated amount of the global mean comparative radiative driving effects of a certain greenhouse gas measured over a 100-year timeline. The CML technique provides GHG emissions in units of corresponding liberated CO_2_ via the global warming potential factors of 1, 25, and 298 for CO_2_, CH_4,_ and NO_2_, respectively. With an average value of 15.82 kg CO_2_ (eq), the alkaline pre-treatment using sodium hydroxide shows the highest discharge of GHG emissions, while acid pretreatment using dilute sulphuric acid generated an average 8.68 kg CO_2_. Climate change is a very important consideration in lignocellulosic bioethanol production as one of the reasons for utilizing bioethanol for energy purposes is the reduction in GHG emissions.

#### Acidification potential (AP)

Acidification stems from the anthropogenic emissions involving sulfur (iv) oxide (SO_2_), nitrogen oxides (NO_x_), and ammonia (NH_3_). The assessment of the acidifying potential of the sulphuric acid and sodium hydroxide used for pre-treatment of one of the biomass in this study (cassava peels biomass), show that sulphuric acid emitted 0.035 kg SO_2_ (eq), a value lower than that of sodium hydroxide emission, which was 0.087 kg SO_2_ (eq). This result is an indication that the acid pre-treatment is more preferable in terms of environmental degradation owing to the effect of the biomass pre-treatment.

### Economic consideration results and analysis

To be viable and economically acceptable, the expenditure for the processing of lignocellulosic biomass to fuel must not be up to the current gasoline cost. This is achievable owing to the efforts of researchers at improving the effectiveness of the biomass processing technologies. The cost of feedstock, feedstock pre-treatment, and enzymes are important considerations for low-cost ethanol production. The use of hybrid (mixed) feedstocks and large-scale processing facilities coupled with low-cost feedstock as well as effective cellulases helps in making the process cost-effective. In converting biomass to bioethanol, some inputs result in environmental costs, the output from the process such as electricity generations and sales of the produced bioethanol would result in the recouping of the expenses of the production process. Table 16 shows the results of the techno-economic assessment of bioethanol produced from acid-based pre-treated cassava and yam peels biomass mixture. The bioethanol price of 0.41 USD/l is a good deal as it compares favorably well with the 0.45 USD/l price of ethanol in the Nigerian market. The produced bioethanol could also augment gasoline from crude oil, this would also reduce the drastic effects of the combustion of gasoline on the environment in terms of emissions and costs. The major advantage derivable from the use of biomass in bioethanol production is the limiting of greenhouse gases' environmental pollution^53^. The lignocellulosic bioethanol production, therefore, comes with more benefits in the long run in terms of economic and environmental considerations.

**Table 16:** Techno-economic assessment results (Cassava and yam peels biomass mixture).

Item	Acid-based pre-treated Cassava and yam peels biomass		References
	USD/year	(%)	
Raw materials^a^	1672	15.20	
Utilities^b^	1721	15.65	
Labour cost	1707	15.52	
Maintenance	550	5.00	
Operating charges	522	4.75	
Plant overhead^c^	578	5.25	
Administrative costs	338	3.07	
Depreciation	1533	13.94	
Total production cost		100	
Total project capital cost (USD)^d^	25,923.86		
Bioethanol cost (USD/l)	0.41		
Current market bioethanol cost (USD/l)	0.45		^54^
Current market gasoline cost (USD/l)	0.42^e^		^55^

The assessment is for a plant designed to process approximately 10 tons/year of cassava and yam peels biomass.

^a^This includes costs of sourcing and transportation of the agricultural residues.

^b^This includes costs of cooling water, steam, fuel, and electricity required for pre-treatment, fermentation, and distillation of the fermented hydrolysates.

^c^This includes costs of enzymes and reagents for pre-treatment.

^d^This includes costs of reactors, fermenters, distillation equipment, plant building, and furniture.

^e^Subject to further increment as the government is reviewing the fuel subsidy policy.

## Conclusion

Bioethanol production from lignocellulosic biomass such as corn cobs, rice husks, cassava peels, yam peels, sugar cane bagasse among others, is an emerging technology in the renewable energy field. However, these biomass must be pretreated before the fermentation process. The main goal aimed to be achieved by carrying out pretreatment is the breaking down of the lignin structure and the interruption of the crystalline make-up of cellulose for enhancing enzyme accessibility to the cellulose during the hydrolysis stage. Selected biomass was sourced locally, dried, and sieved into two mesh sizes and then pretreated using hydrothermal and acid-based based, hydrothermal, and alkali-based and hydrothermal only processes. The raw as well as the pretreated biomass samples were then characterized by proximate analysis, SEM, FTIR, XRD, and BET. The cellulose values of the raw biomass range from 25.8 wt% for cassava peels biomass to 40.0 wt% for sugar cane bagasse. The pretreated biomass shows a significant difference from the raw biomass with values ranging from 33.2 to 43.8 wt%. The cassava peels biomass of 300 microns particle size has the highest specific surface area and pore volume of 819.6 m^2^/g and 0.2031 cc/g respectively for individual pretreated biomass, while for the combined biomass, the combined corn cobs and rice husks biomass with specific surface area and pore volume of 1837 m^2^/g and 0.5570 cc/g respectively. The combined corn cobs and rice husks biomass show a promising potential for improved bioethanol yield after fermentation. The organic compounds present in the biomass include hydroxyl, alkanes, methoxy, carboxylic acids, ketones among others. The use of a combined biomass feedstock would be preferred as this will give greater flexibility in the bioethanol production from lignocellulosic biomass, and as was shown from this work, the combination of different biomass materials can result in favorable properties of the combined biomass feedstock after pretreatment.

## Supplementary information

Supplementary Information.
