# Supplementary material for: Experimental determination of the effects of pretreatment on selected Nigerian lignocellulosic biomass in bioethanol production
Source: Sci Rep. 2021 Jan 12;11:557. doi: 10.1038/s41598-020-78105-8 (PMC7804122; doi:10.1038/s41598-020-78105-8)
Supplement: Supplementary file 1 — Supplementary Information. [file 41598_2020_78105_MOESM1_ESM.docx]

**Experimental determination of the effects of pretreatment on selected Nigerian lignocellulosic biomass in Bioethanol production**

Adeolu A. Awoyale ^a,b,^*, and, David Lokhat ^a^

^a^ Reactor Technology Research Group, School of Engineering, University of KwaZulu-Natal, Durban, South Africa. ^b^ Petroleum and Natural Gas Processing Department, Petroleum Training Institute, Effurun, Nigeria

* Corresponding author: Reactor Technology Research Group, School of Engineering, University of KwaZulu-Natal, Durban, South Africa

E-mail address: [oluyale@yahoo.com](mailto:oluyale@yahoo.com); [216075659@stu.ukzn.ac.za](mailto:216075659@stu.ukzn.ac.za)

- Supplementary information


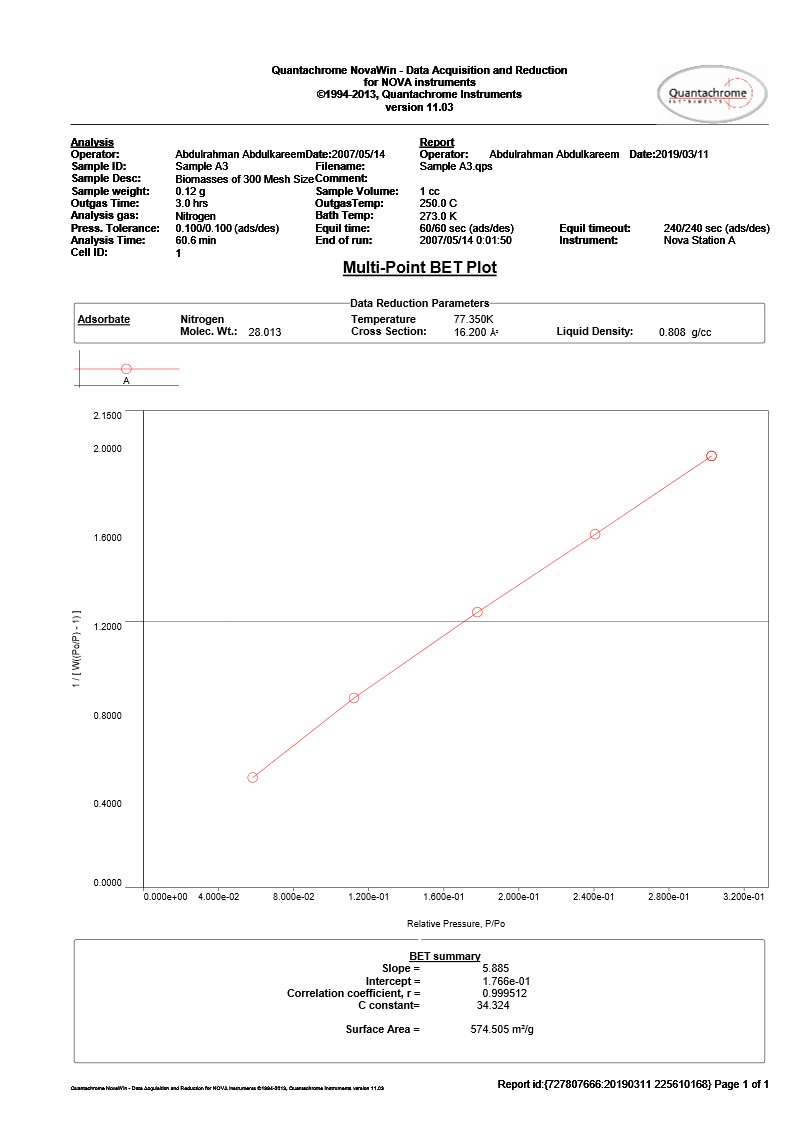

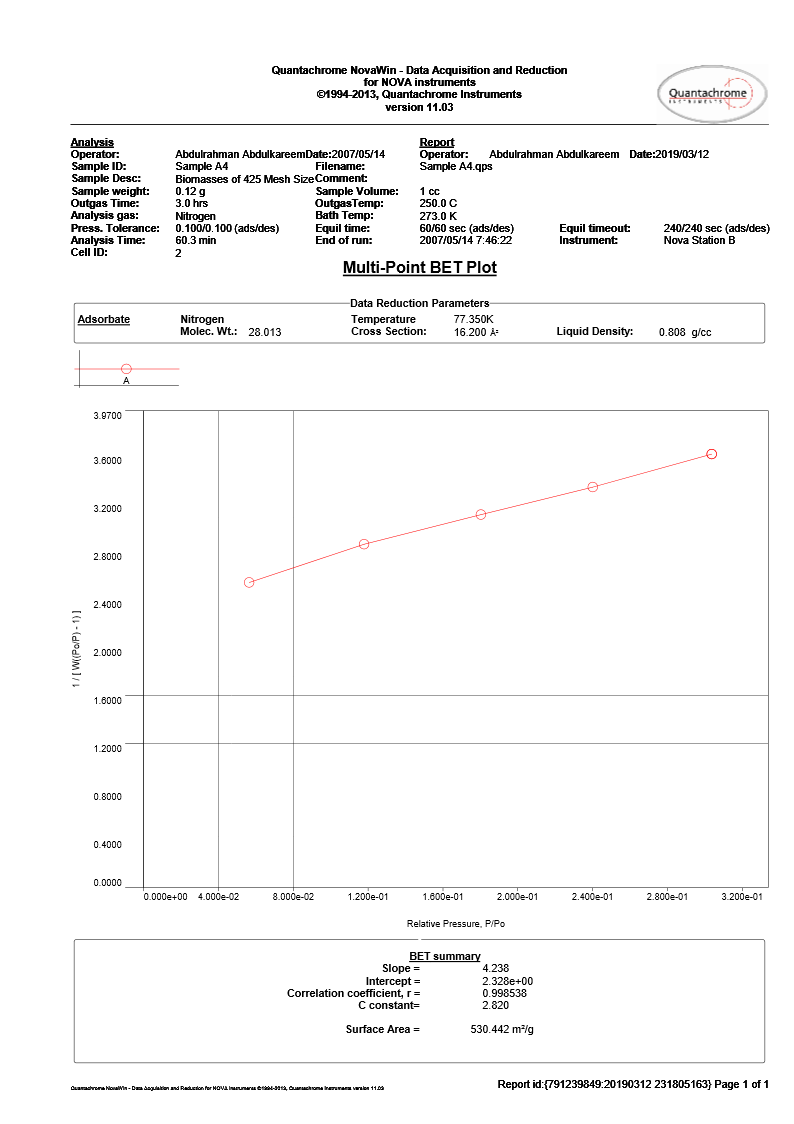


Raw Cassava peels 300 microns Raw Cassava peels 425 microns


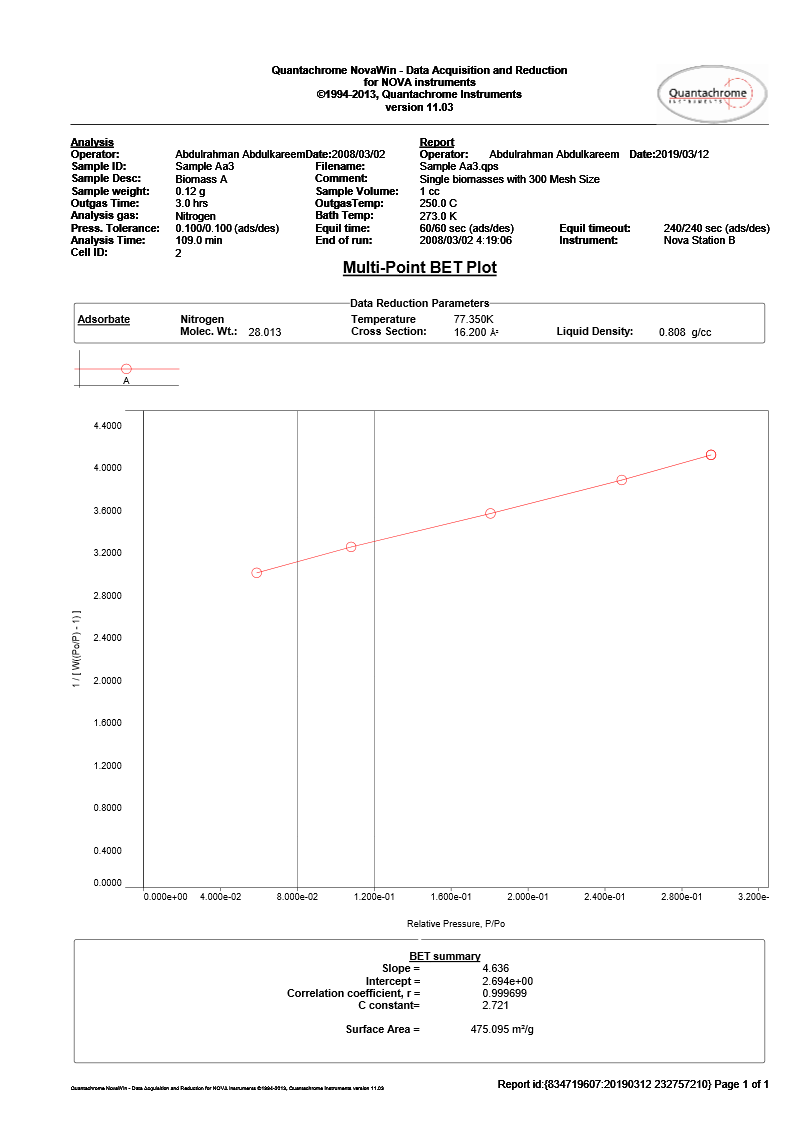

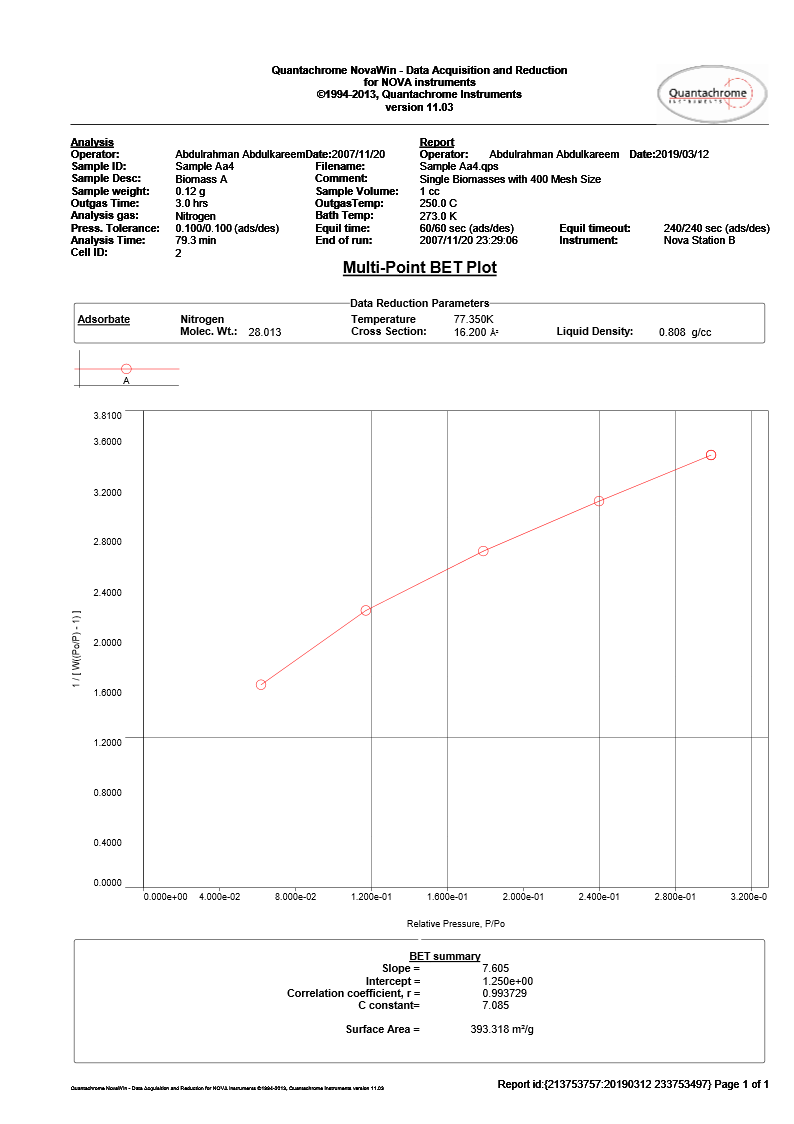


Acid pretreated Cassava peels 300 microns Acid pretreated Cassava peels 425 microns


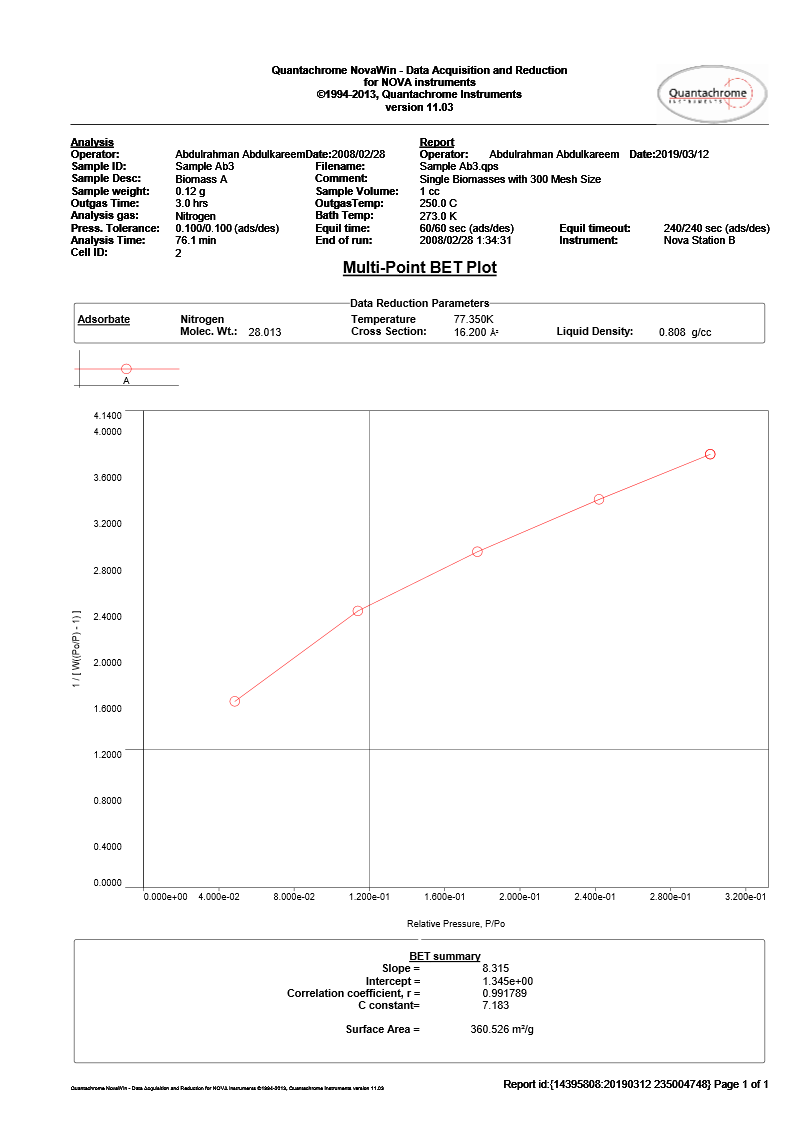

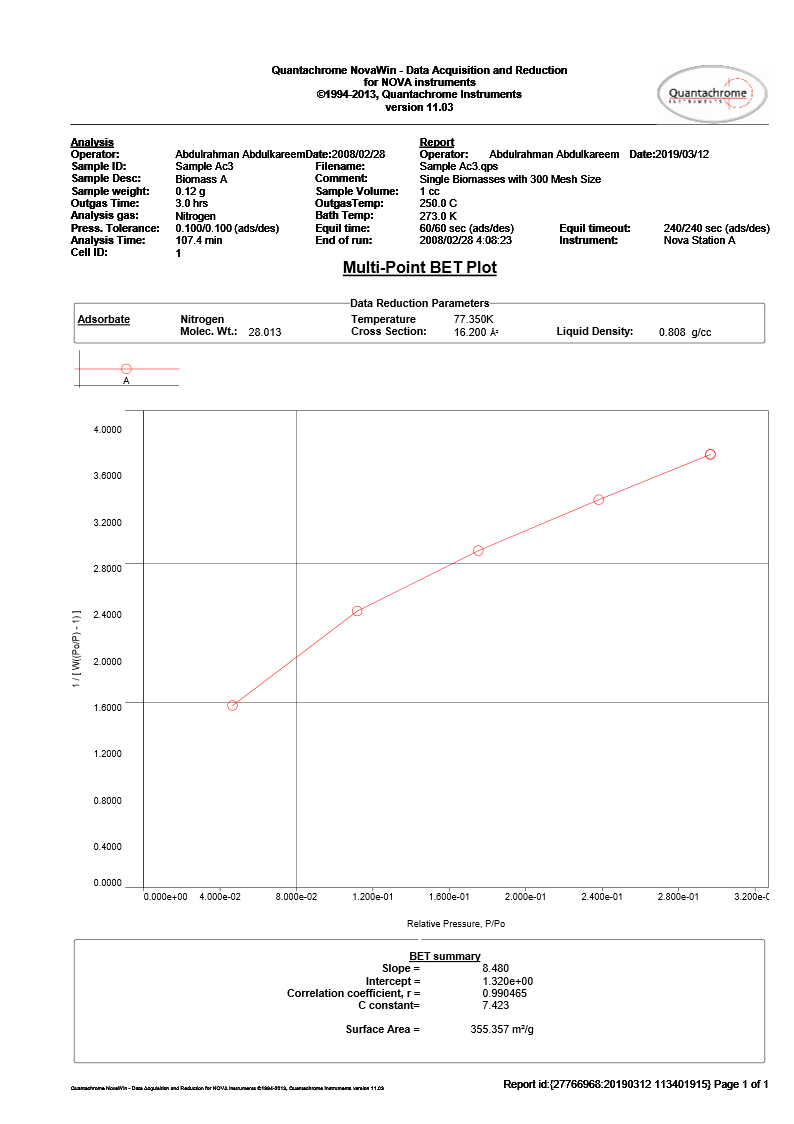


Alkali pretreated Cassava peels 300 microns Hot water pretreated cassava peels 300 microns

Figure S1: BET absorption isotherms of raw and pretreated cassava peels biomass

Figure S2: BET absorption isotherms of raw and pretreated corn cobs biomass


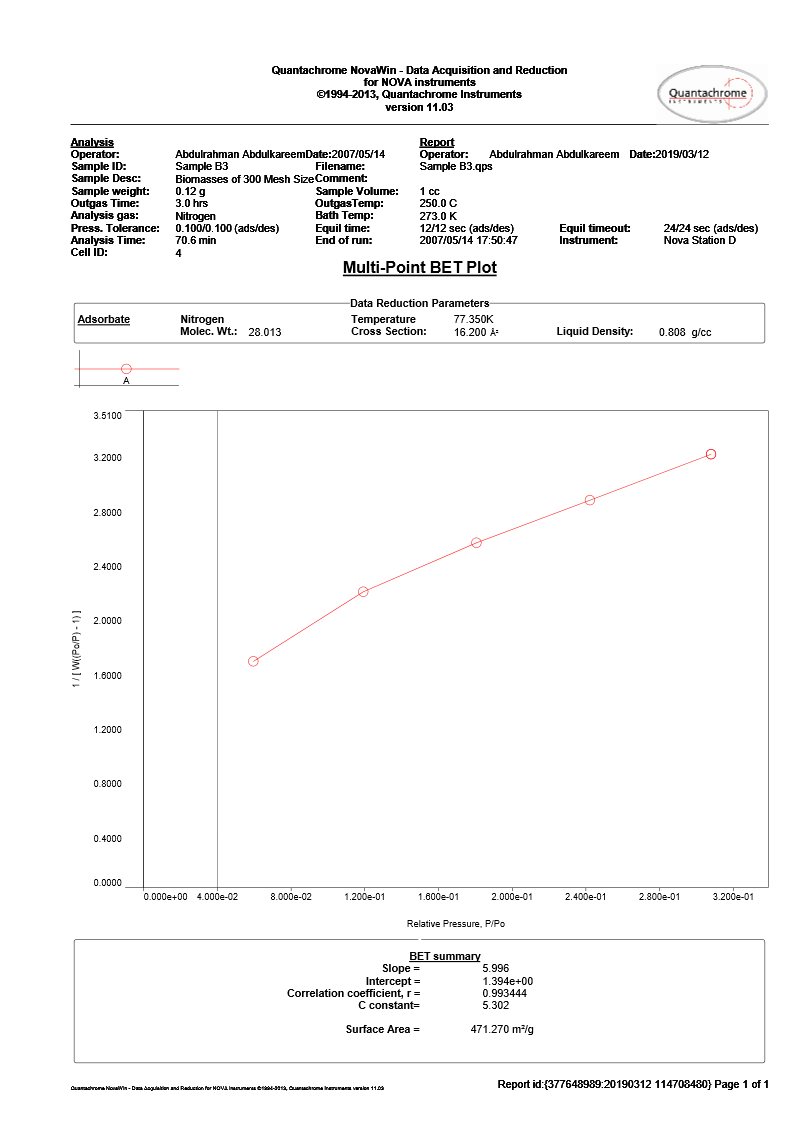

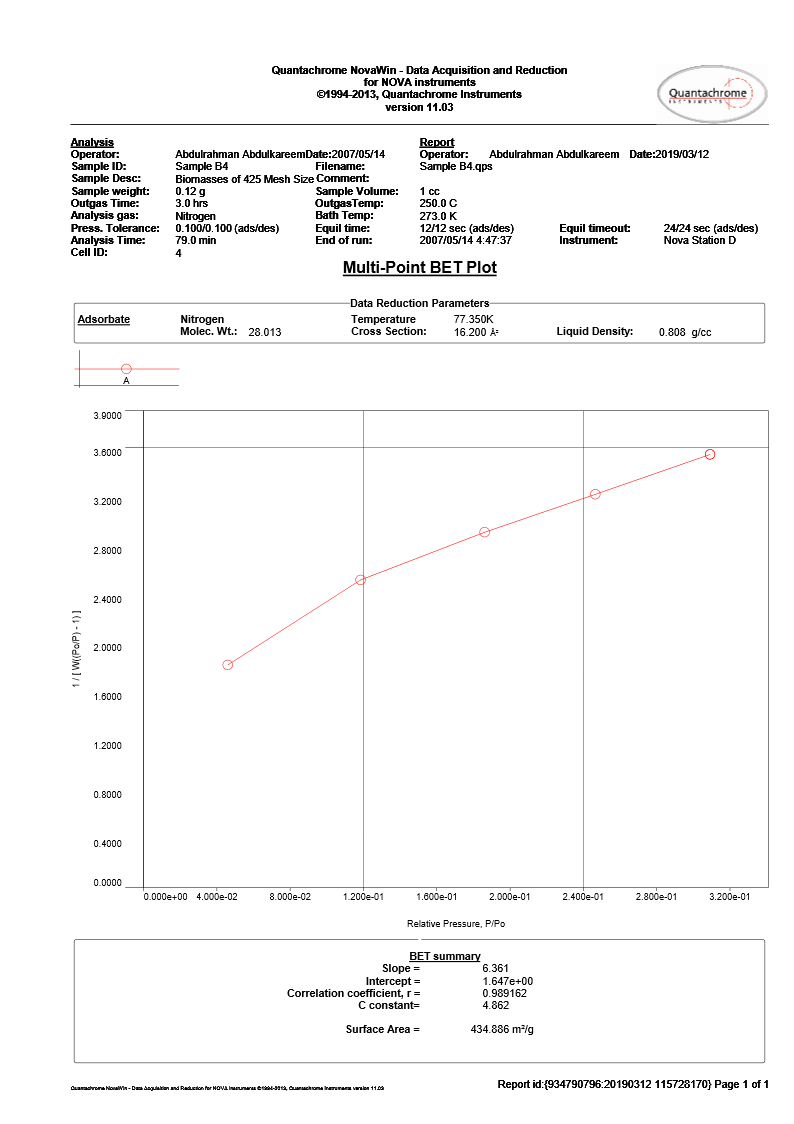


Raw corn cobs biomass 300 microns Raw corn cobs biomass 425 microns


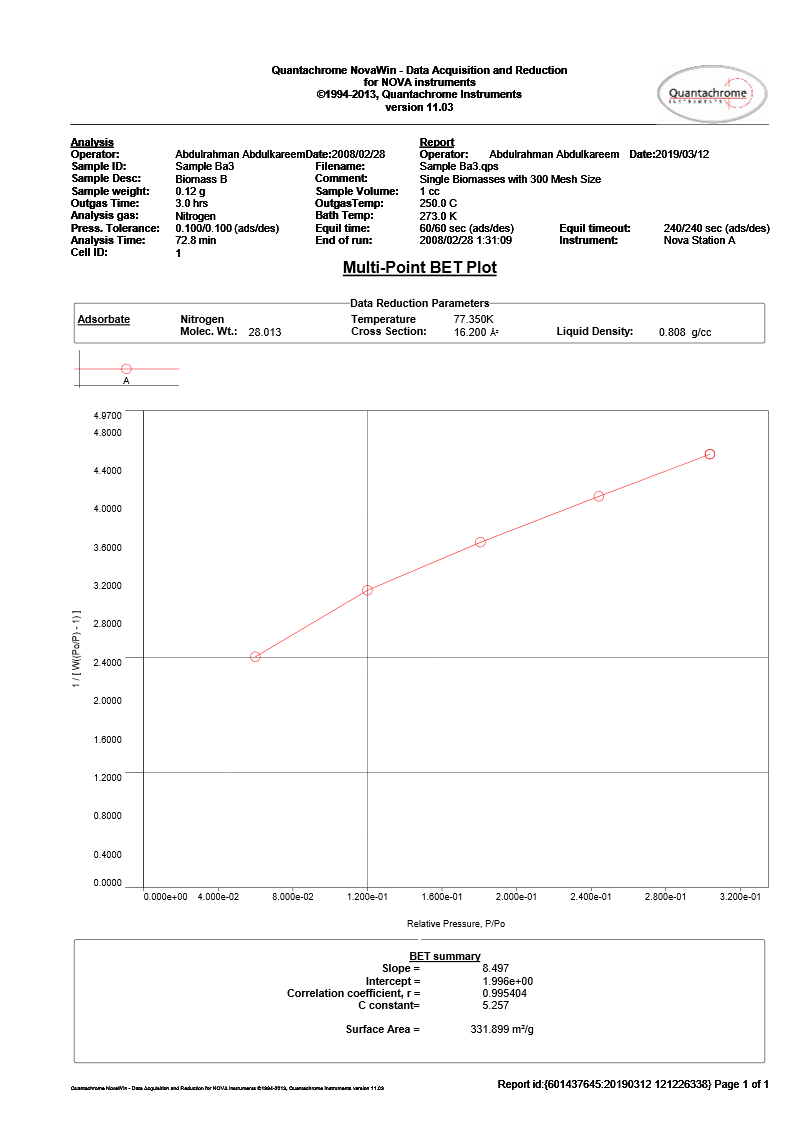

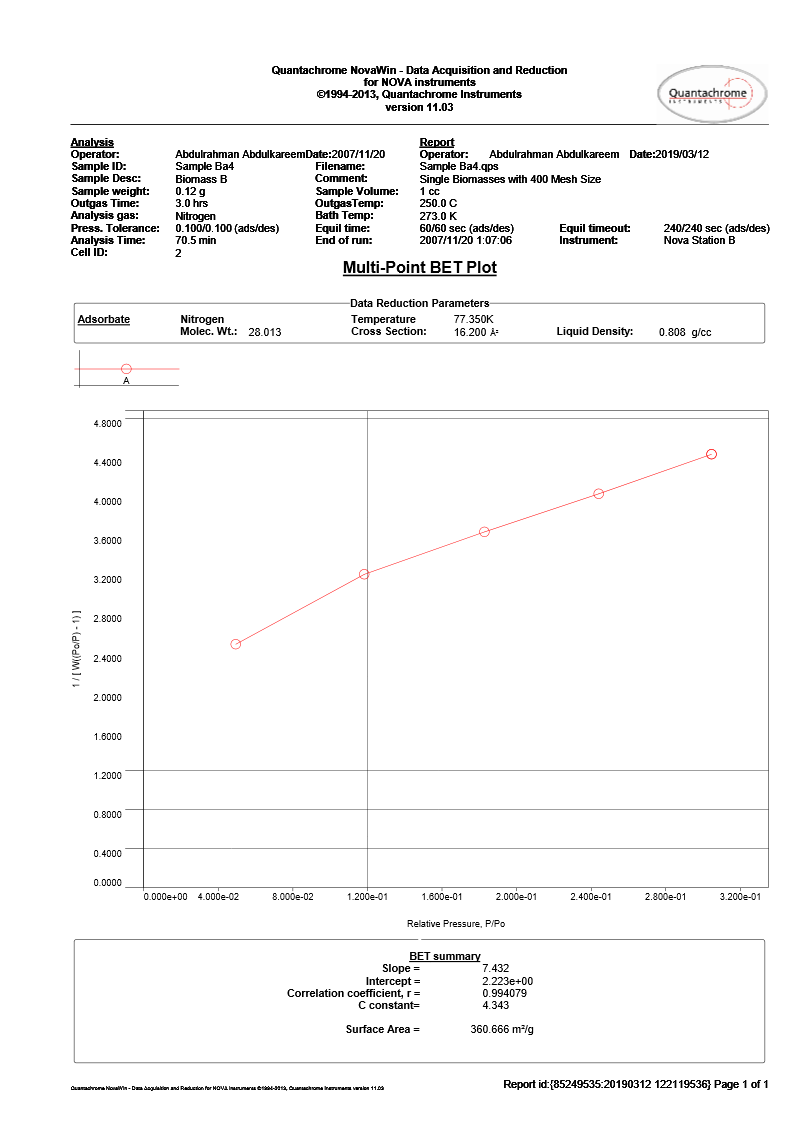


Acid pretreated corn cobs 300 microns Acid pretreated corn cobs 425 microns


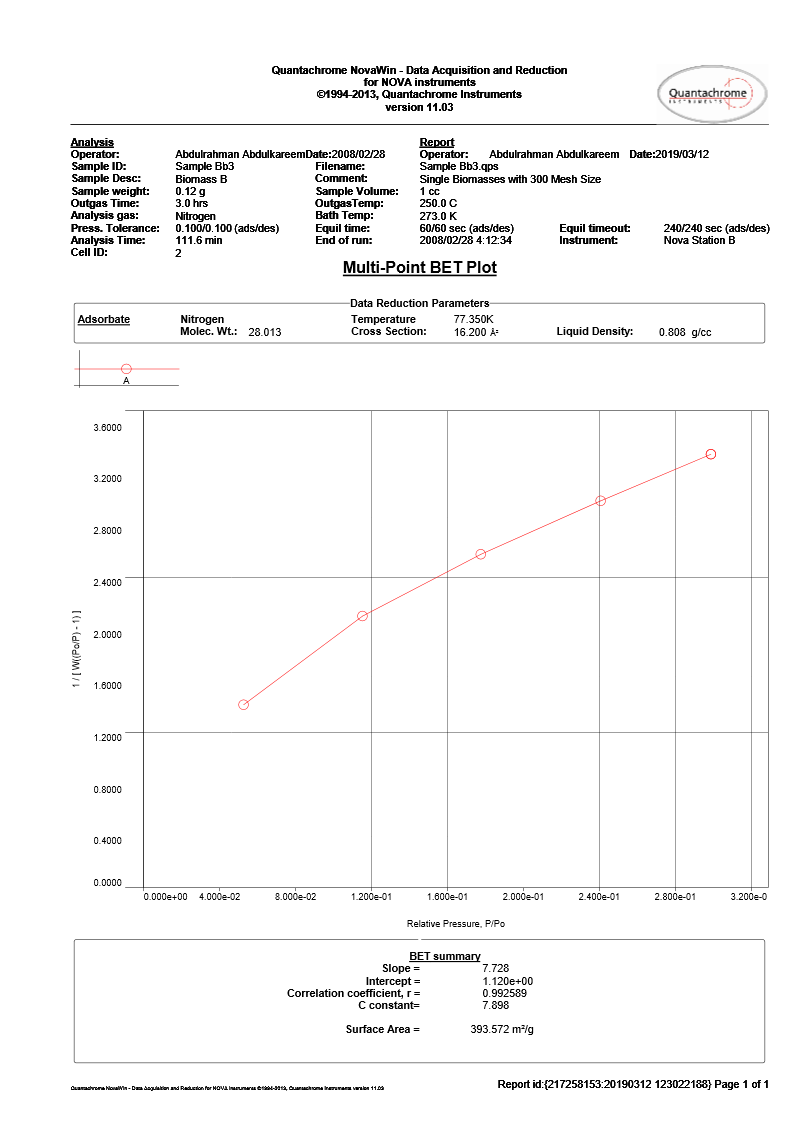

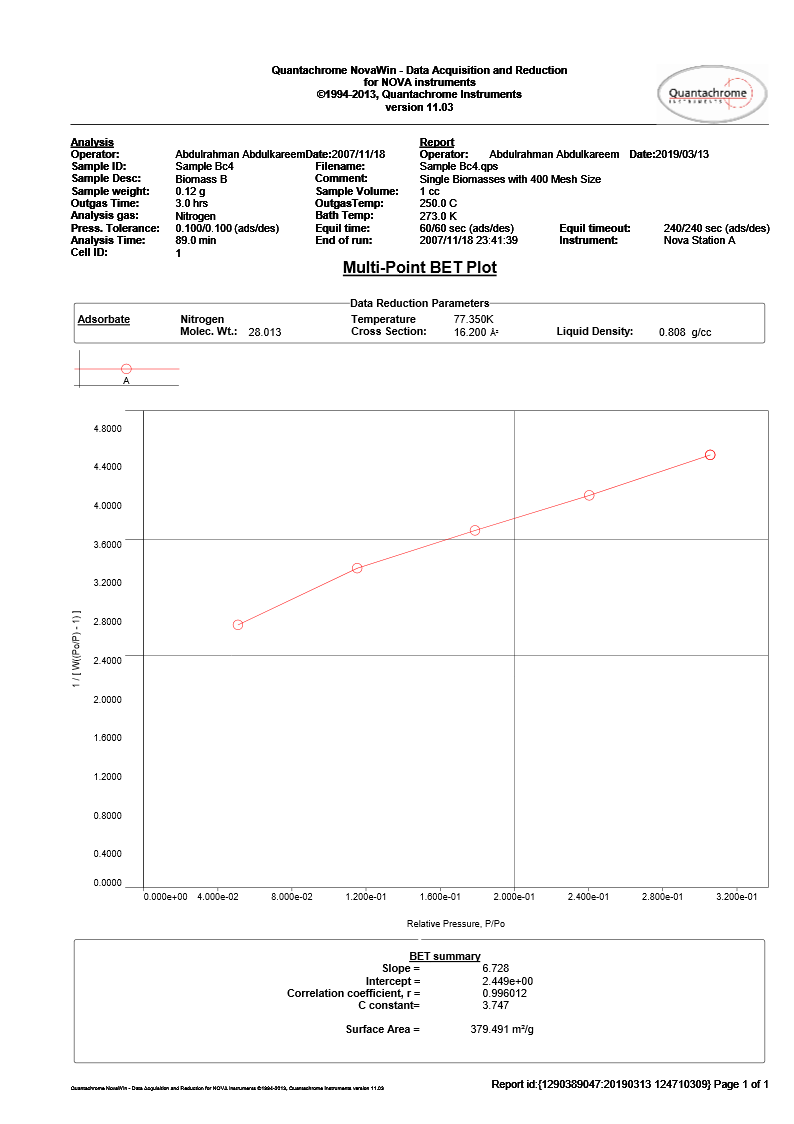


Alkali pretreated corn cobs 300 microns Hot water pretreated corn cobs 300 microns

Figure S3: BET absorption isotherms of raw and pretreated rice husks biomass


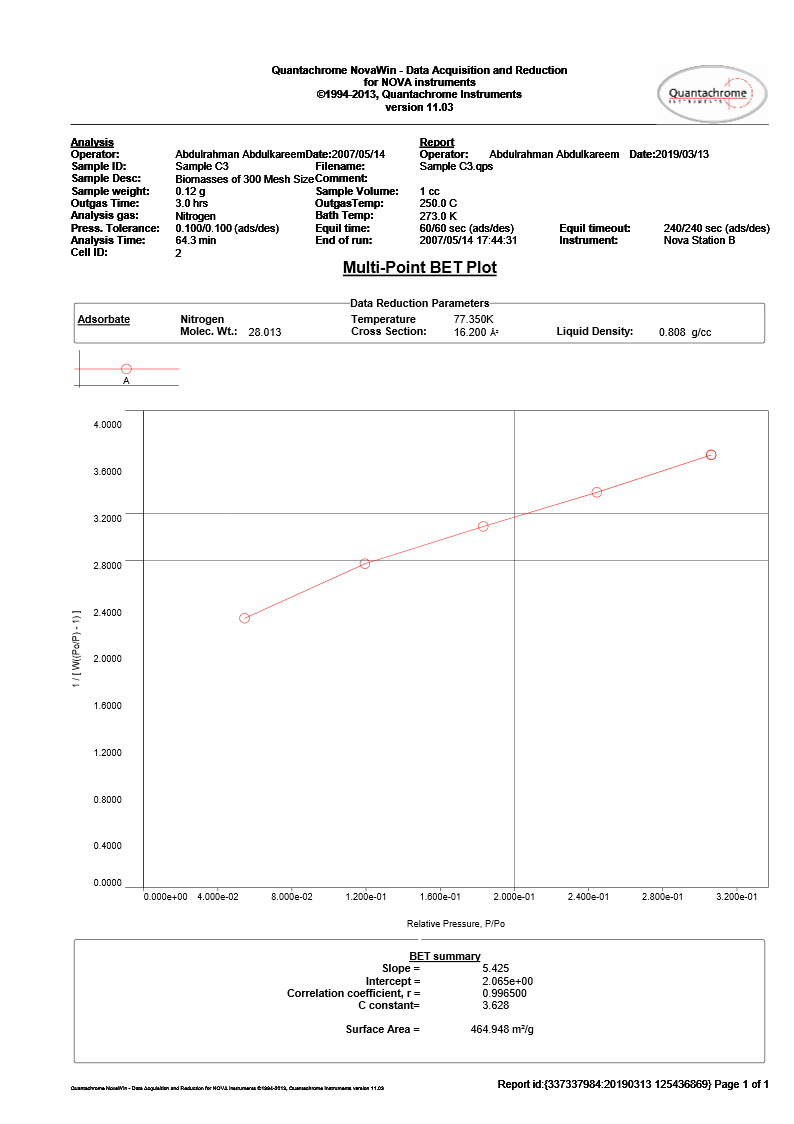

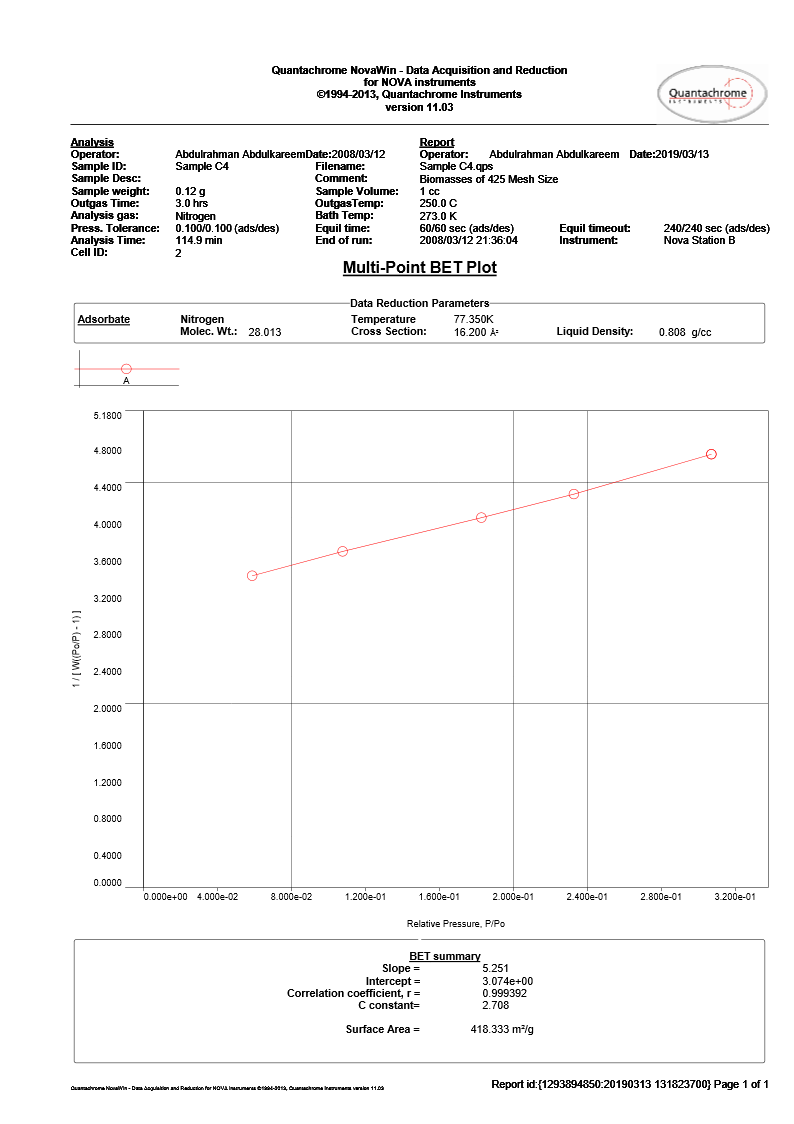


Raw rice husks 300 microns Raw rice husks 425 microns


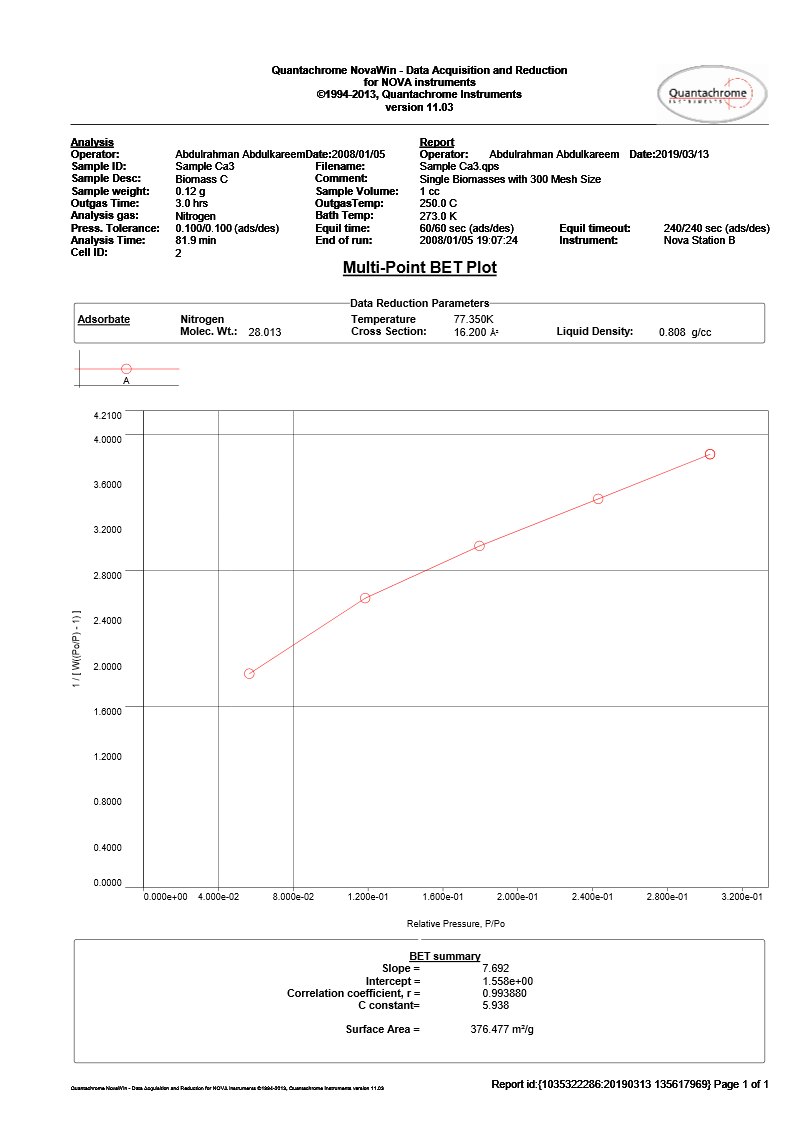

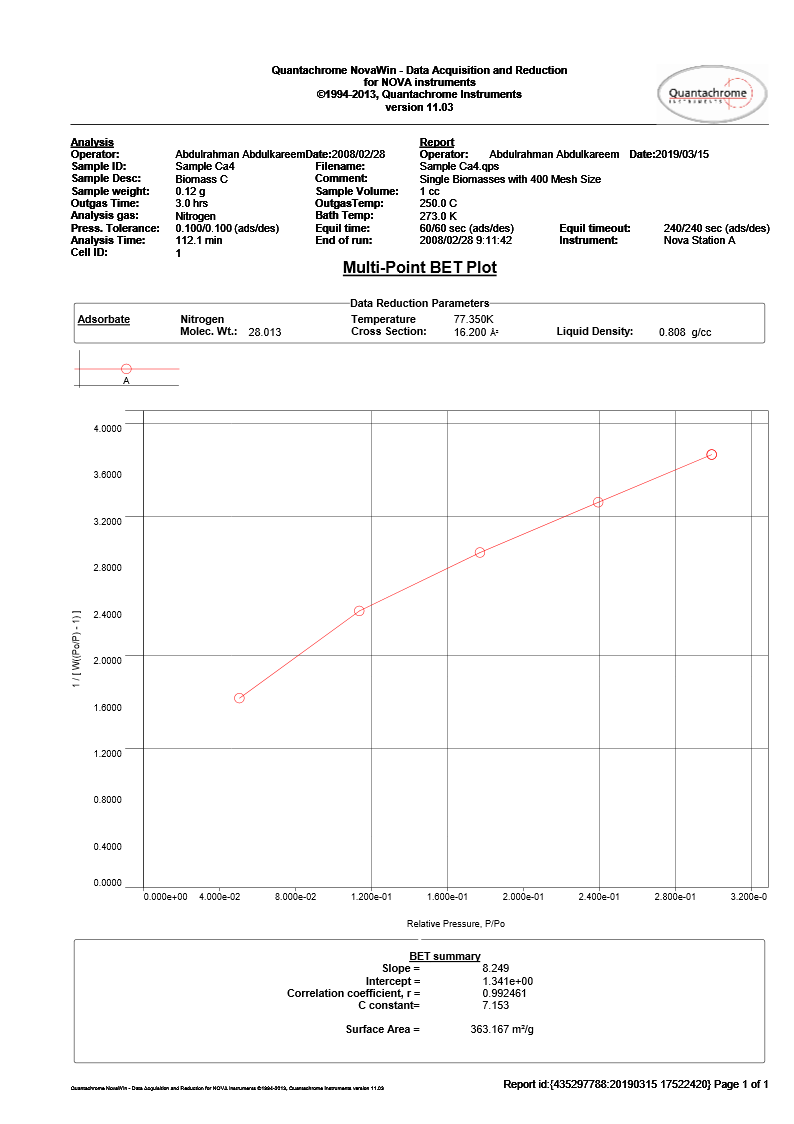


Acid pretreated rice husks biomass 300 microns Acid pretreated rice husks biomass 425 microns


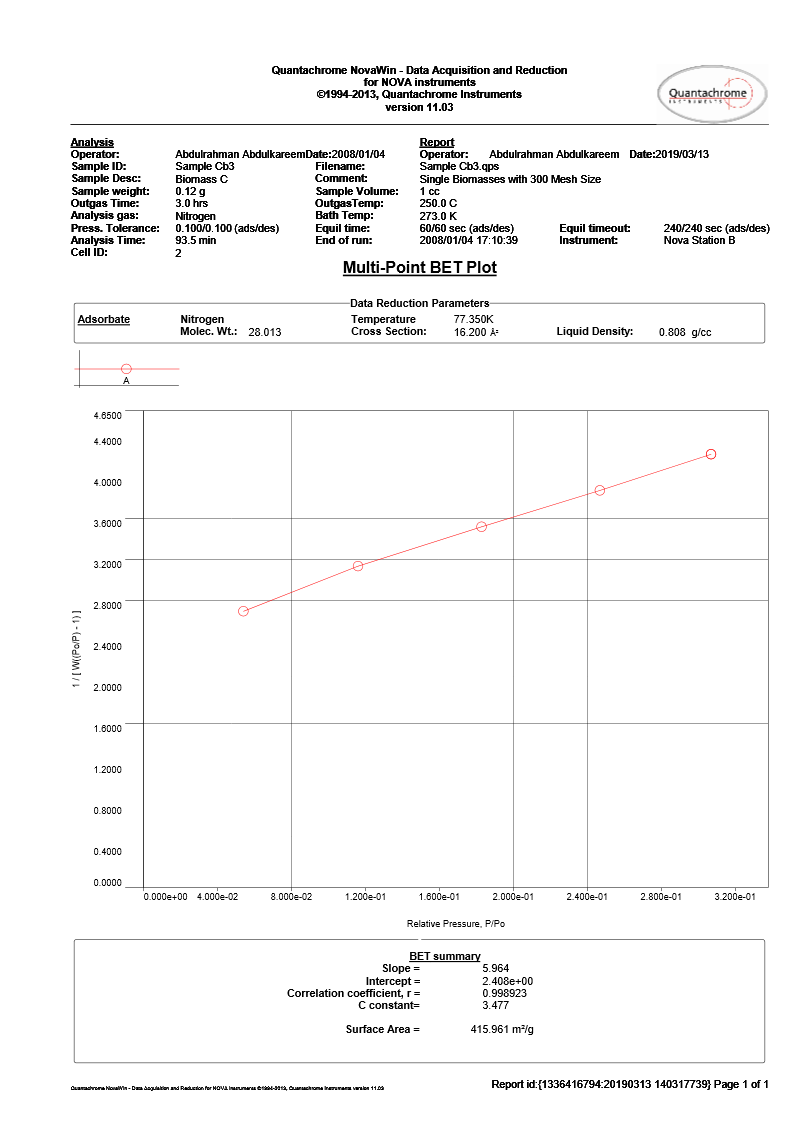

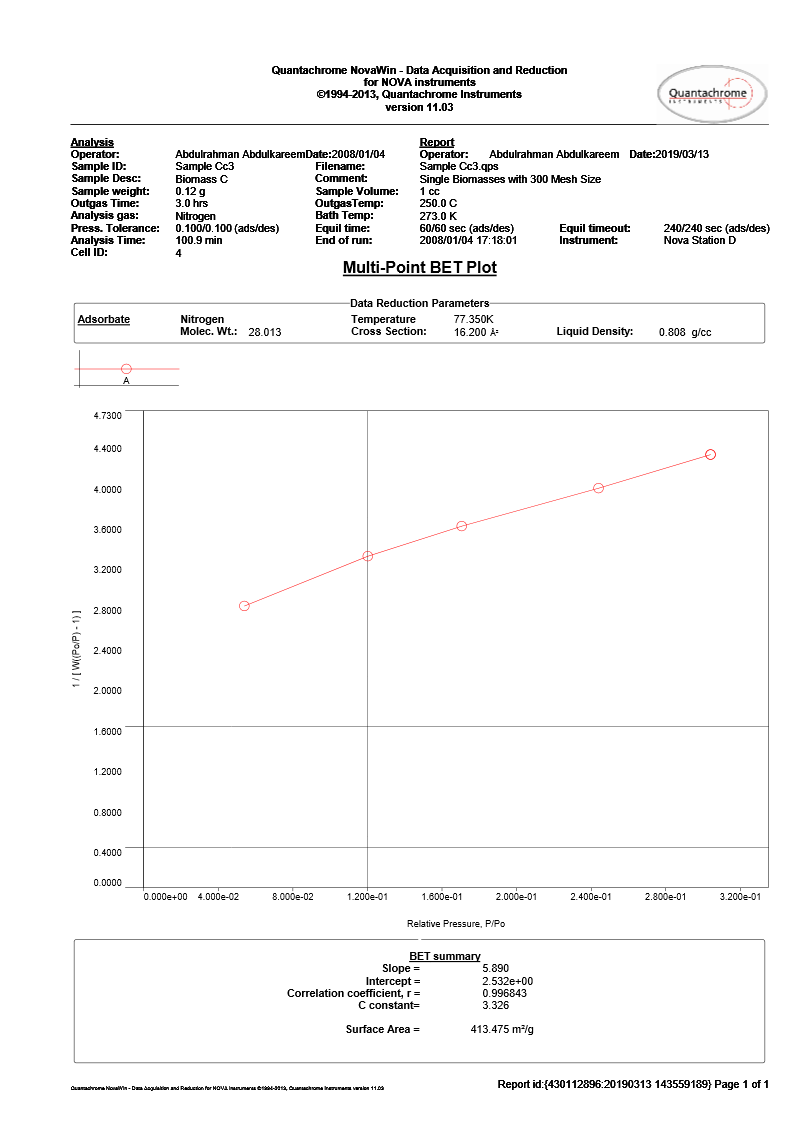


Alkali pretreated rice husks biomass 300 microns Hot water pretreated rice husks biomass 300 microns

Figure S4 : BET absorption isotherms of raw and pretreated sugar cane bagasse biomass


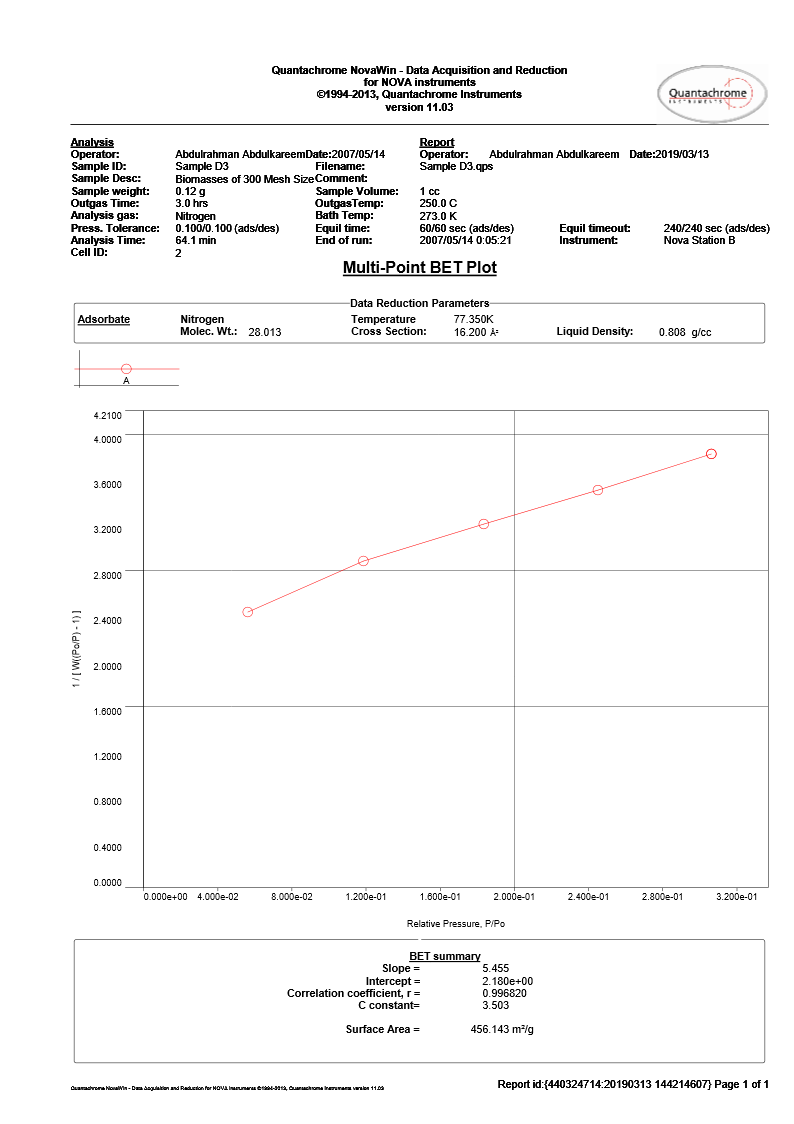

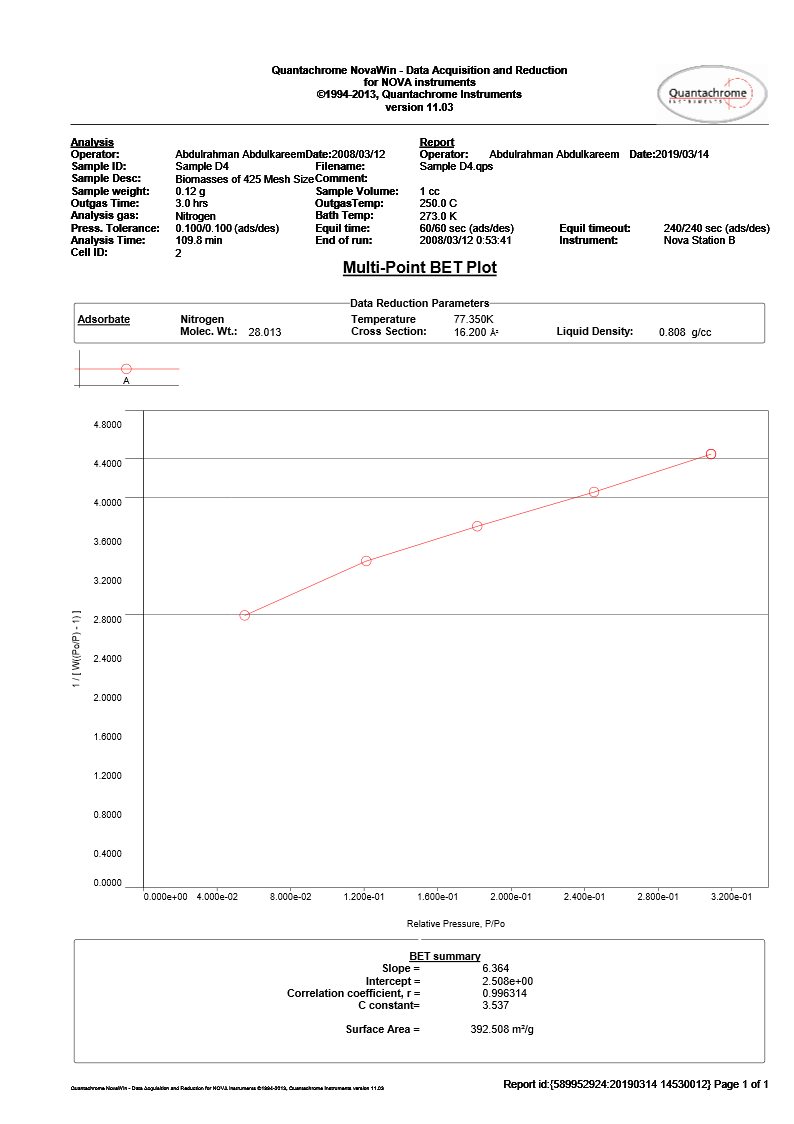


Raw sugar cane bagasse 300 micron Raw sugar cane bagasse 425 micron


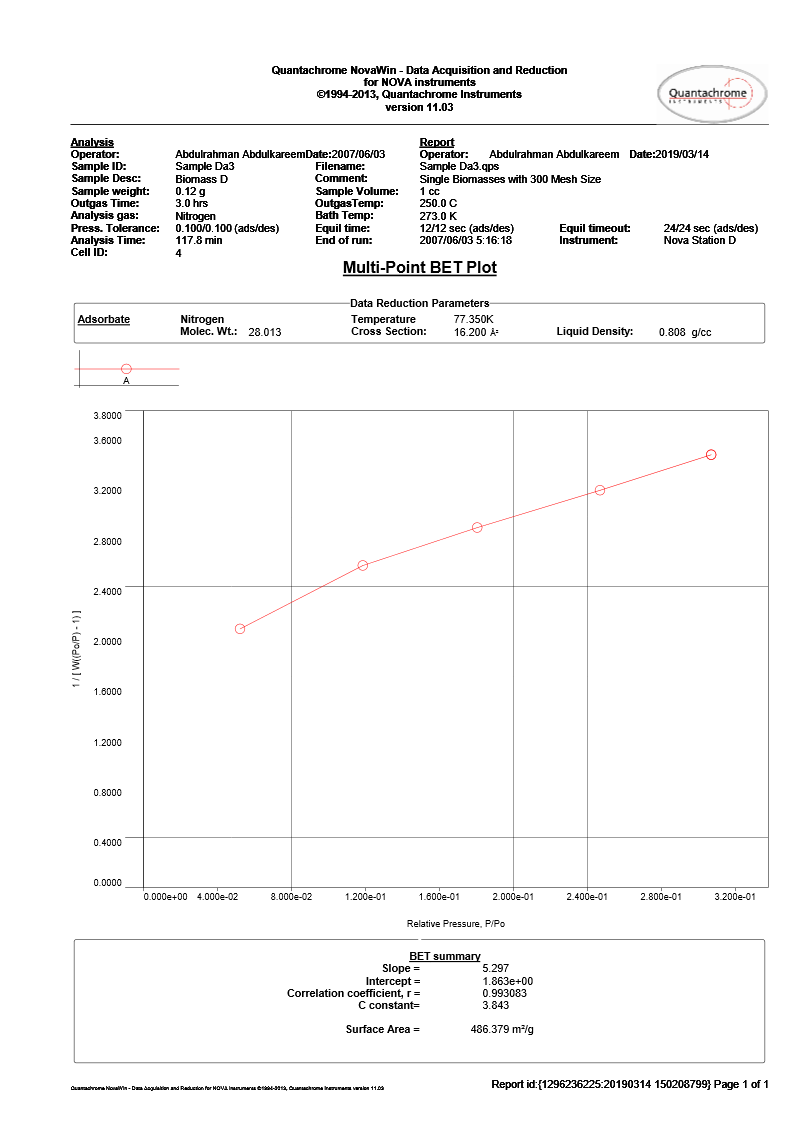

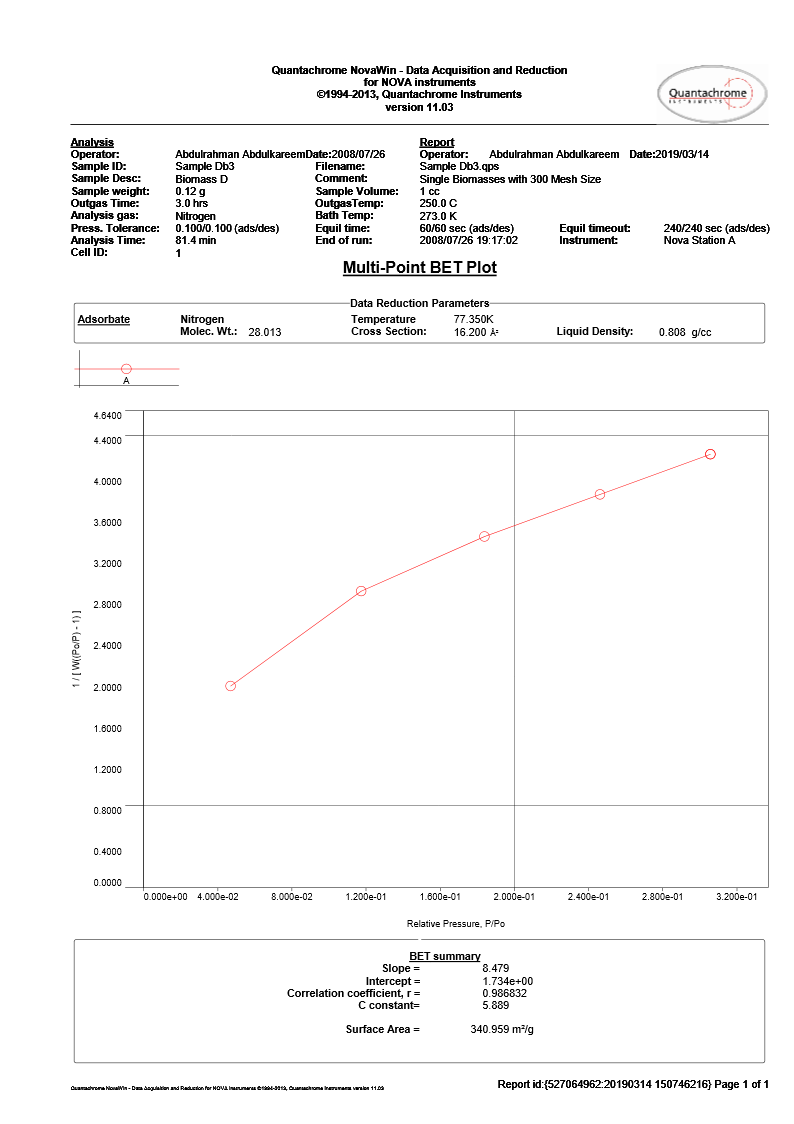


Acid pretreated sugar cane bagasse 300 microns Alkali pretreated sugar cane bagasse 300 microns


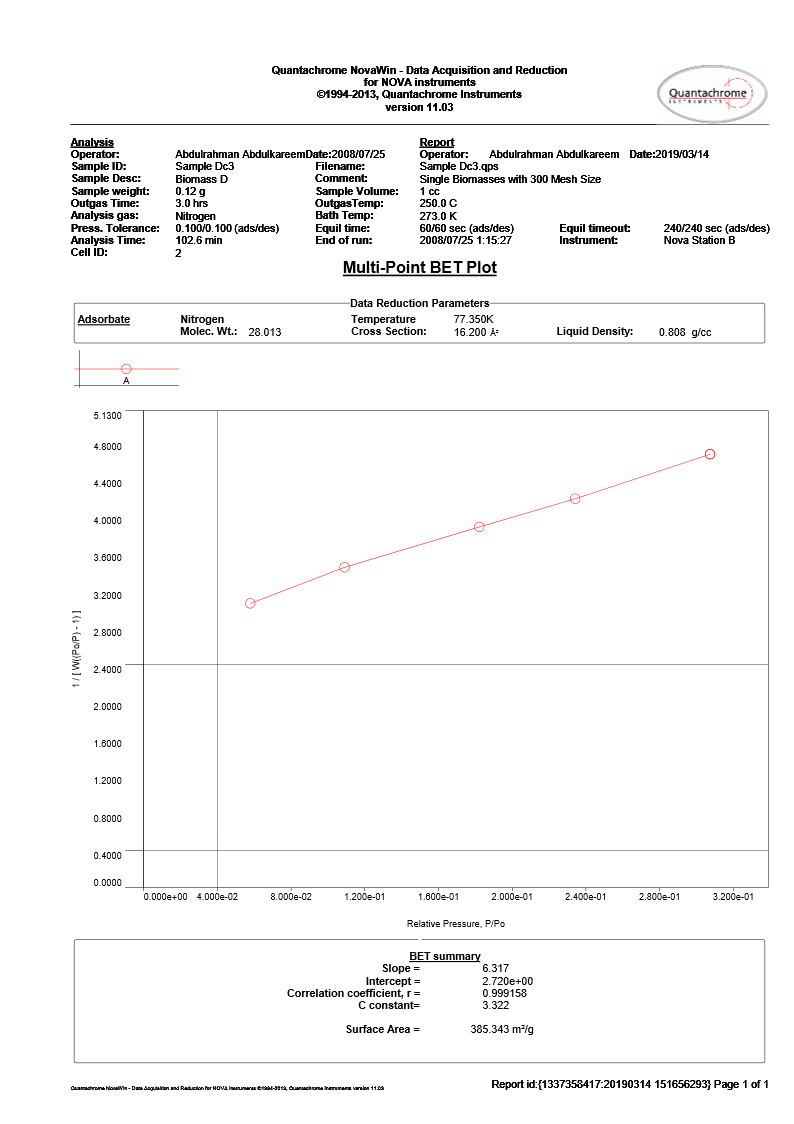


Hot water pretreated sugar cane bagasse 300 microns

Figure S5 : BET absorption isotherms of raw and pretreated yam peels biomass


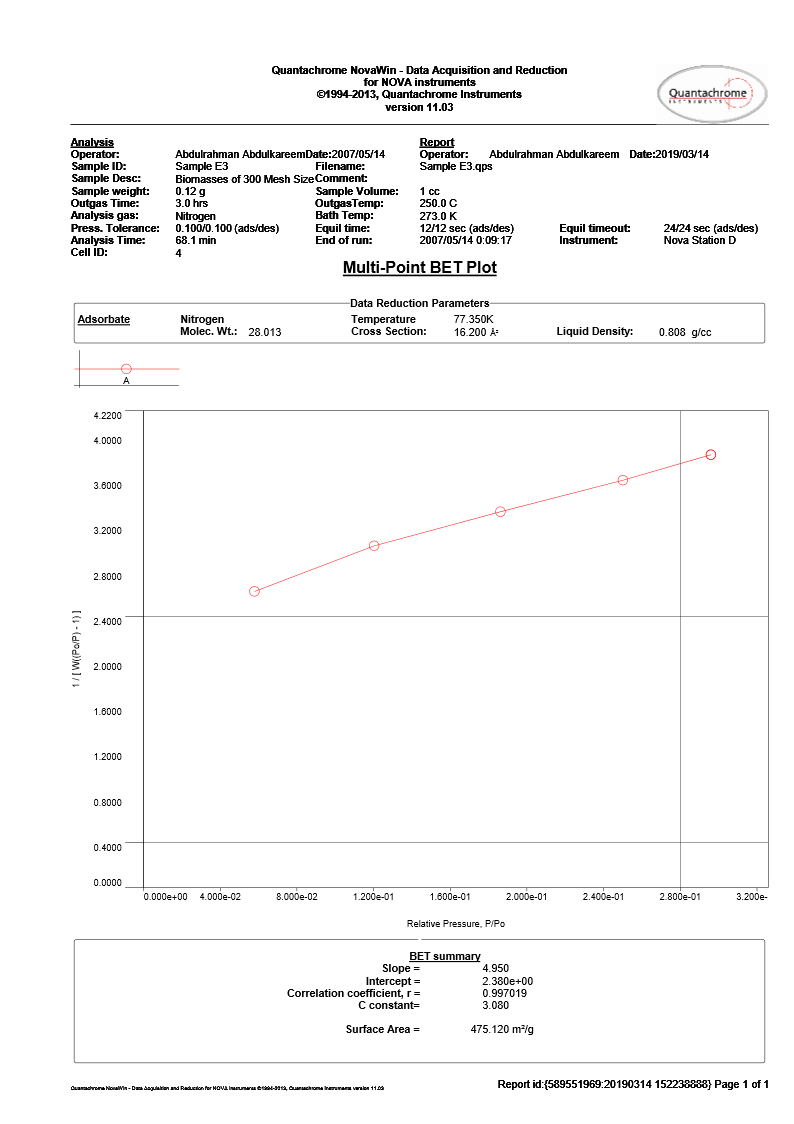

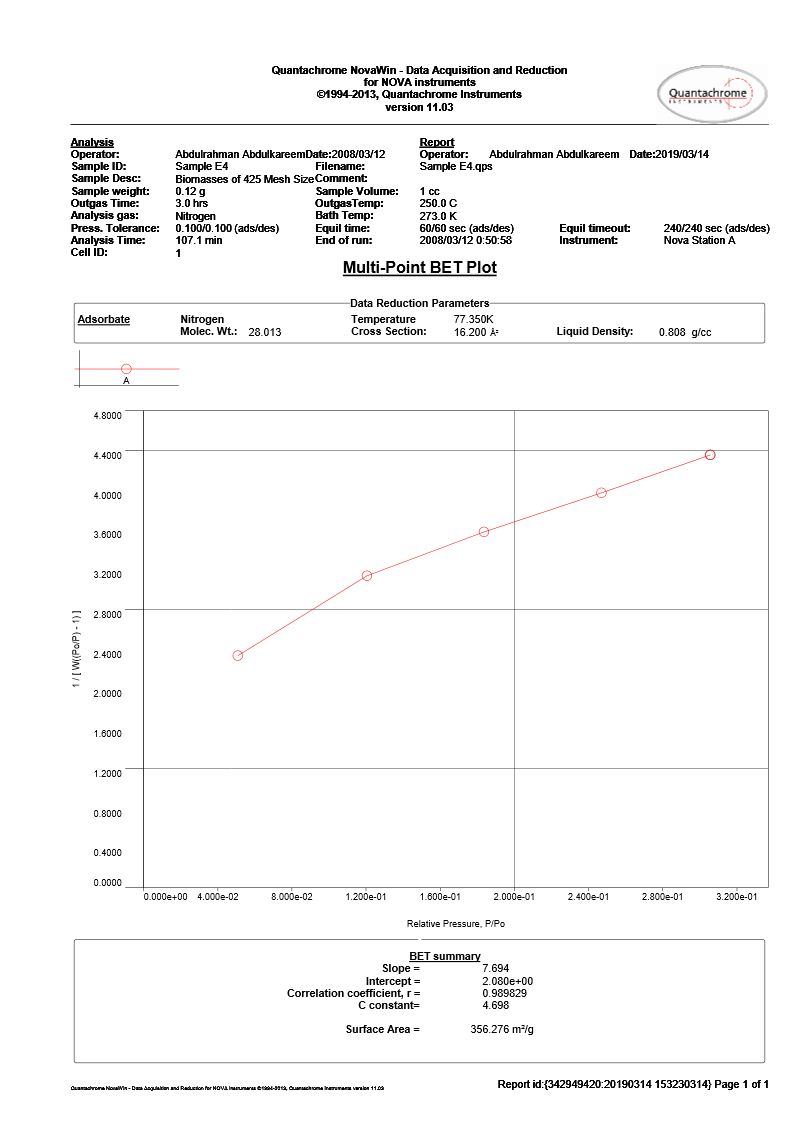


Raw yam peels biomass 300 microns Raw yam peels biomass 425 microns


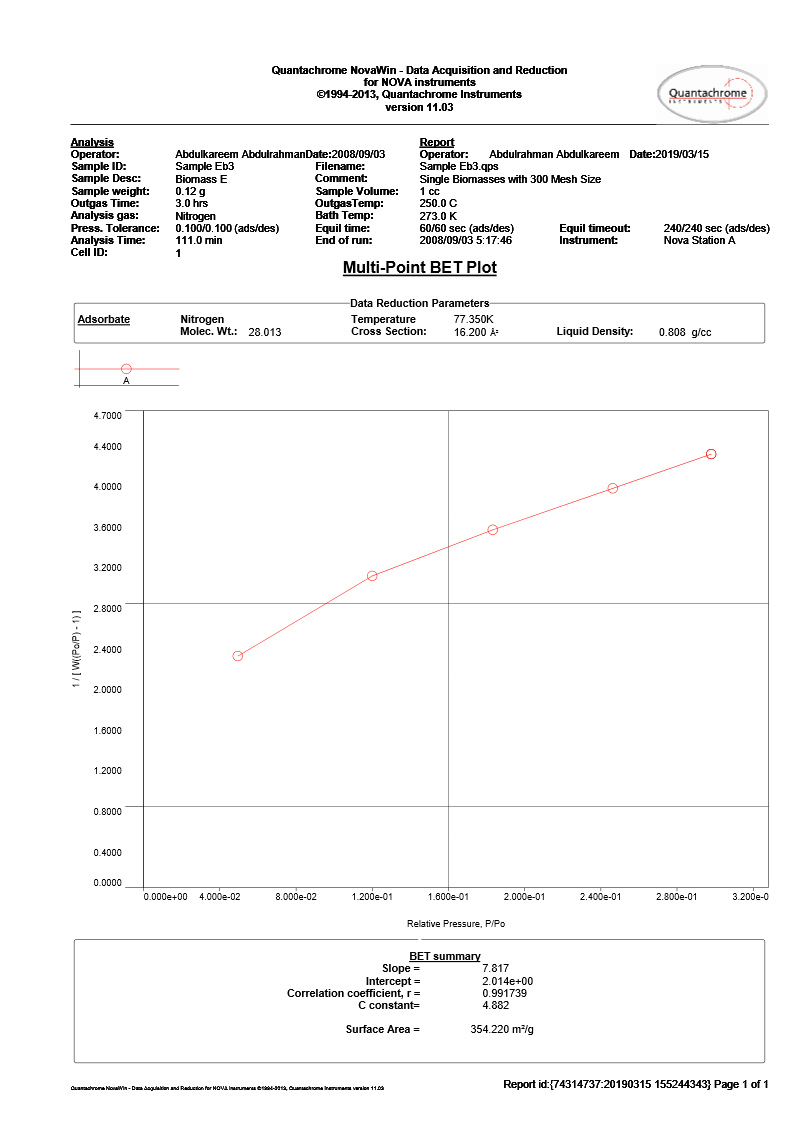

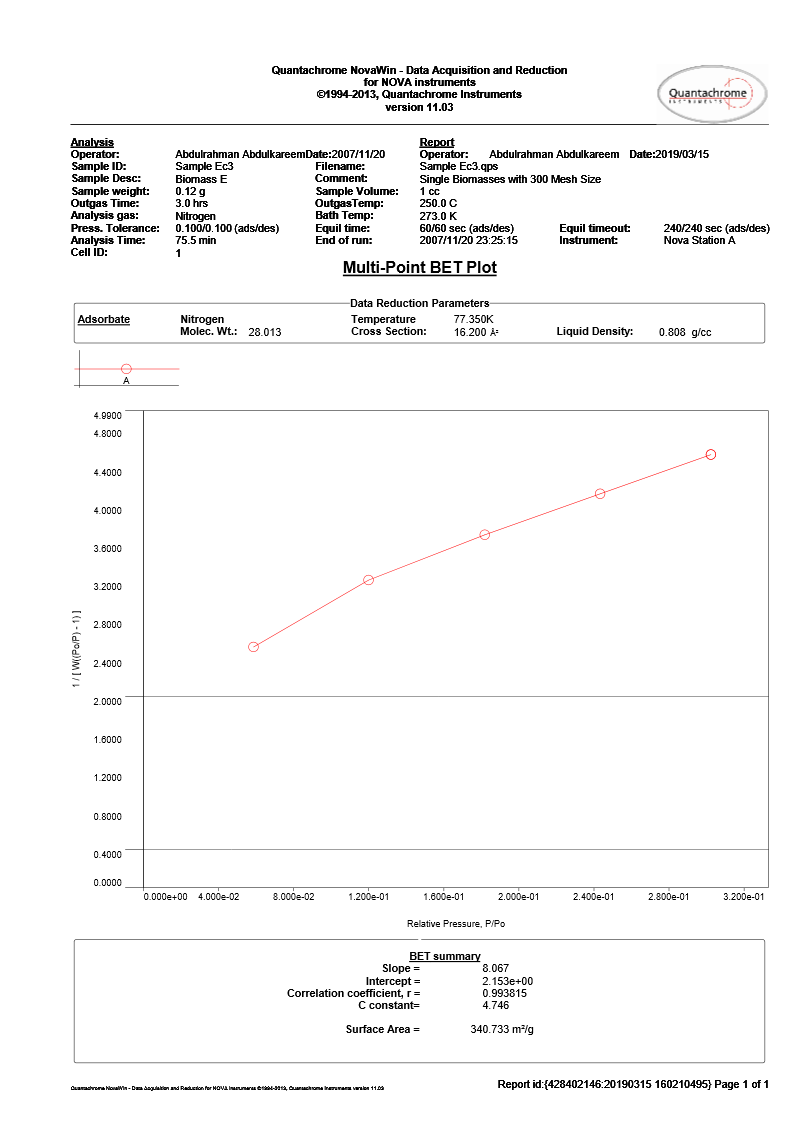


Alkali pretreated yam peels biomass 300 microns Hot water pretreated yam peels biomass 300 microns


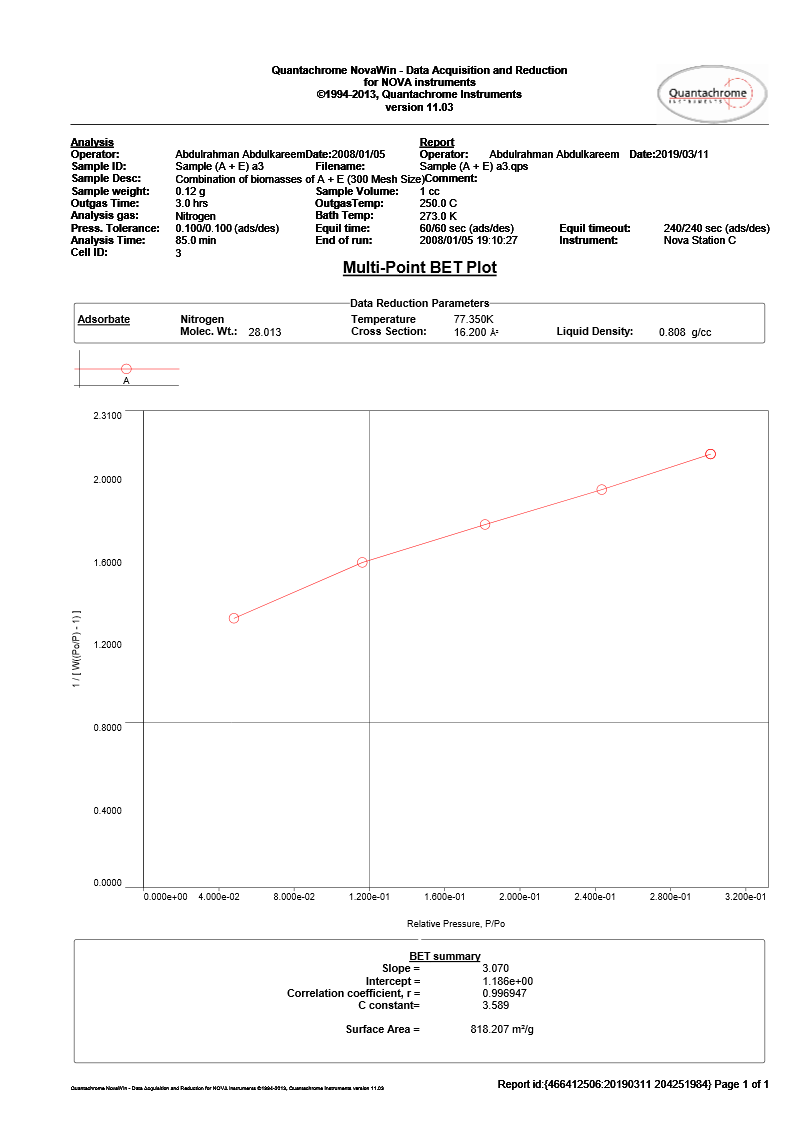

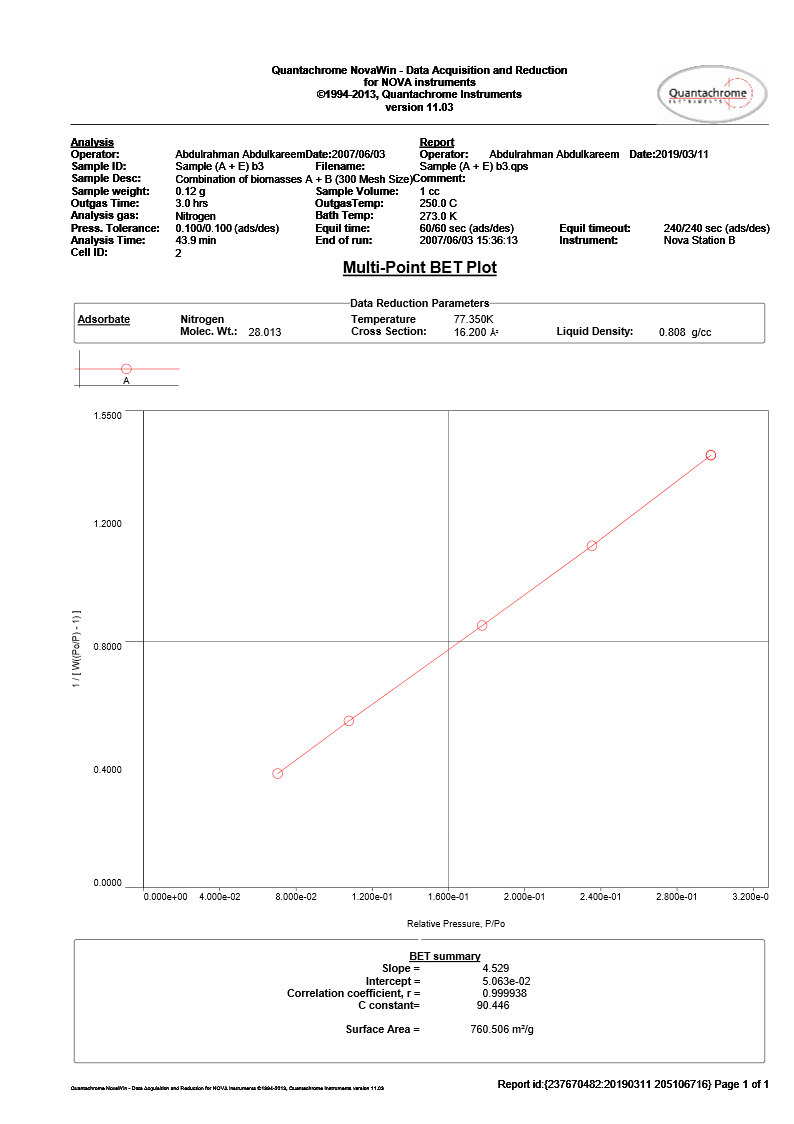


Acid pretreated 300 microns Alkali pretreated 300 microns


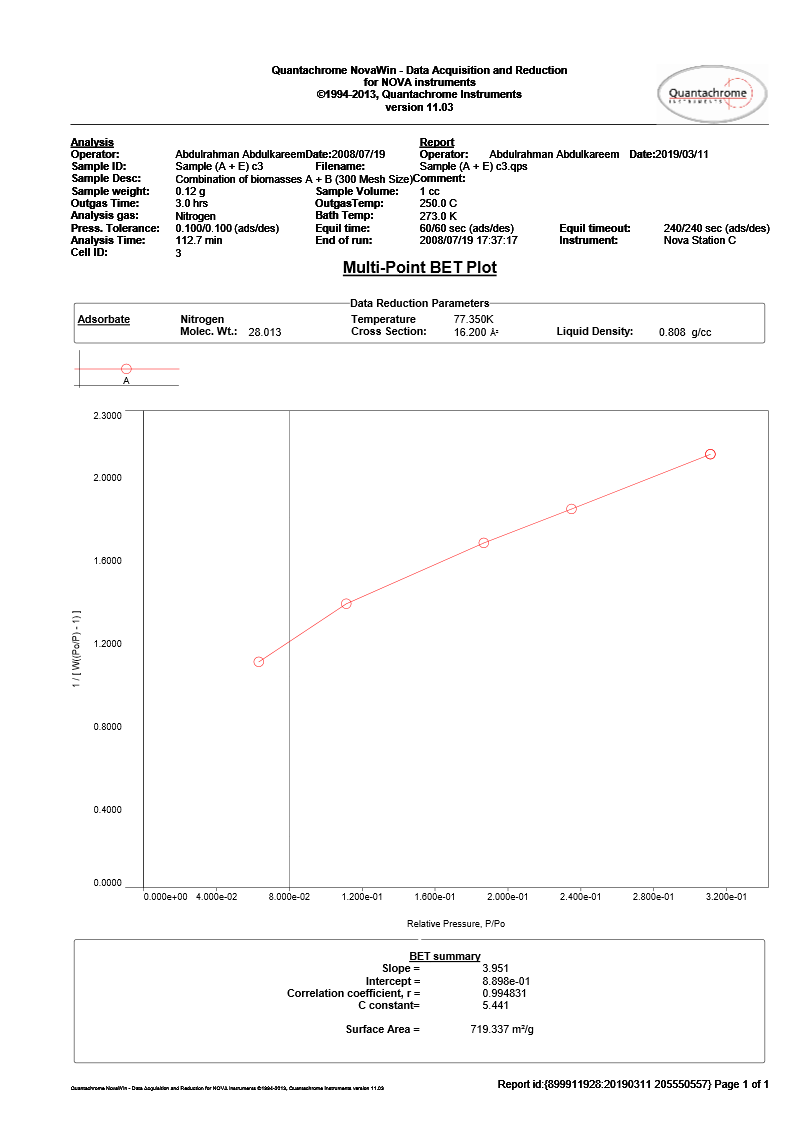


Hot water pretreated 300 microns

Figure S6 : BET absorption isotherms of pretreated cassava plus yam peels biomass


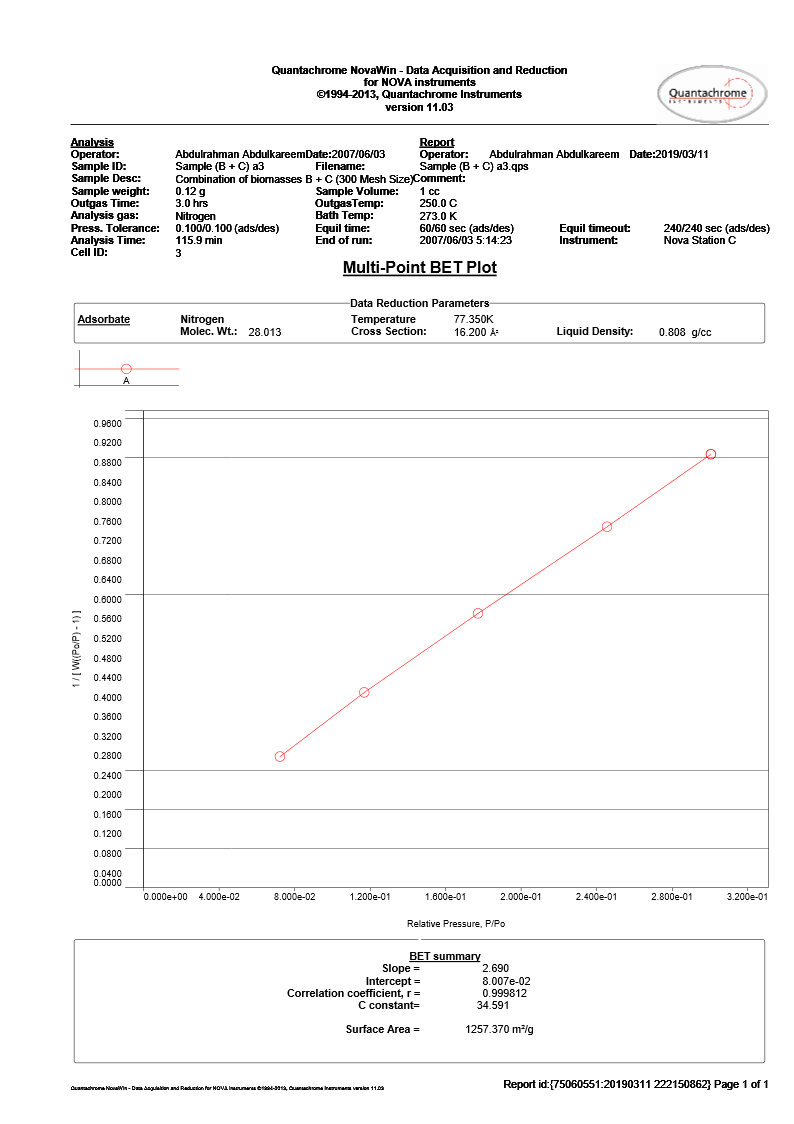

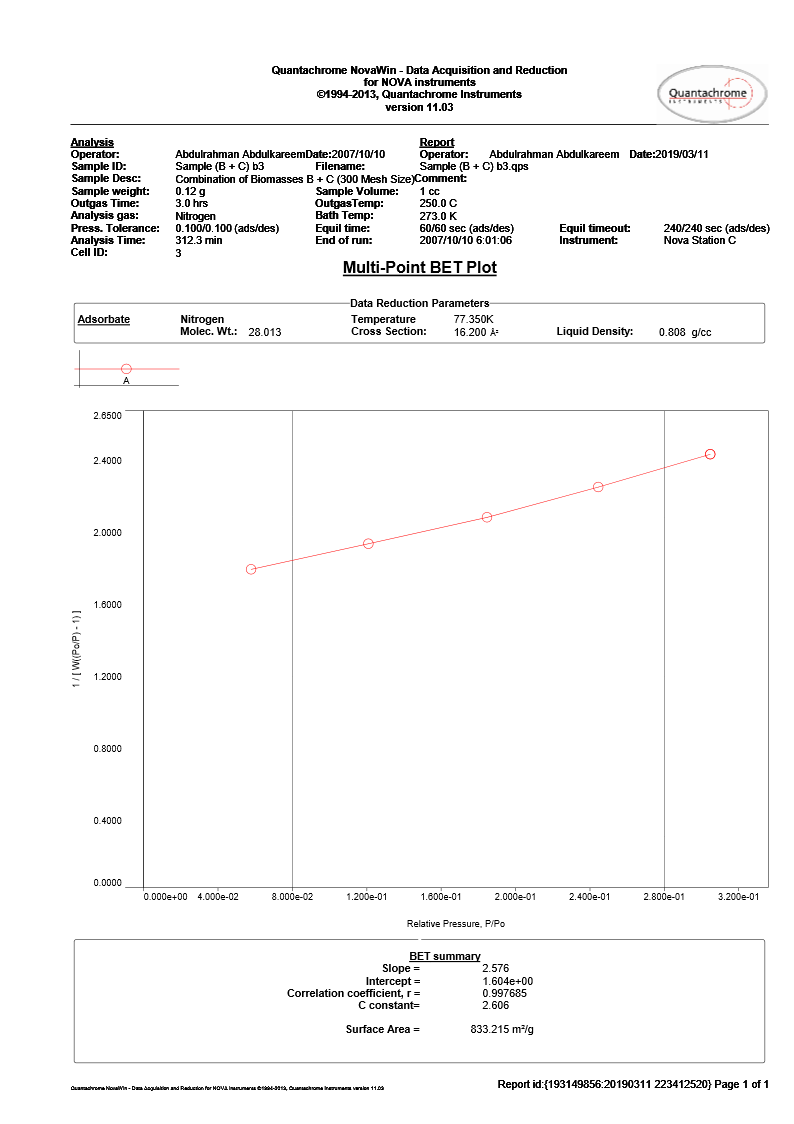


Acid pretreated Alkaline pretreated

Figure S7 : BET absorption isotherms of pretreated corn cobs plus rice husks biomass


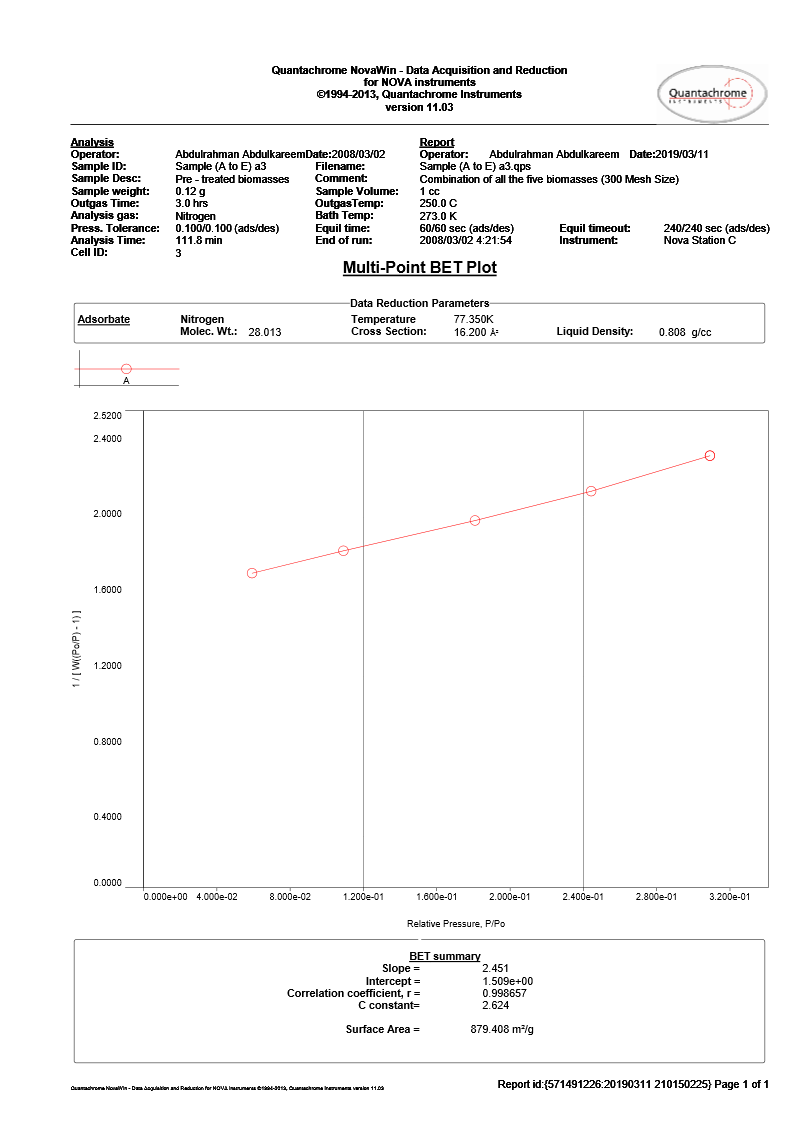

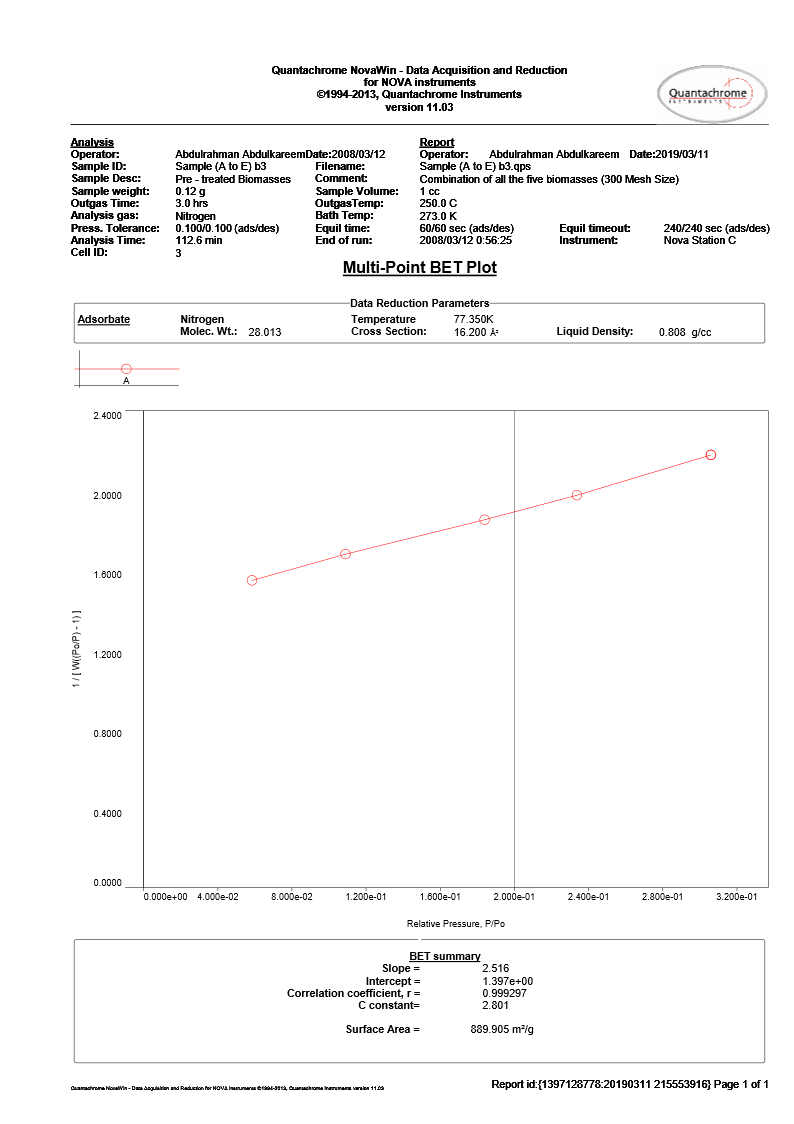


Acid pretreated Alkali pretreated


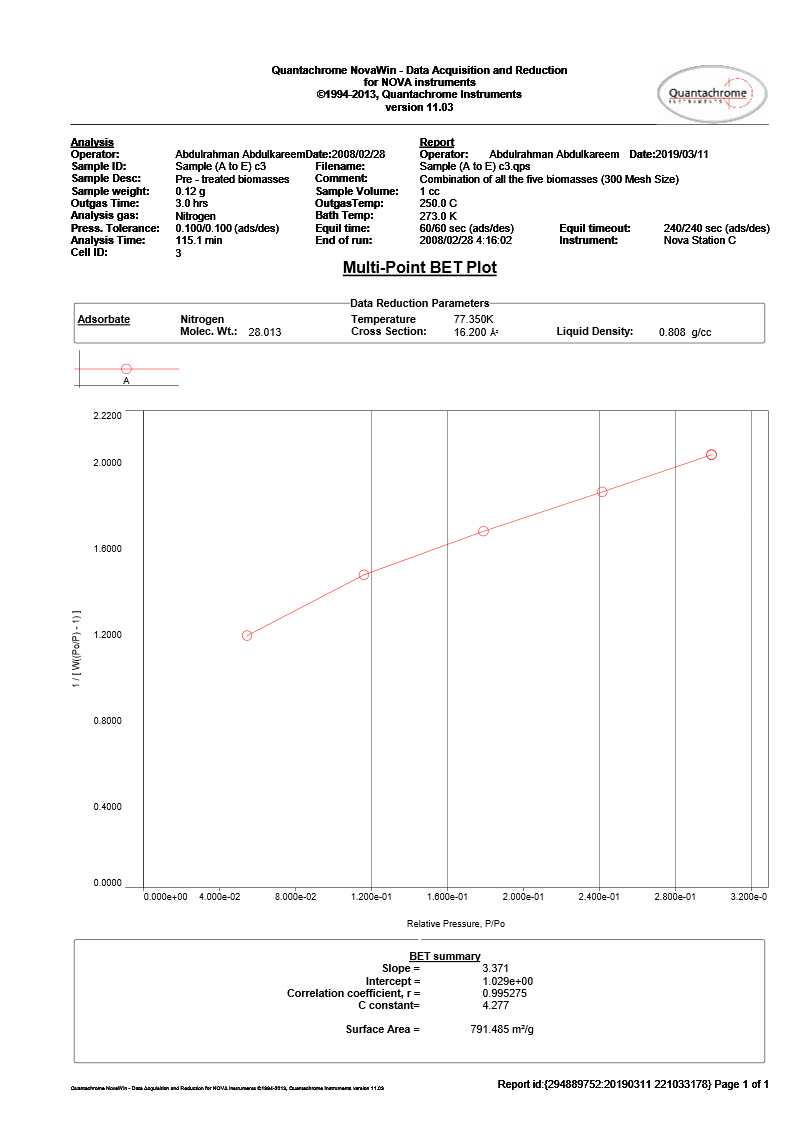


Hot water pretreated

Figure S8 : BET absorption isotherms[[26](#_ENREF_26)] of mixture of all the biomasses


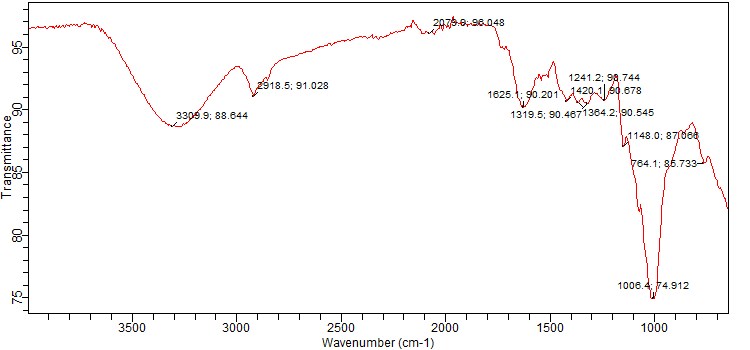

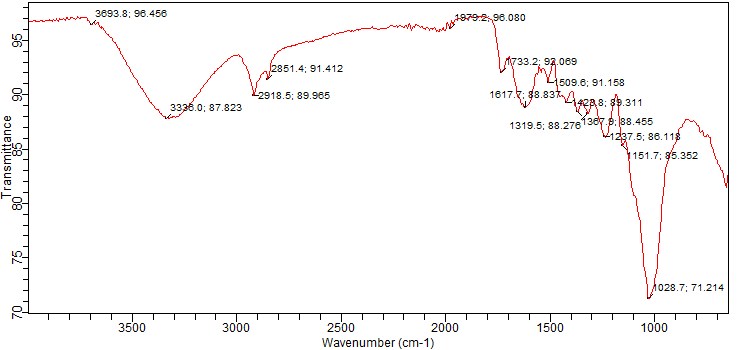


Unpretreated cassava (300 microns) Unpretreated cassava peels (425 microns)


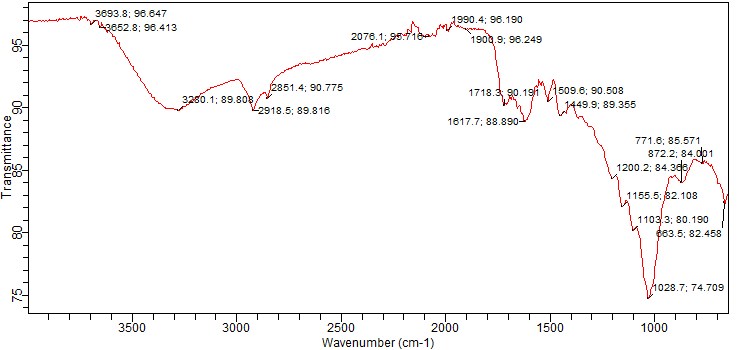

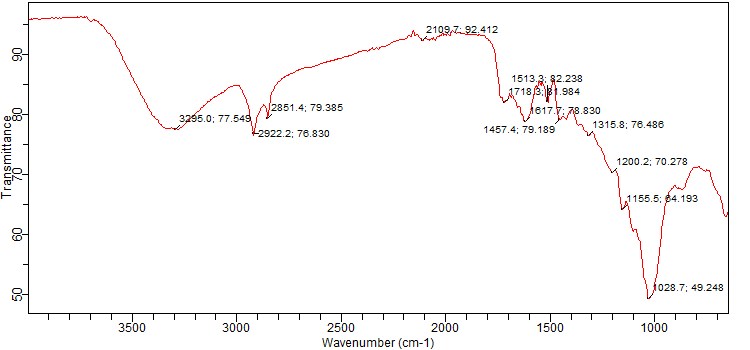


Acid pretreated cassava peels 300 microns Acid pretreated cassava peels 425 microns


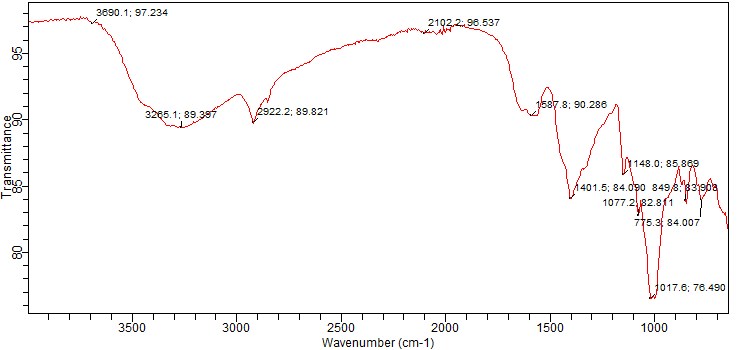

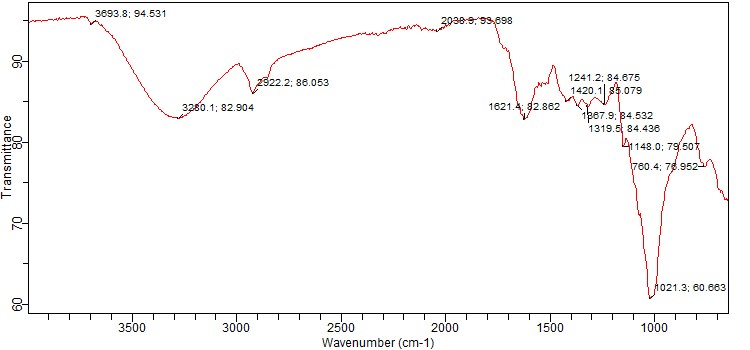


Alkali pretreated cassava peels 300 microns Hot water pretreated 300 microns


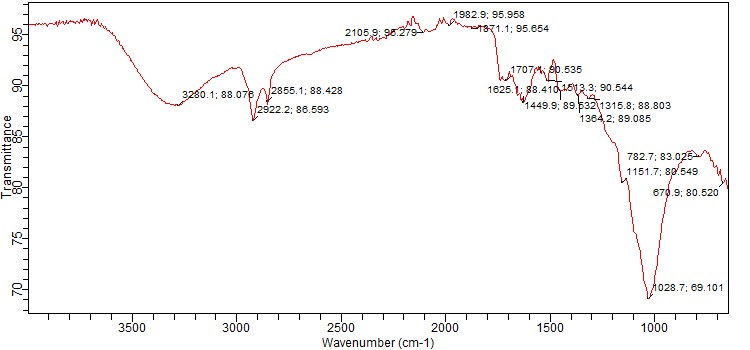


Hot water pretreated 425 microns

Figure S9 : FTIR of cassava peels biomass (Raw and pretreated)

Figure S10 : FTIR of corn cobs biomass (Raw and pretreated)


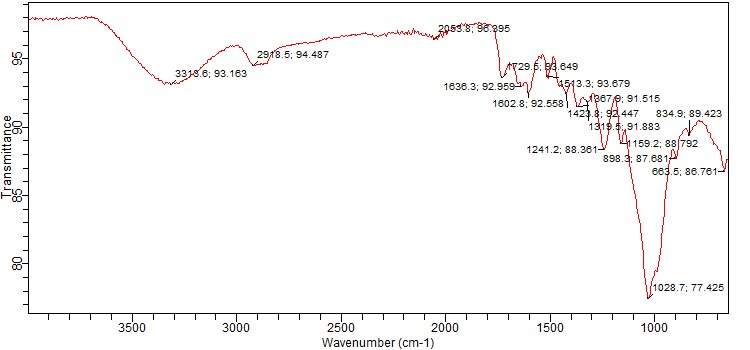

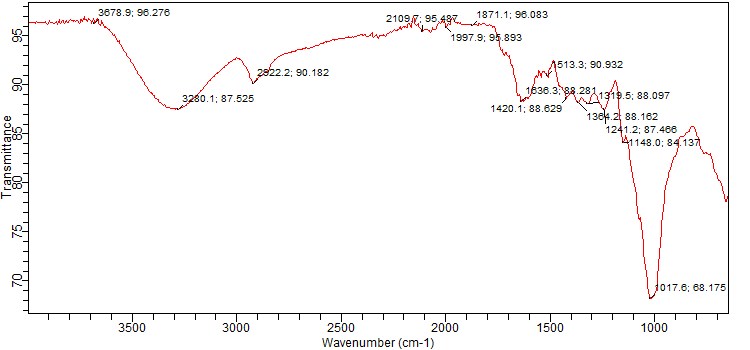


Corn cobs raw sample 425 microns Acid pretreated 300 microns


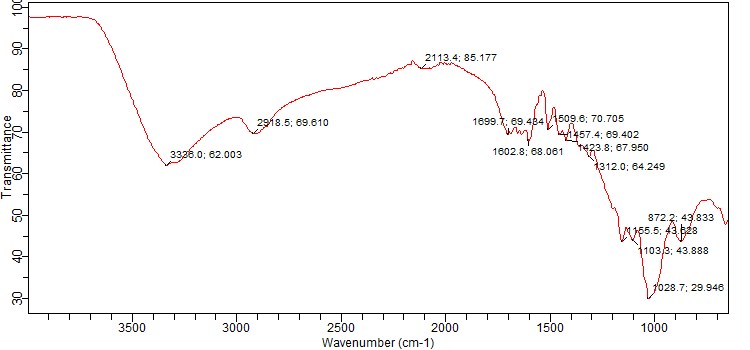

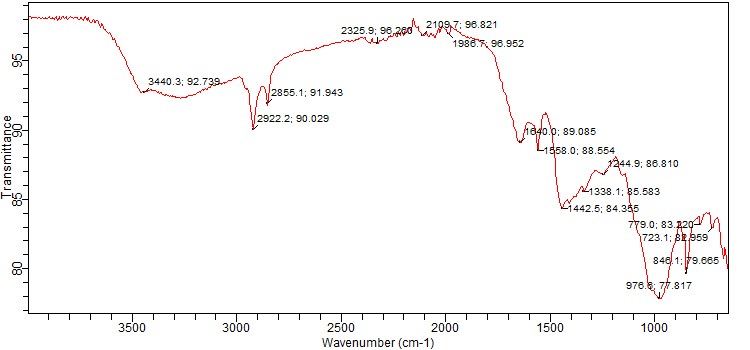


Acid pretreated 425 microns Alkali pretreated 300 microns


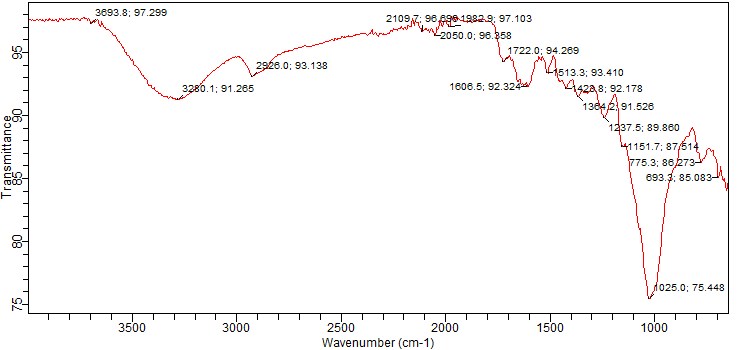

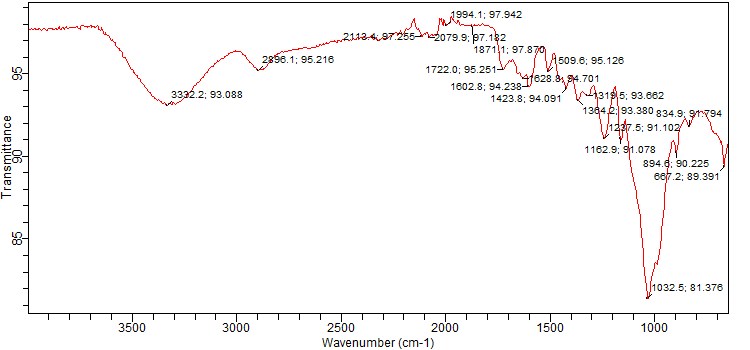


Hot water pretreated 300 microns Hot water pretreated 425 microns


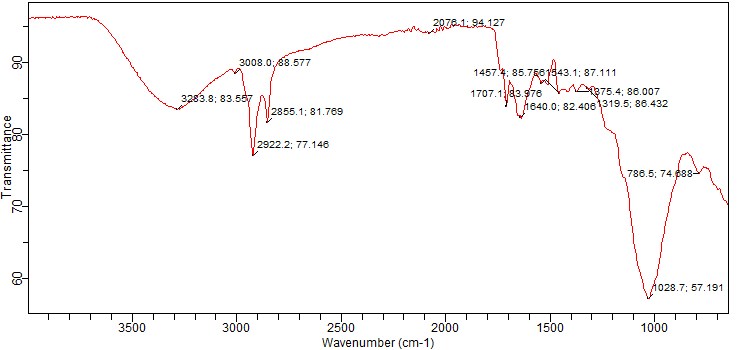

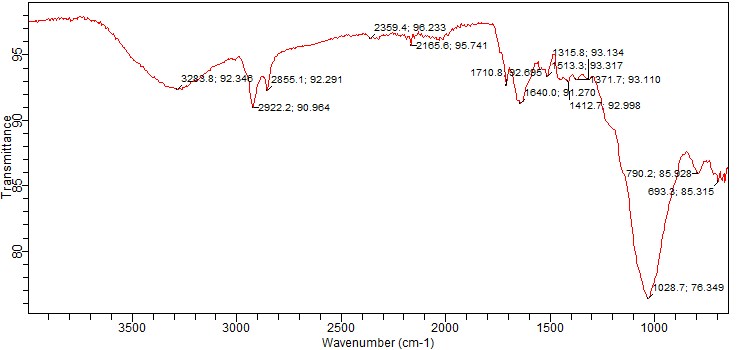


Raw rice husks biomass (300 microns) Raw rice husks biomass (425 microns)

#
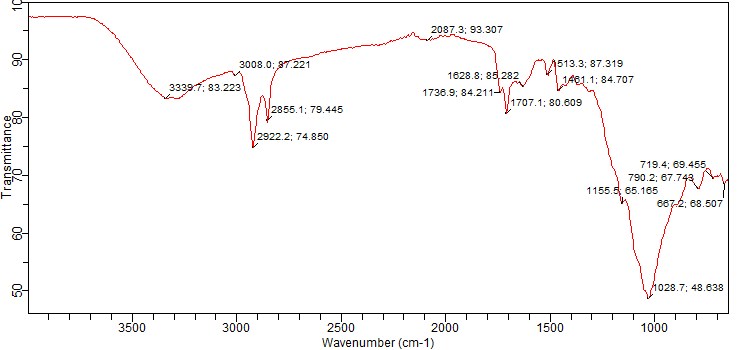

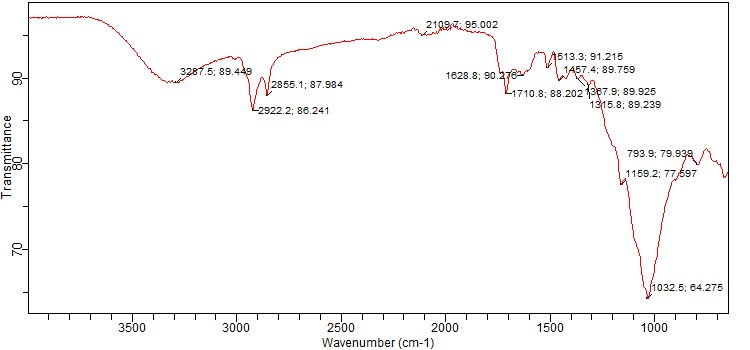


Acid pretreated 300 microns Acid pretreated 425 microns


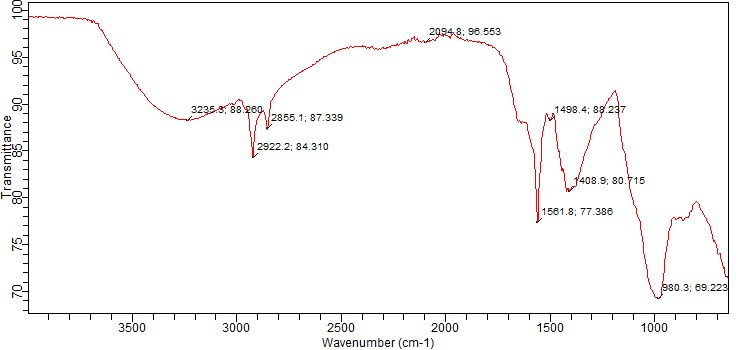

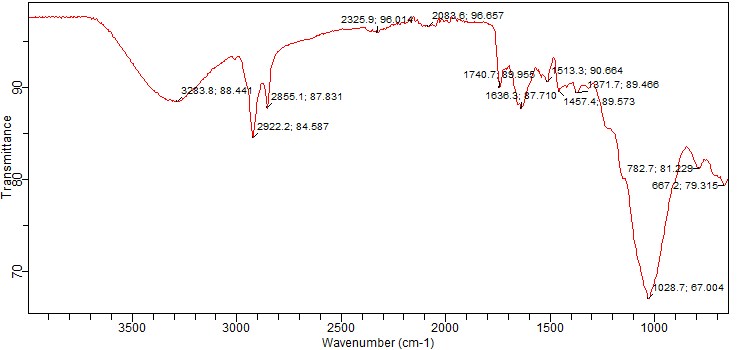


Alkali pretreated 300 microns Hot water pretreated 300 microns


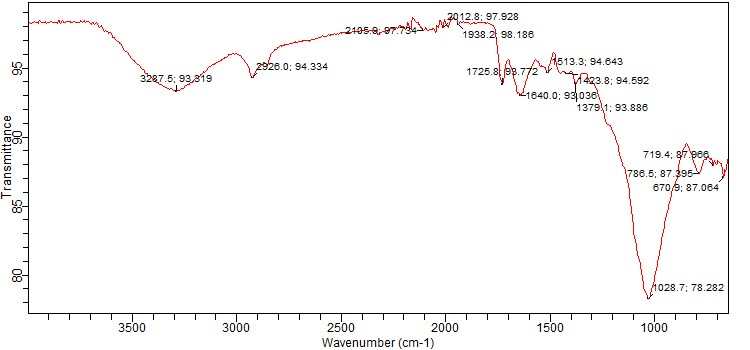


Hot water pretreated 425 microns

Figure S11 : FTIR of rice husks biomass


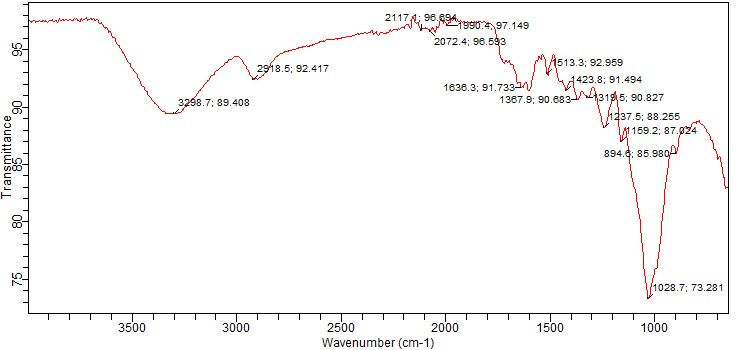

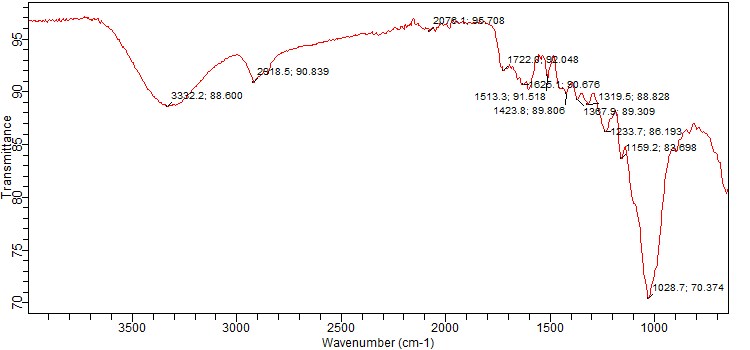


Raw sugar cane bagasse biomass 300 micron Raw sugar cane bagasse biomass 425 micron


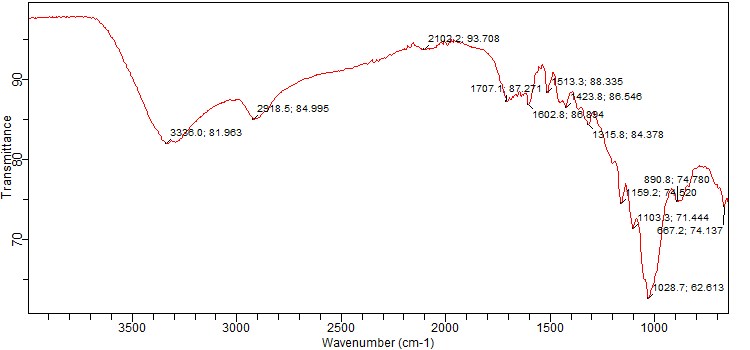

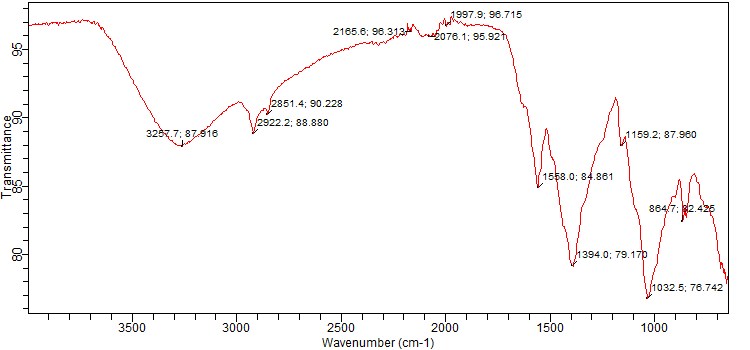


Acid pretreated 300 microns Alkali pretreated 300 microns


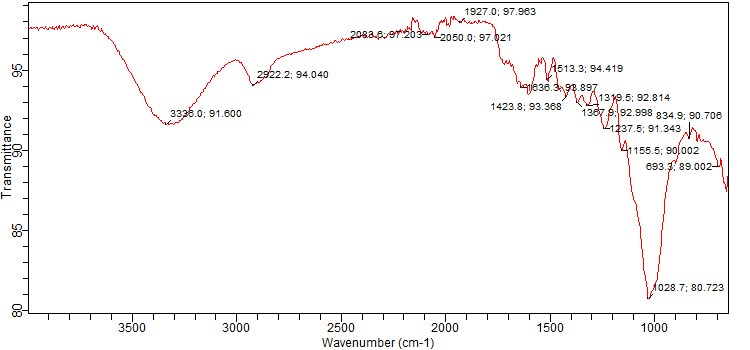


Hot water pretreated 300 microns

Figure S12 : FTIR of sugar cane bagasse biomass (Raw and pretreated)

Figure S13 : FTIR of yam peels biomass


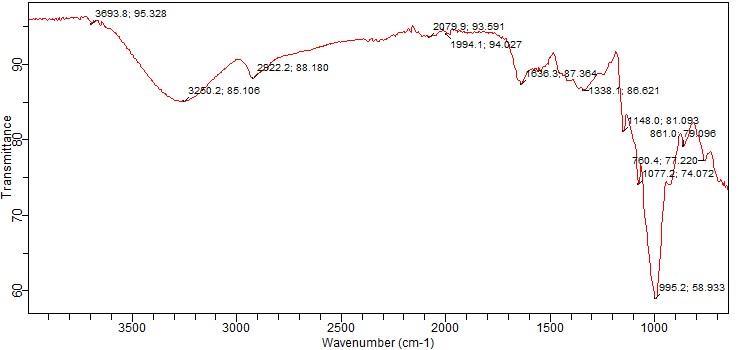

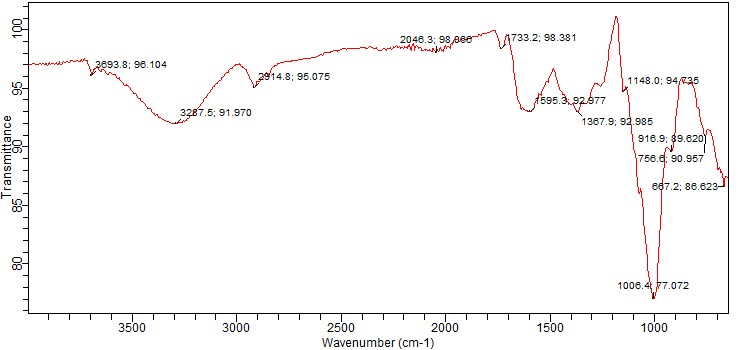


Raw yam peel biomass (300 micron) Raw yam peel biomass (425 micron)


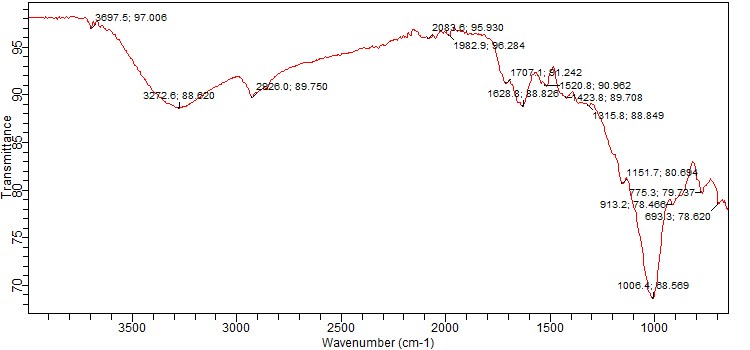

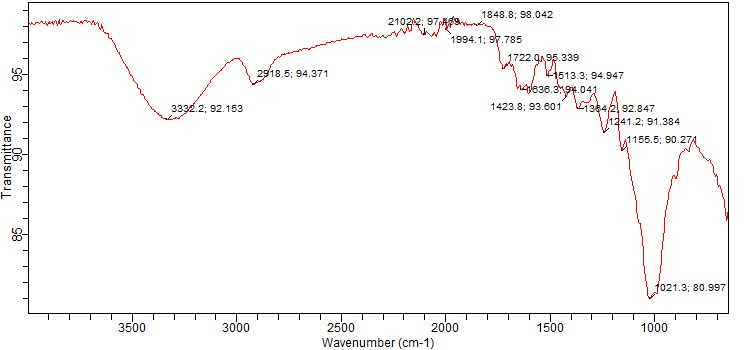


Acid pretreated 300 microns Alkali pretreated 300 microns


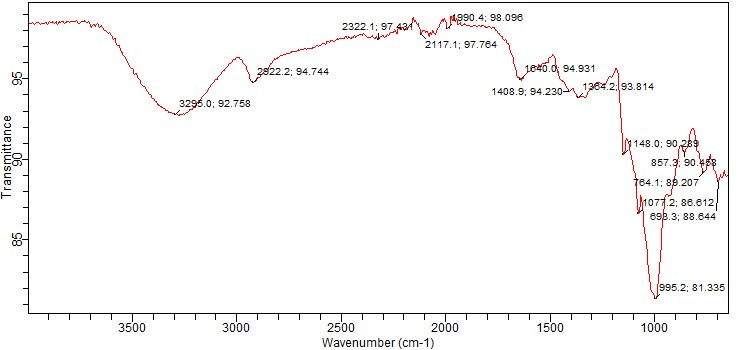


Hot water pretreated 300 microns


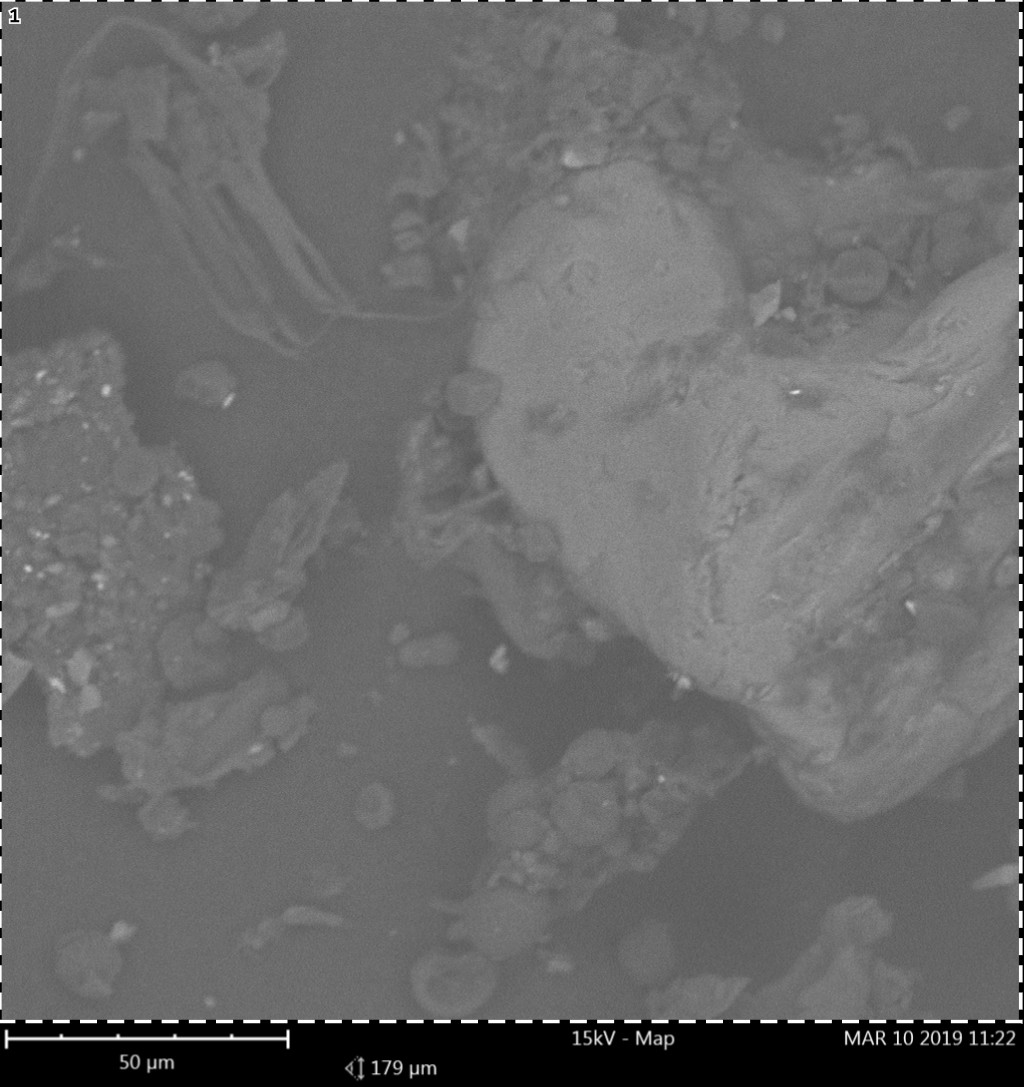

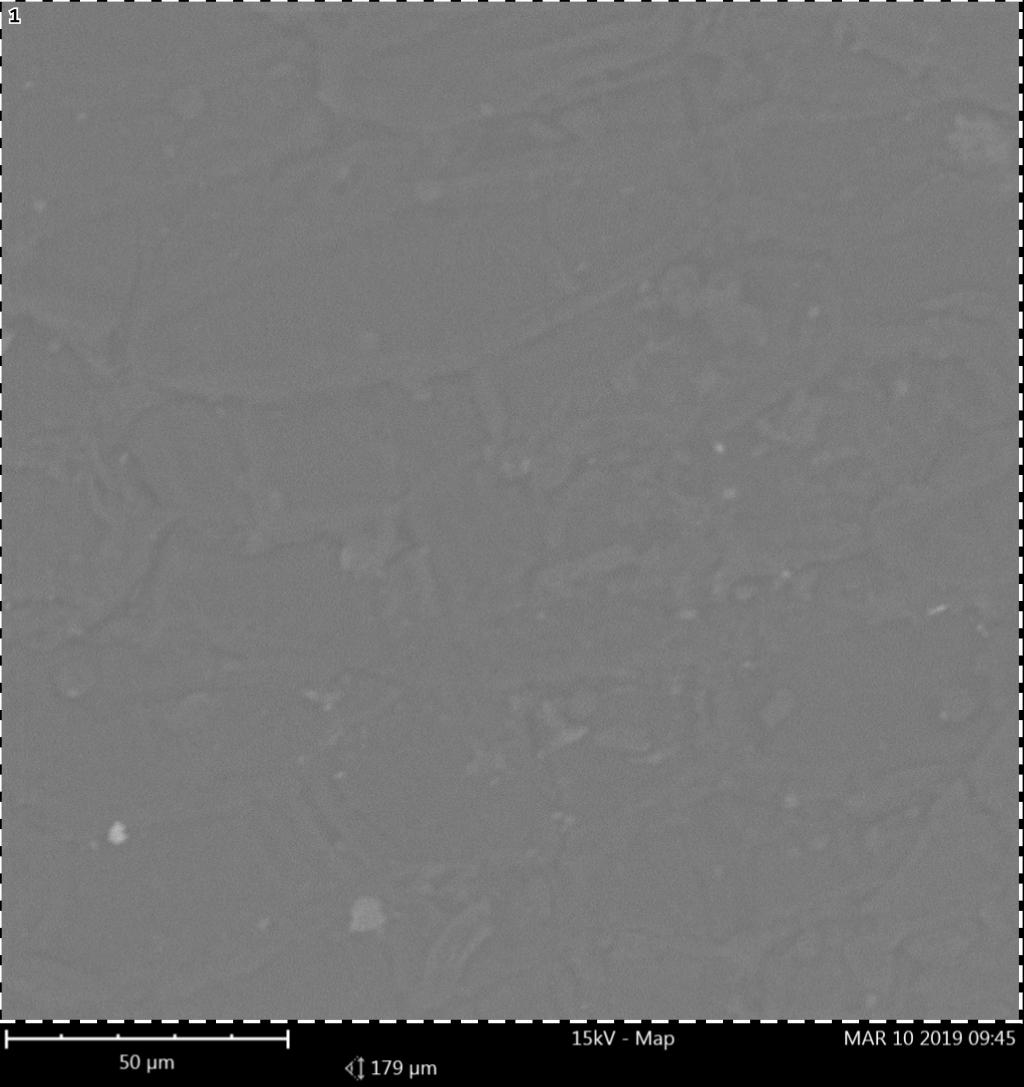


CASSAVA PEELS 300 MICRONS RAW BIOMASS CASSAVA PEELS 425 MICRONS RAW BIOMASS


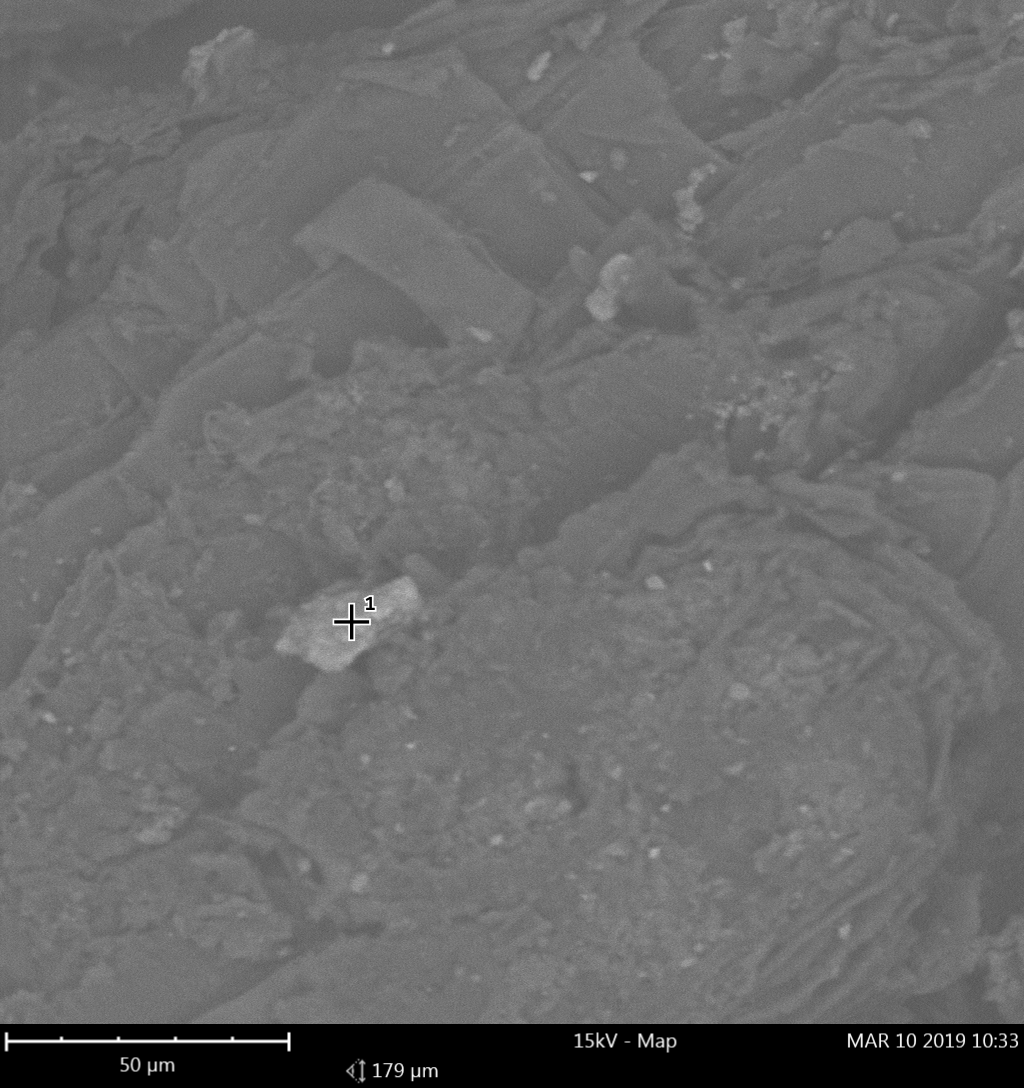

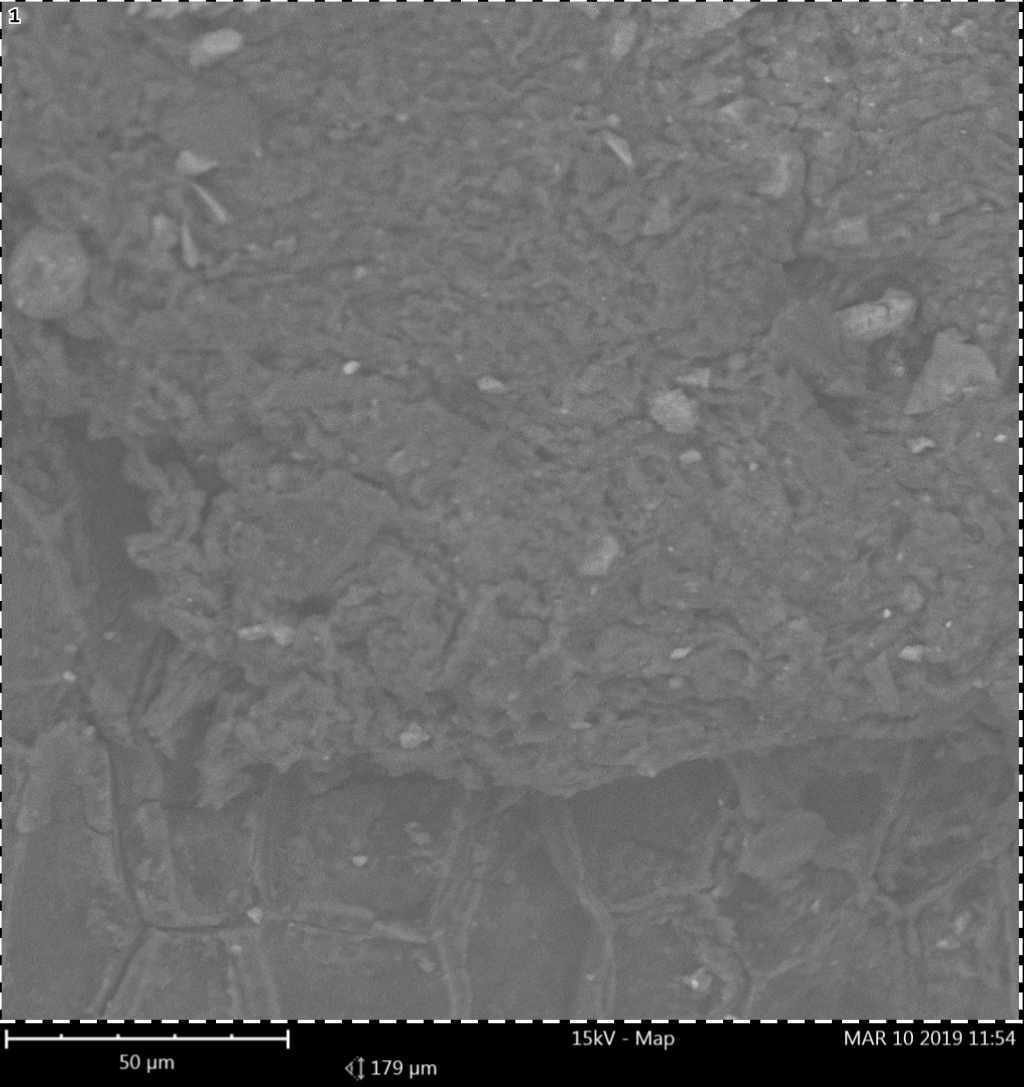


ACID PRETREATED CASSAVA PEELS 300 MICRONS ACID PRETREATED CASSAVA PEELS 425 MICRONS


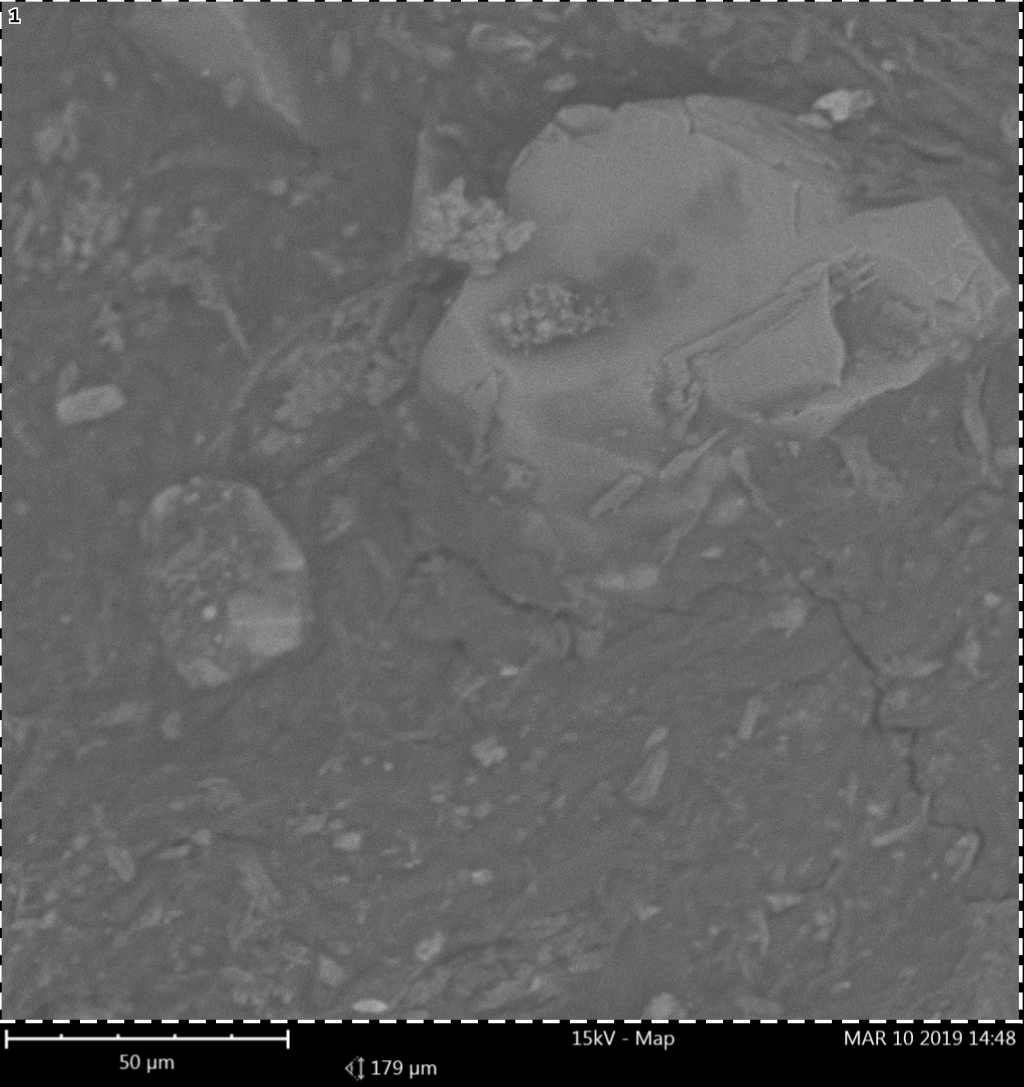

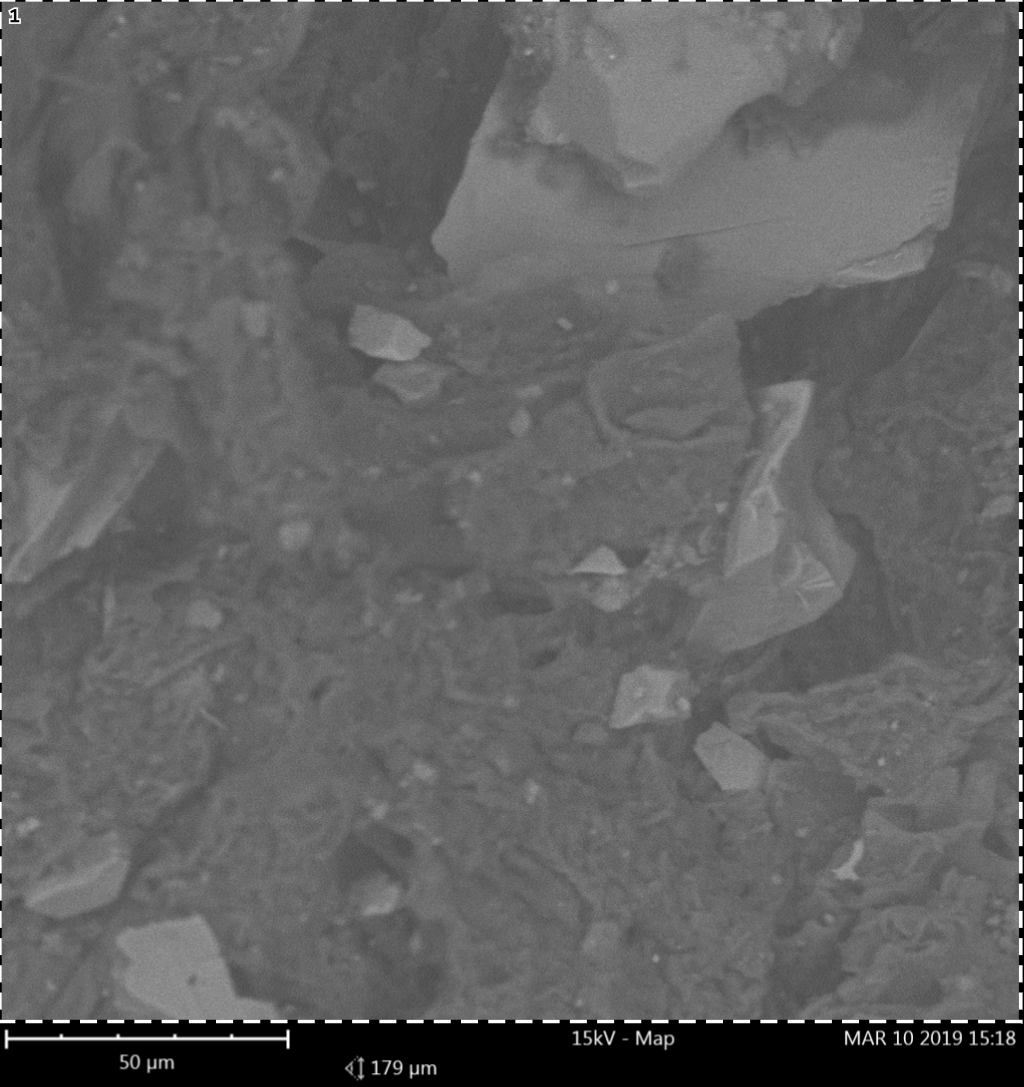


ALKALI PRETREATED CASSAVA PEELS 300 MICRONS HOT WATER PRETREATED CASSAVA PEELS 300 MICRONS

Figure S14 : SEM of cassava peels biomass (Raw and pretreated)

Figure S15 : SEM of Corn cobs biomass (Raw and pretreated)


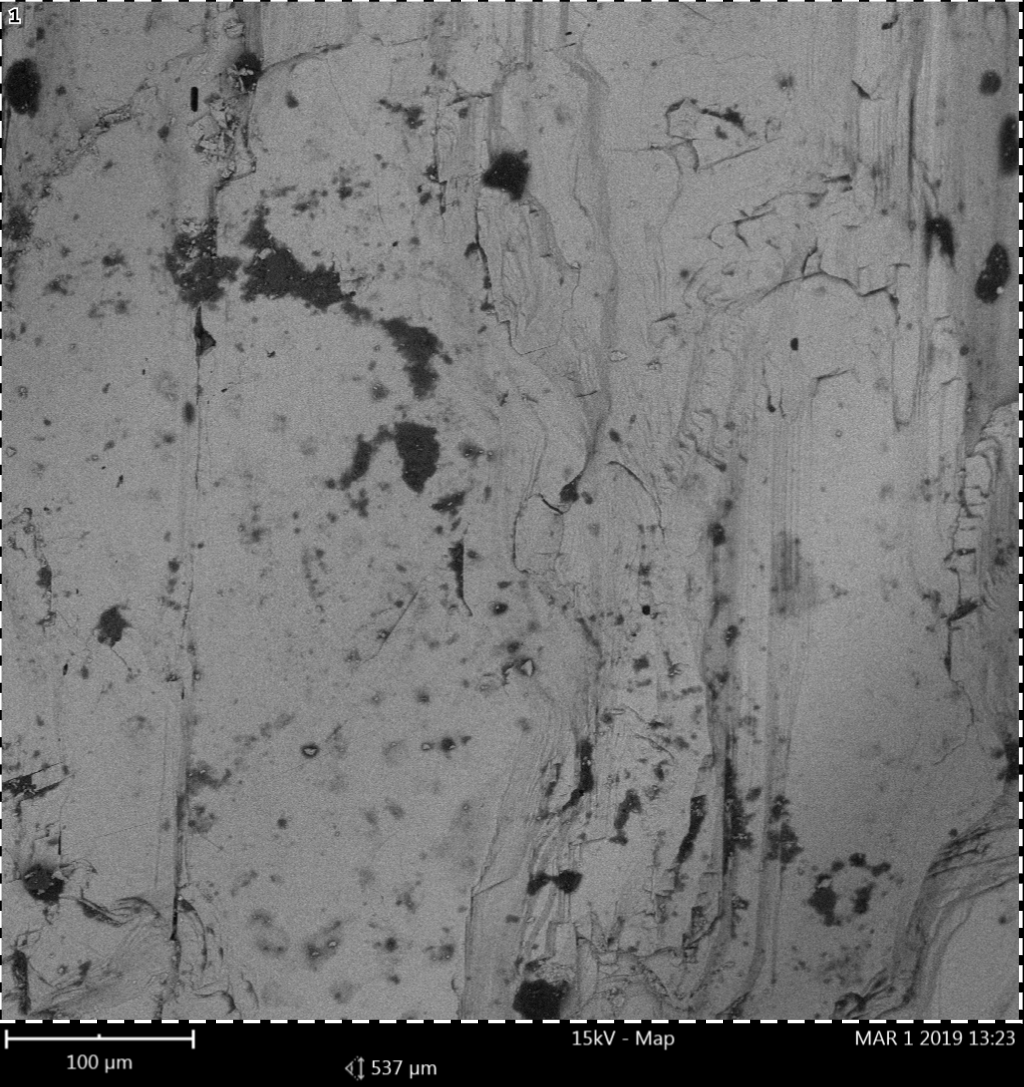

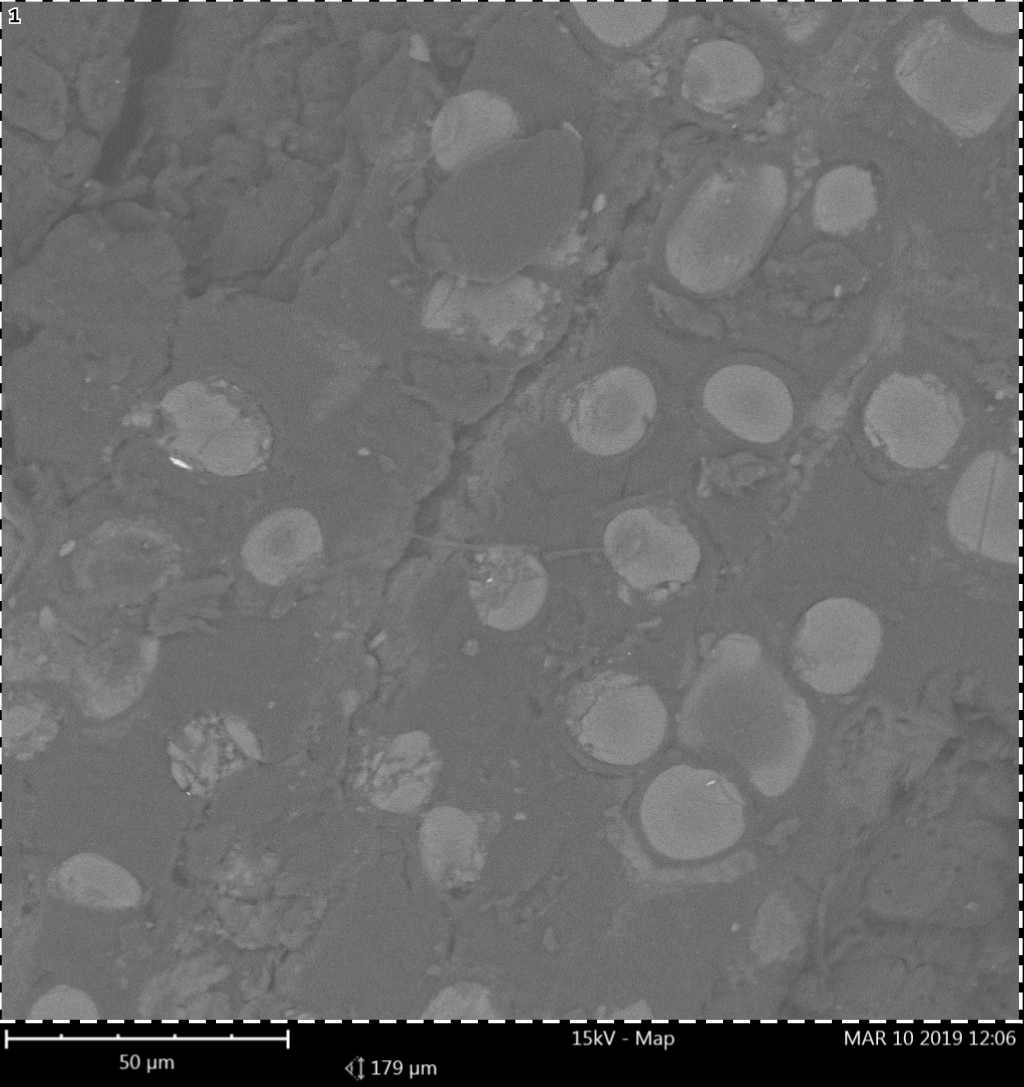


Raw corn cobs biomass (300 microns) Raw corn cobs biomass (425 microns)


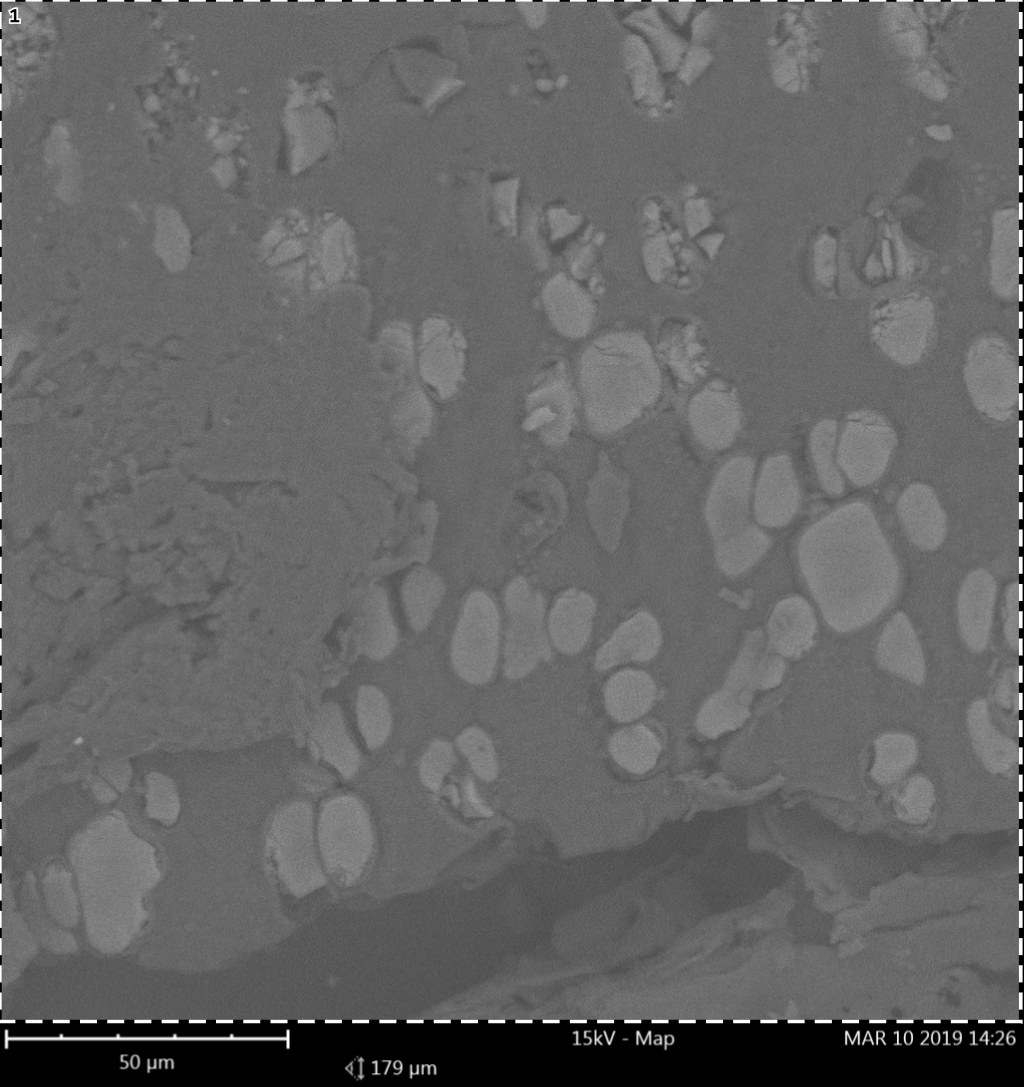

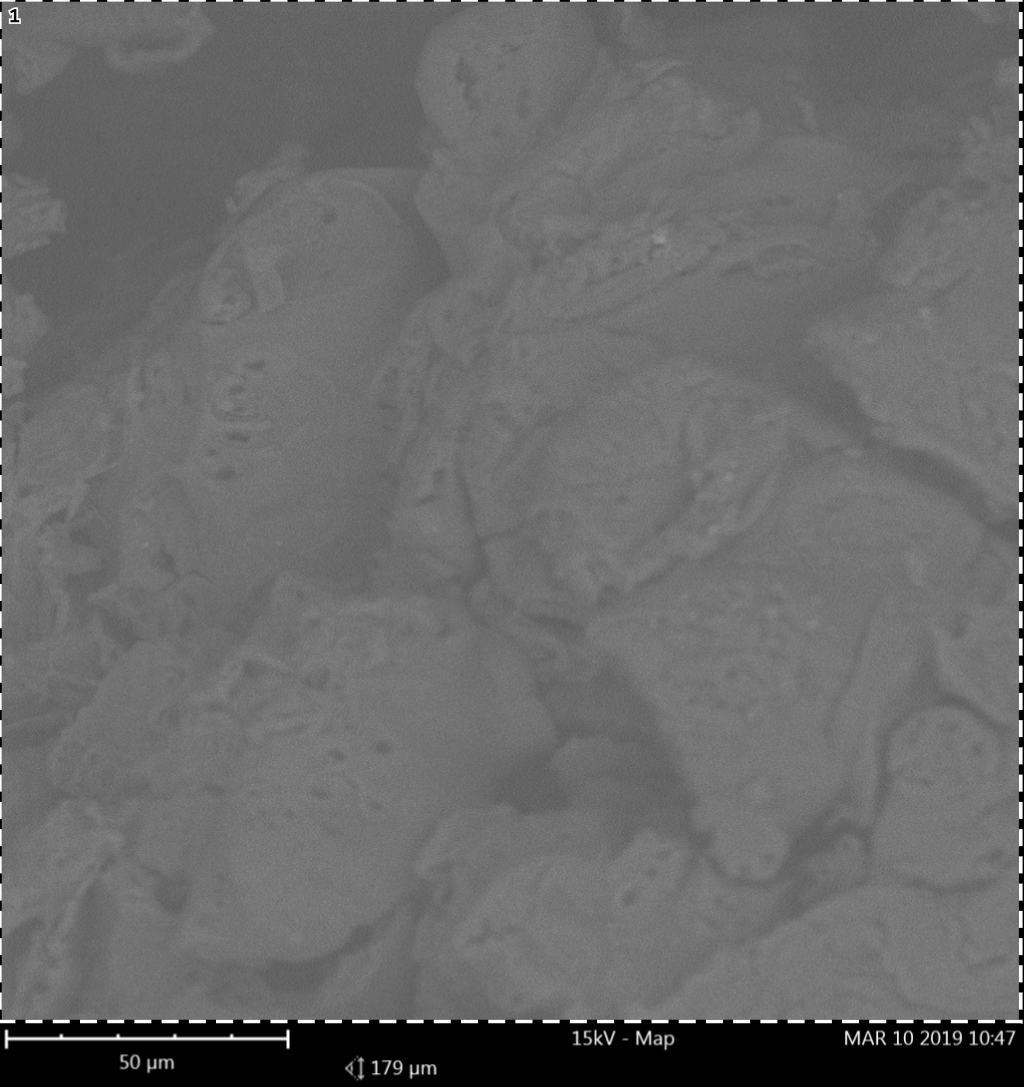


Acid Pretreated corn cobs biomass (300 microns) Acid Pretreated corn cobs biomass (425 microns)


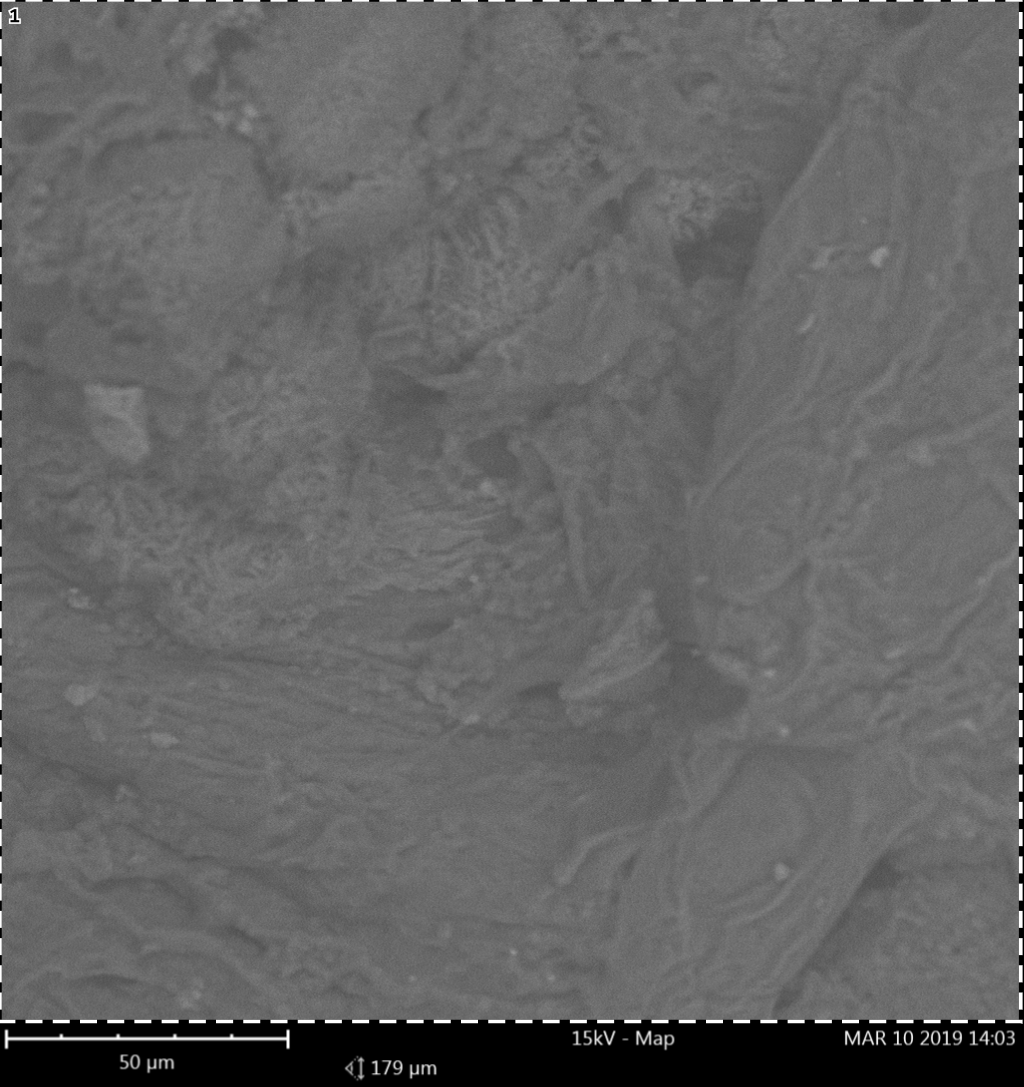

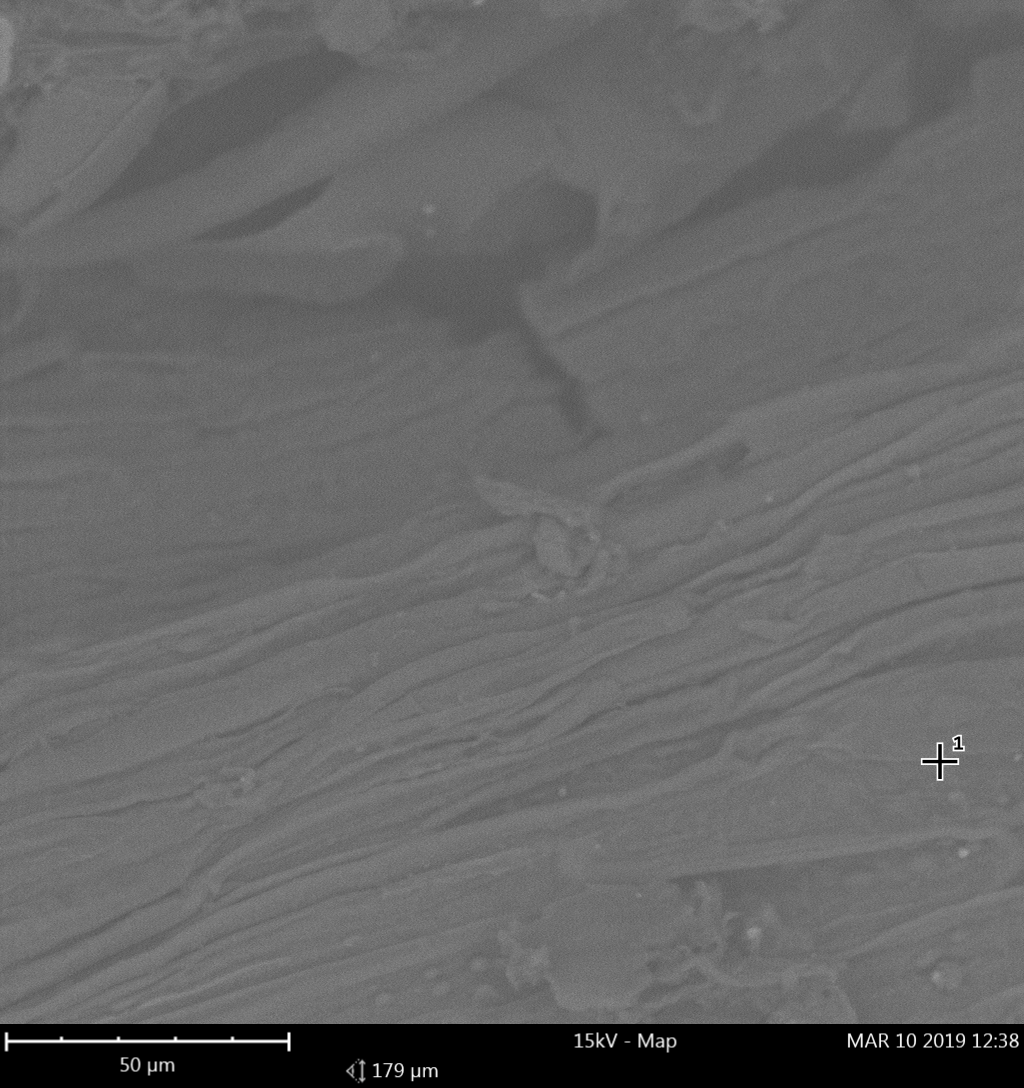


Alkali Pretreated corn cobs biomass (300 microns) Hot water Pretreated corn cobs biomass (300 microns)

Figure S16: SEM of raw and pretreated Rice husks biomass


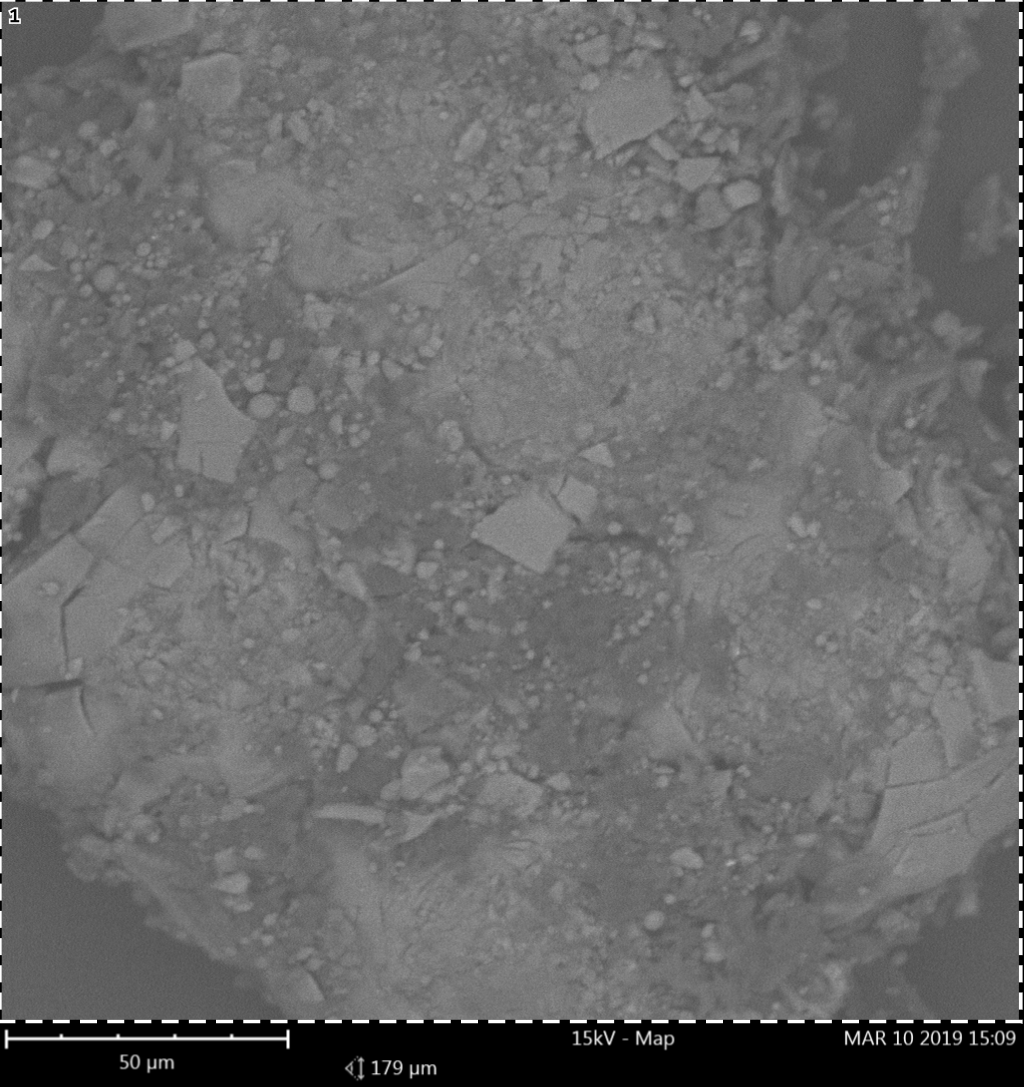

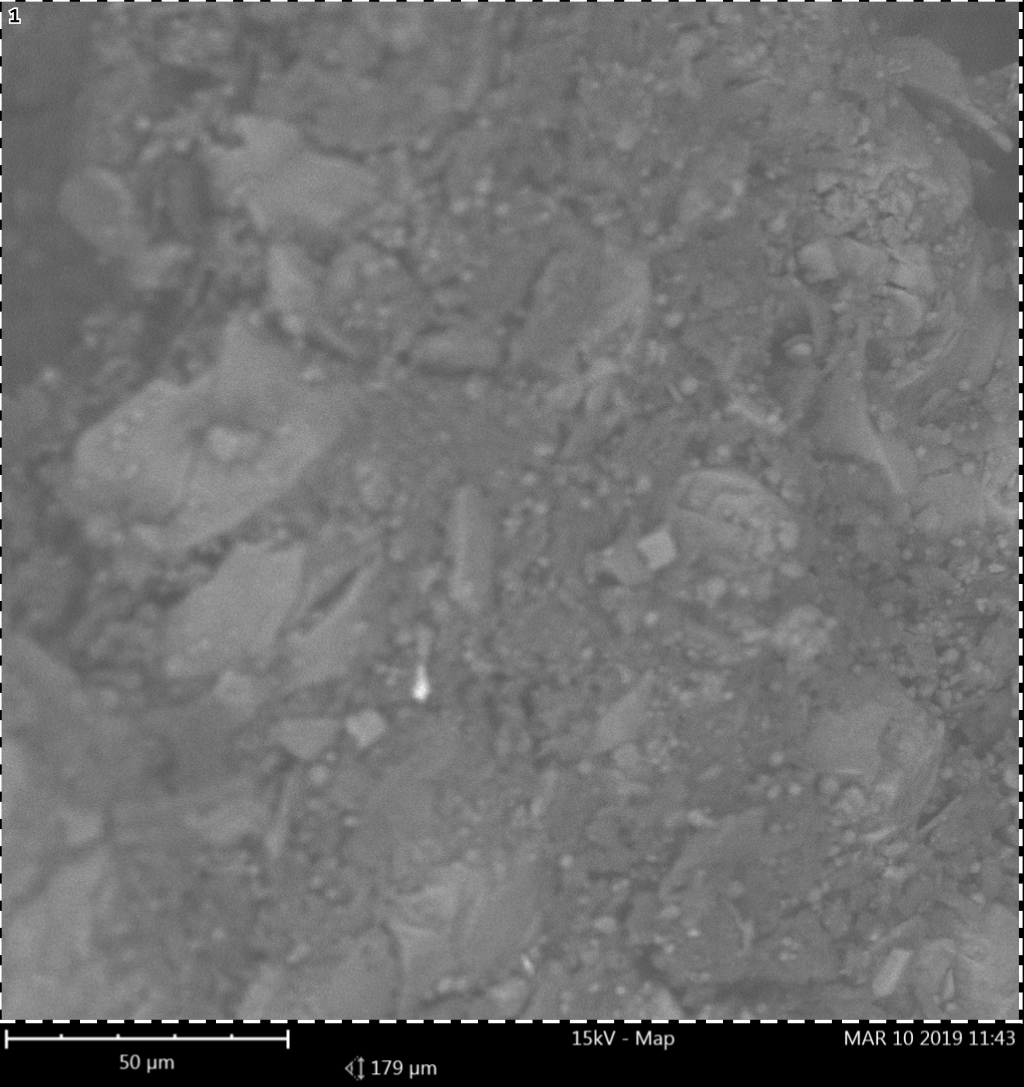


Raw rice husks biomass 300 microns Raw rice husks biomass 425 microns


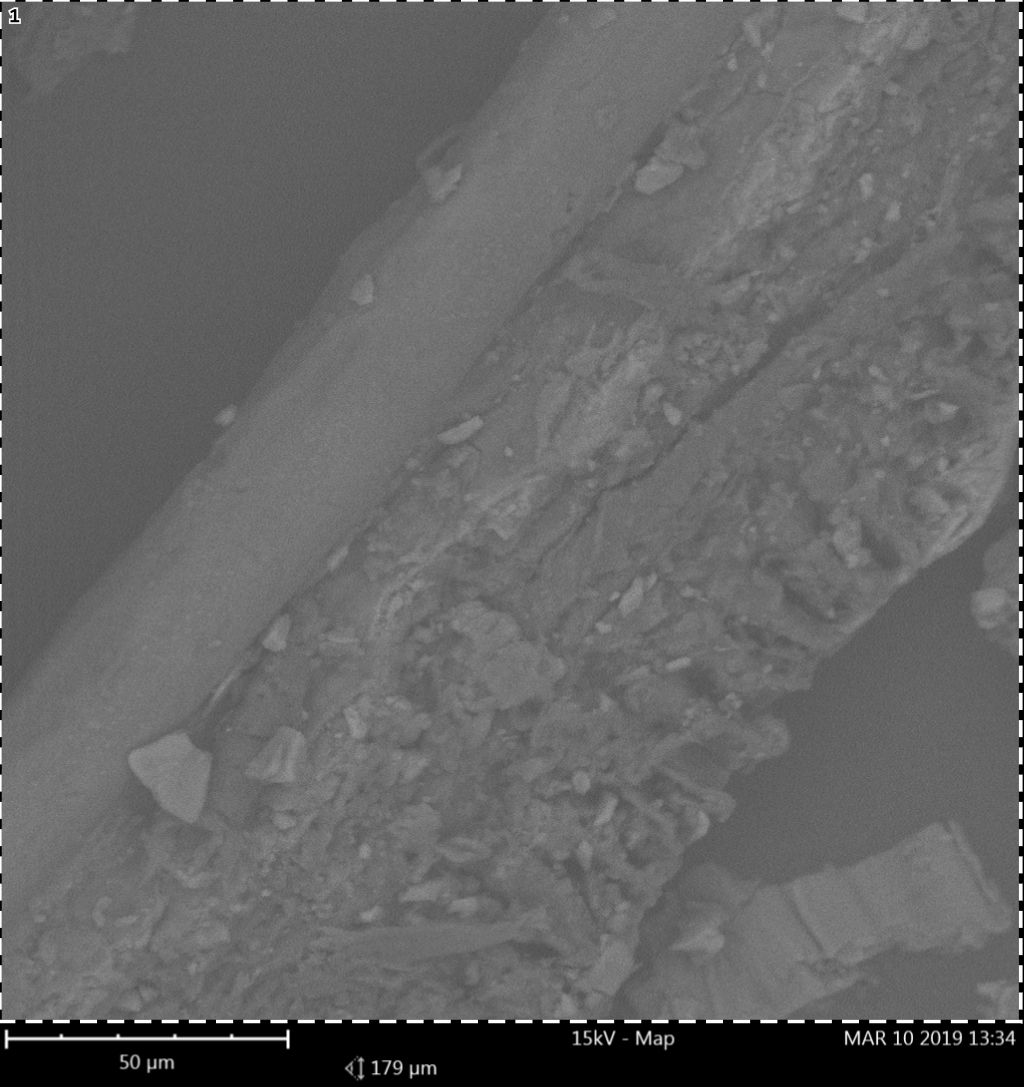

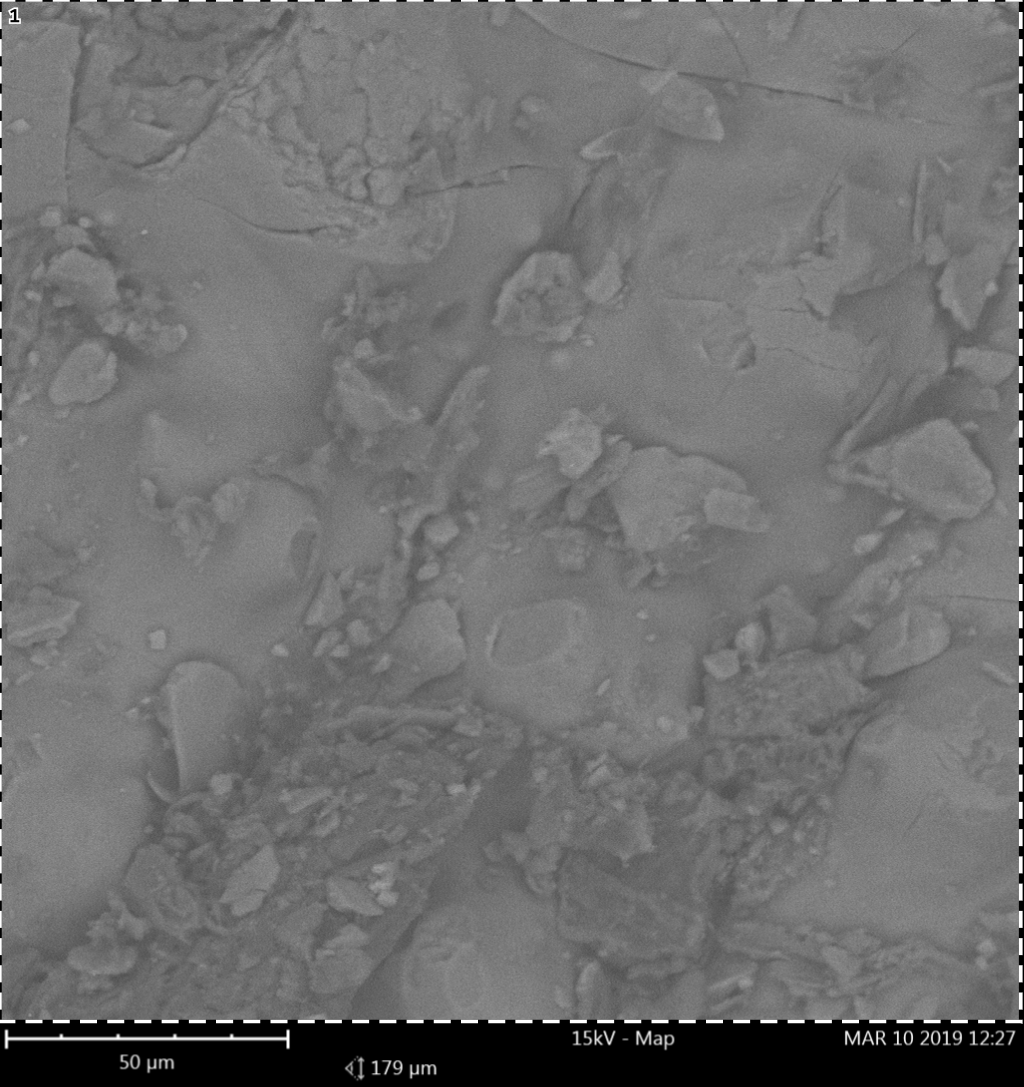


Acid pretreated 300 microns rice husks Acid pretreated 425 microns rice husks


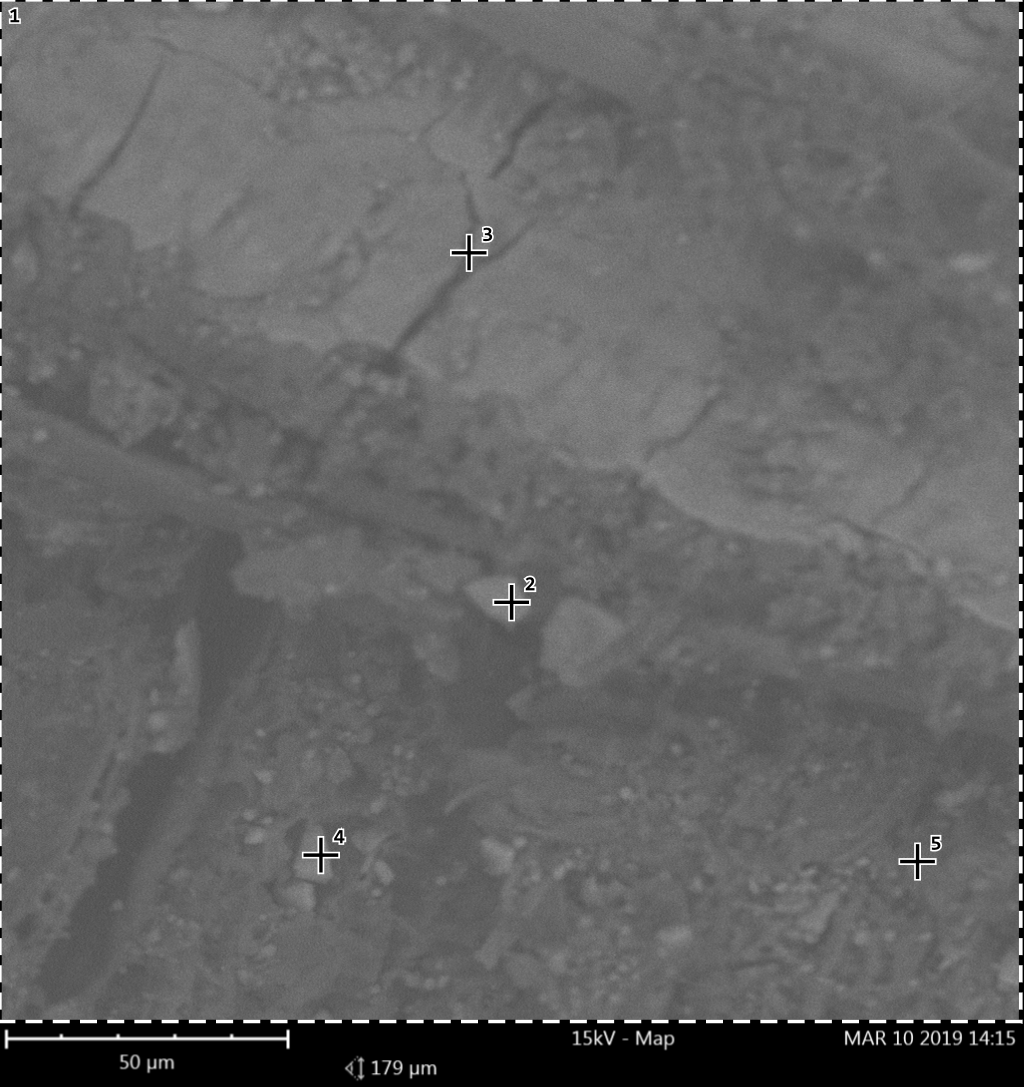

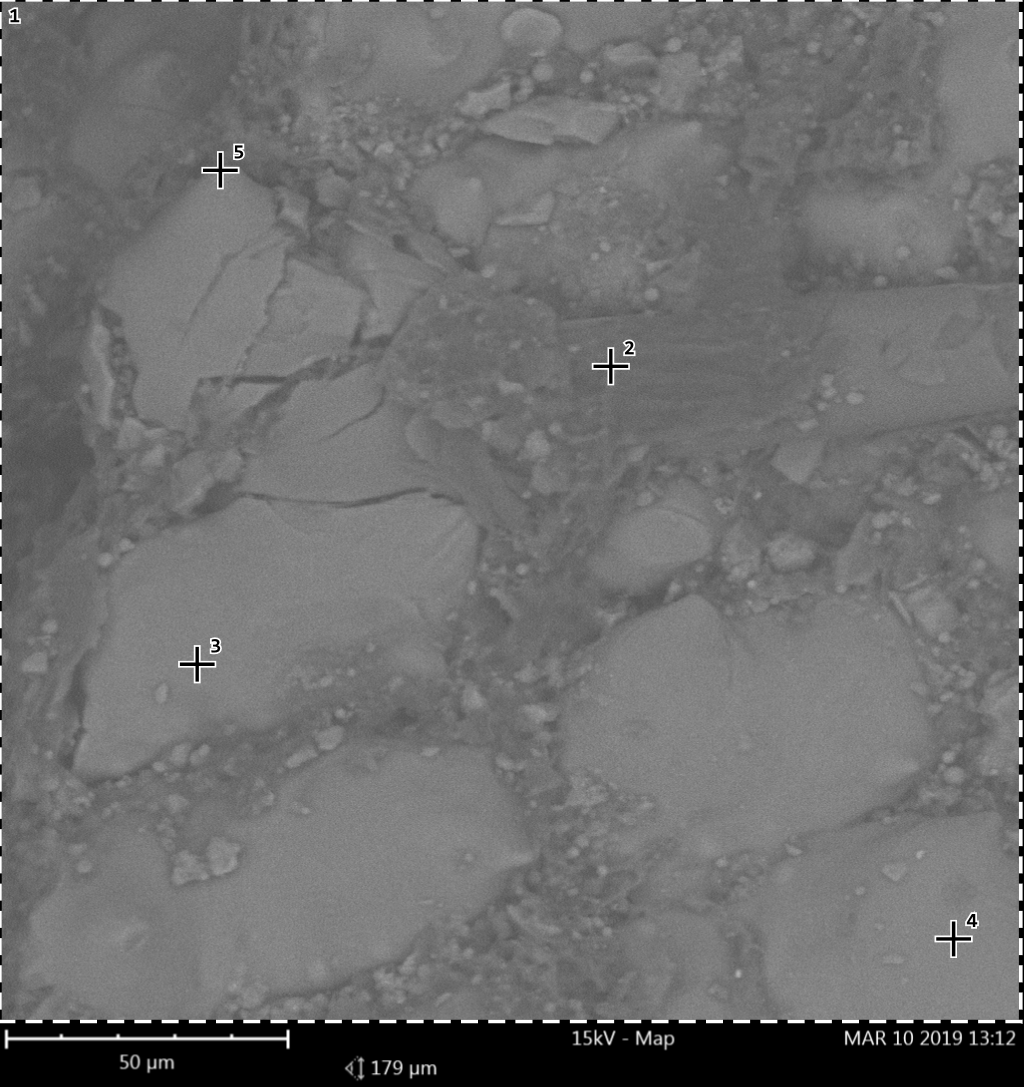


Hot water pretreated 300 microns rice husks Hot water pretreated 425 microns rice husks

Figure S17: SEM of raw and pretreated sugar cane bagasse biomass


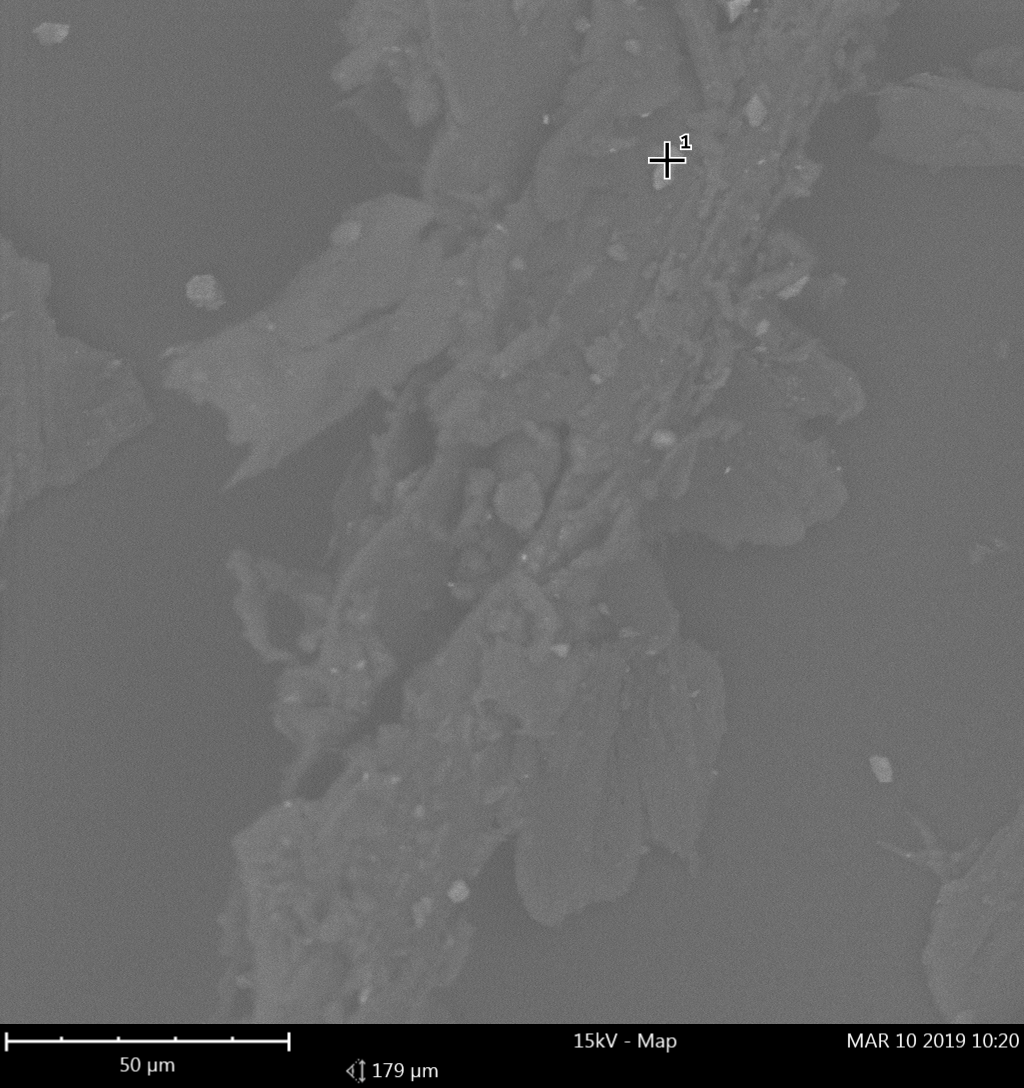

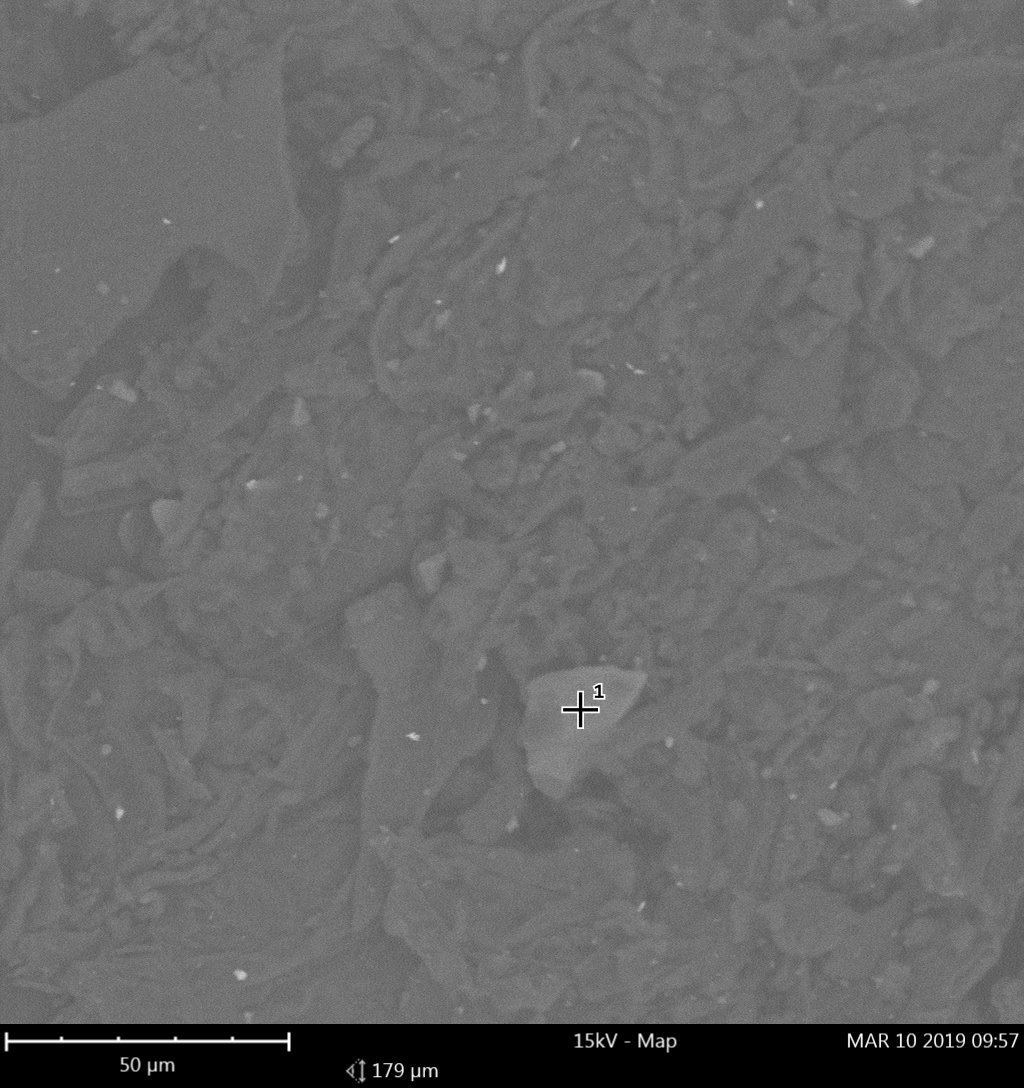


Raw sugar cane bagasse 300 microns Raw sugar cane bagasse 425 microns


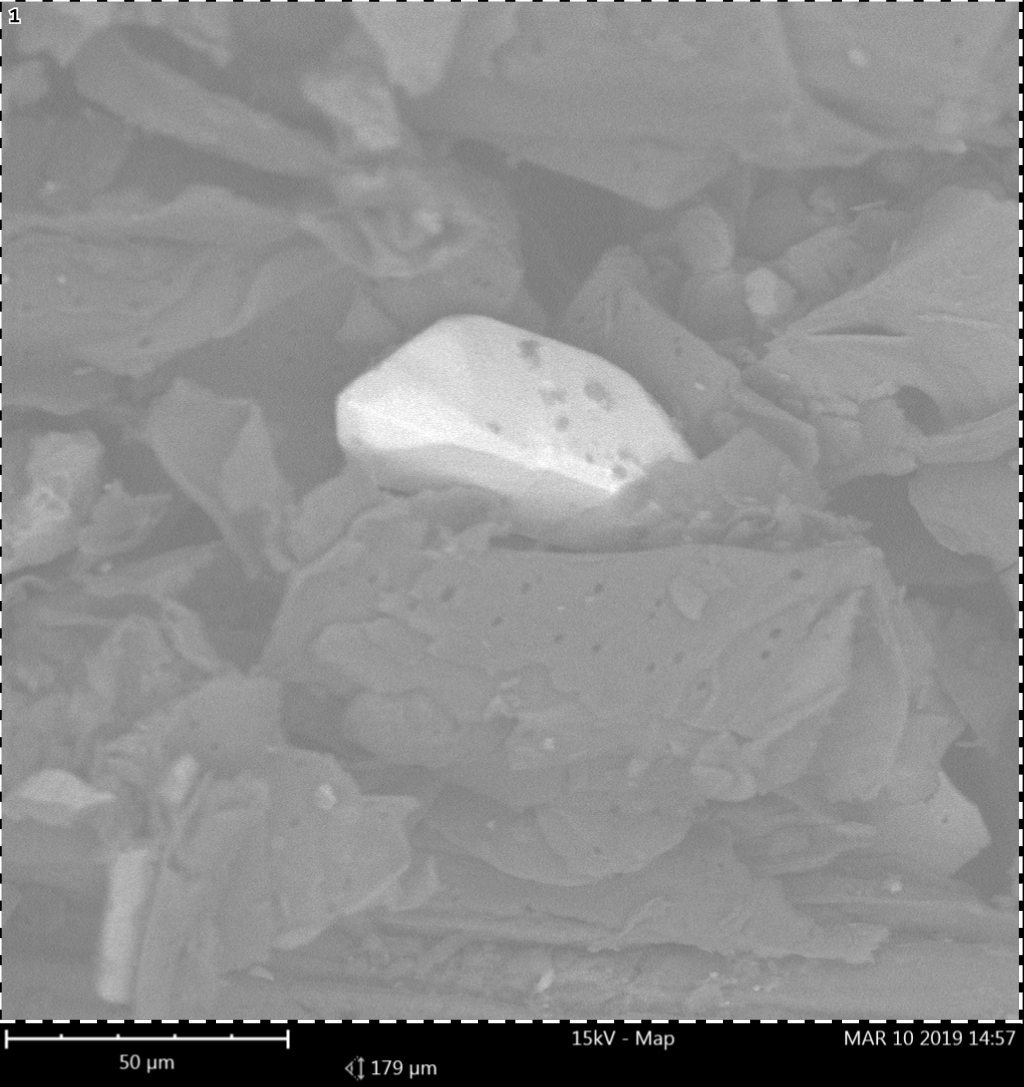

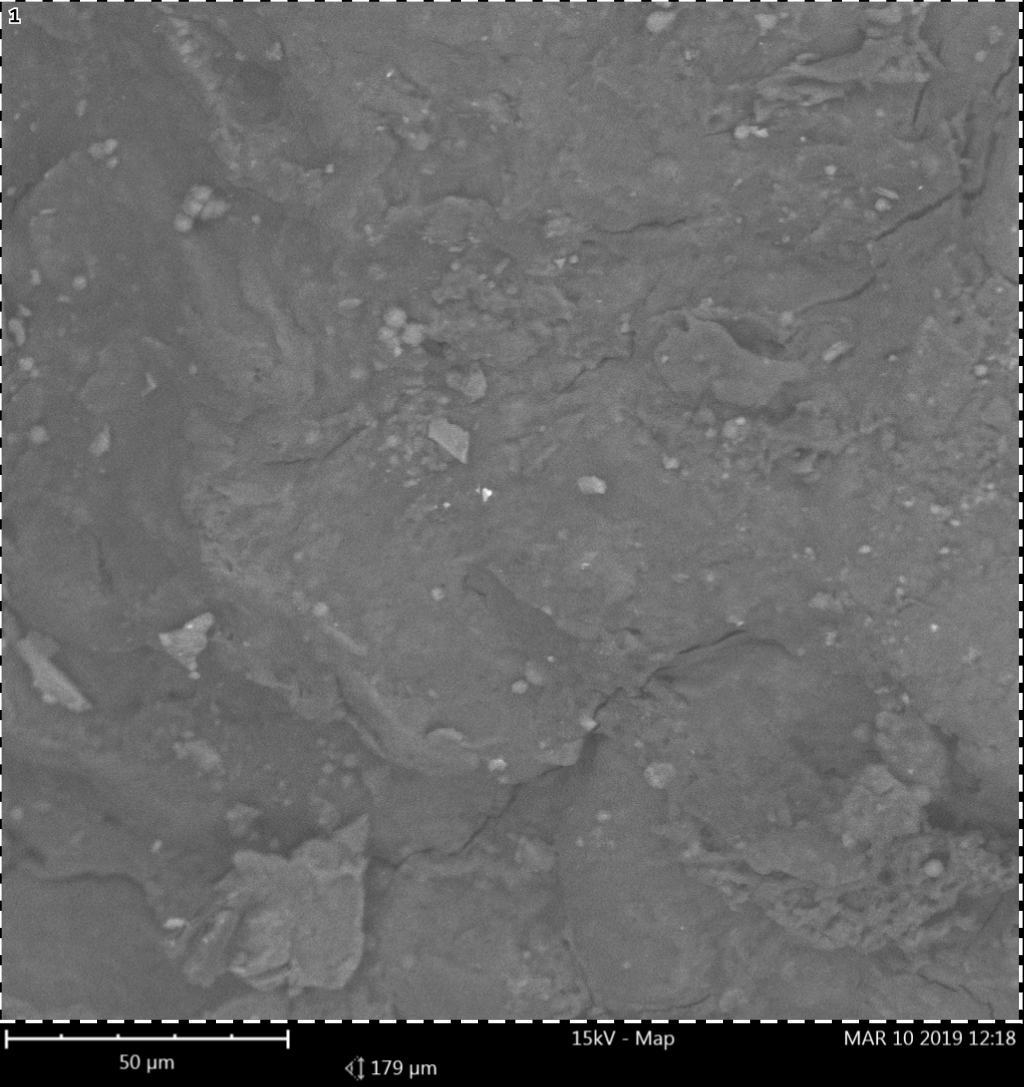


Acid Pretreated 300 microns Alkali Pretreated 300 microns


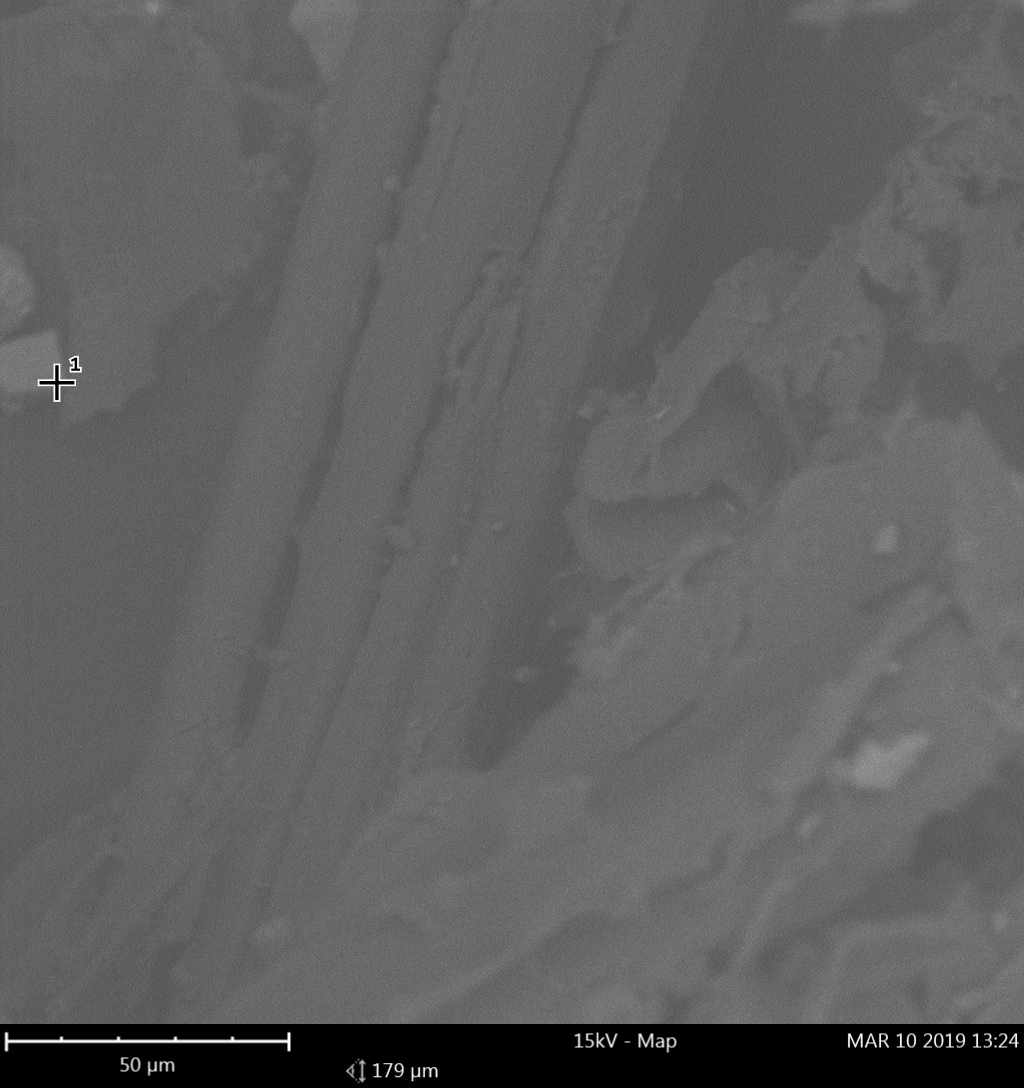


Hot water pretreated 300 microns

Figure S18 : SEM of raw and pretreated yam peels biomass


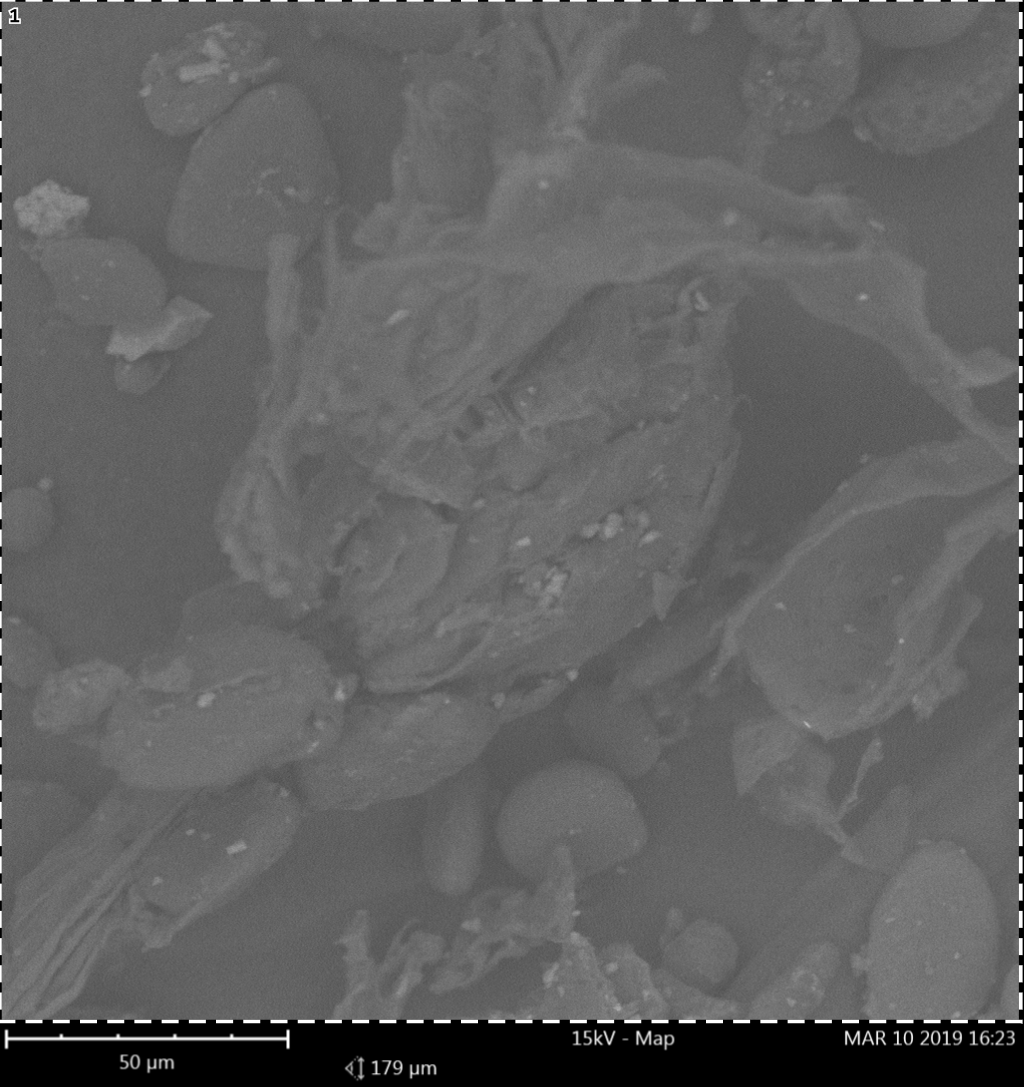

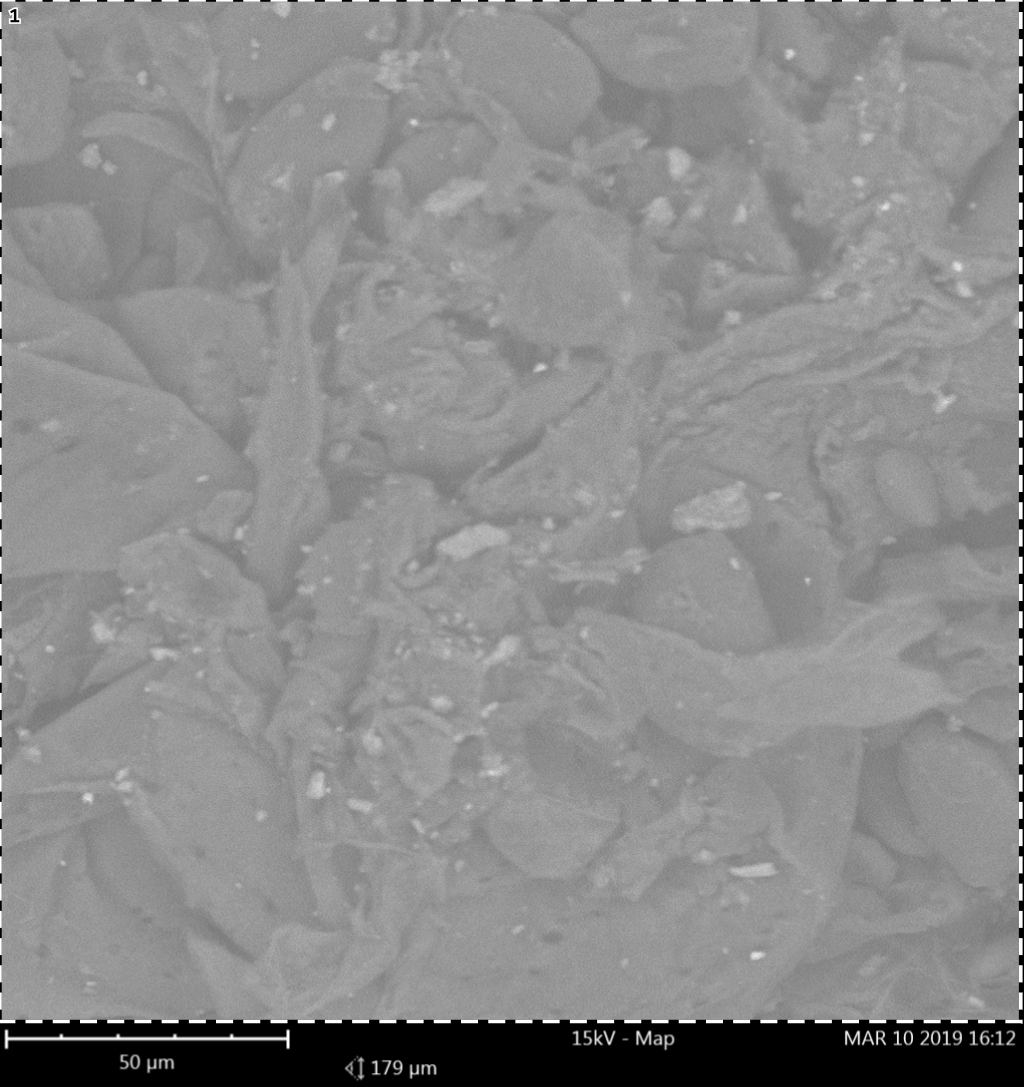


Raw yam peels biomass 300 microns Raw yam peels biomass 425 microns


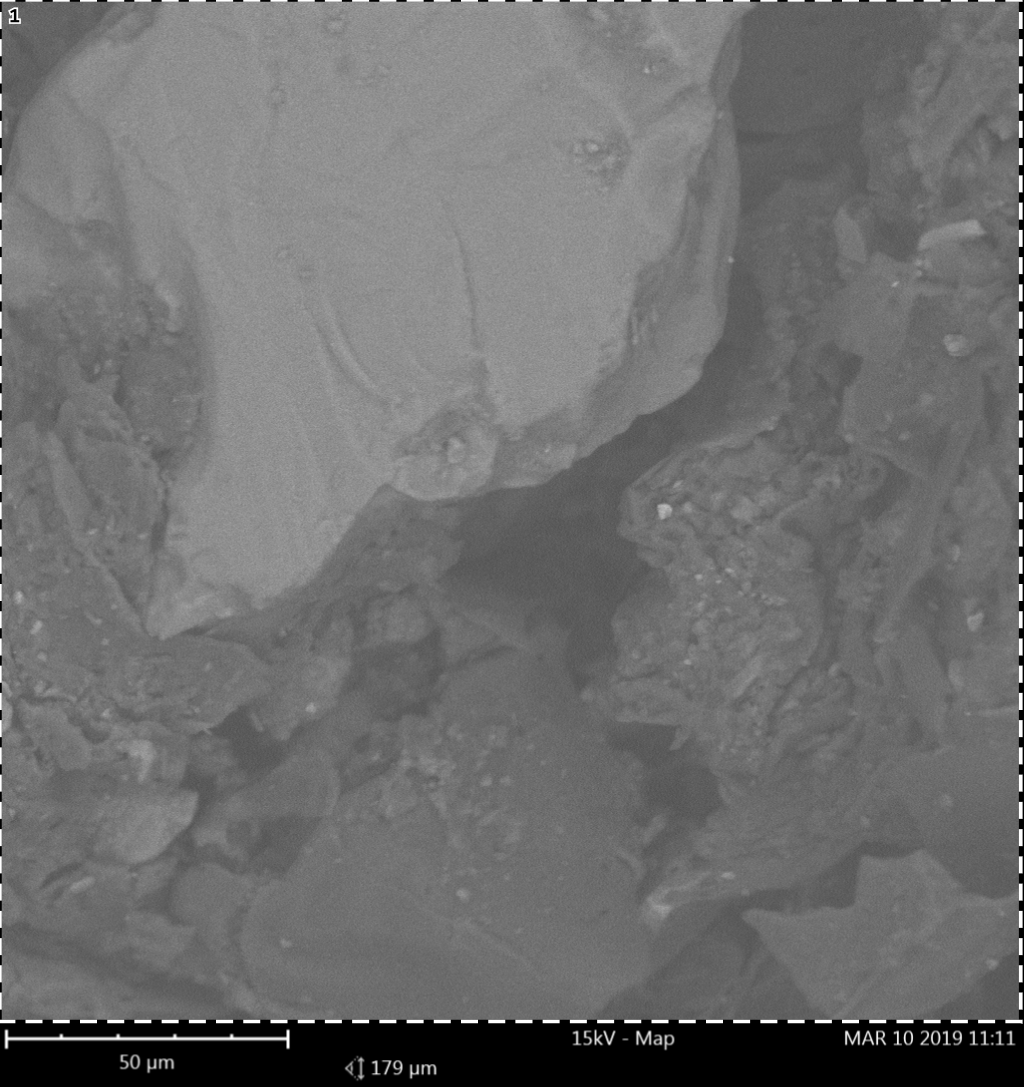

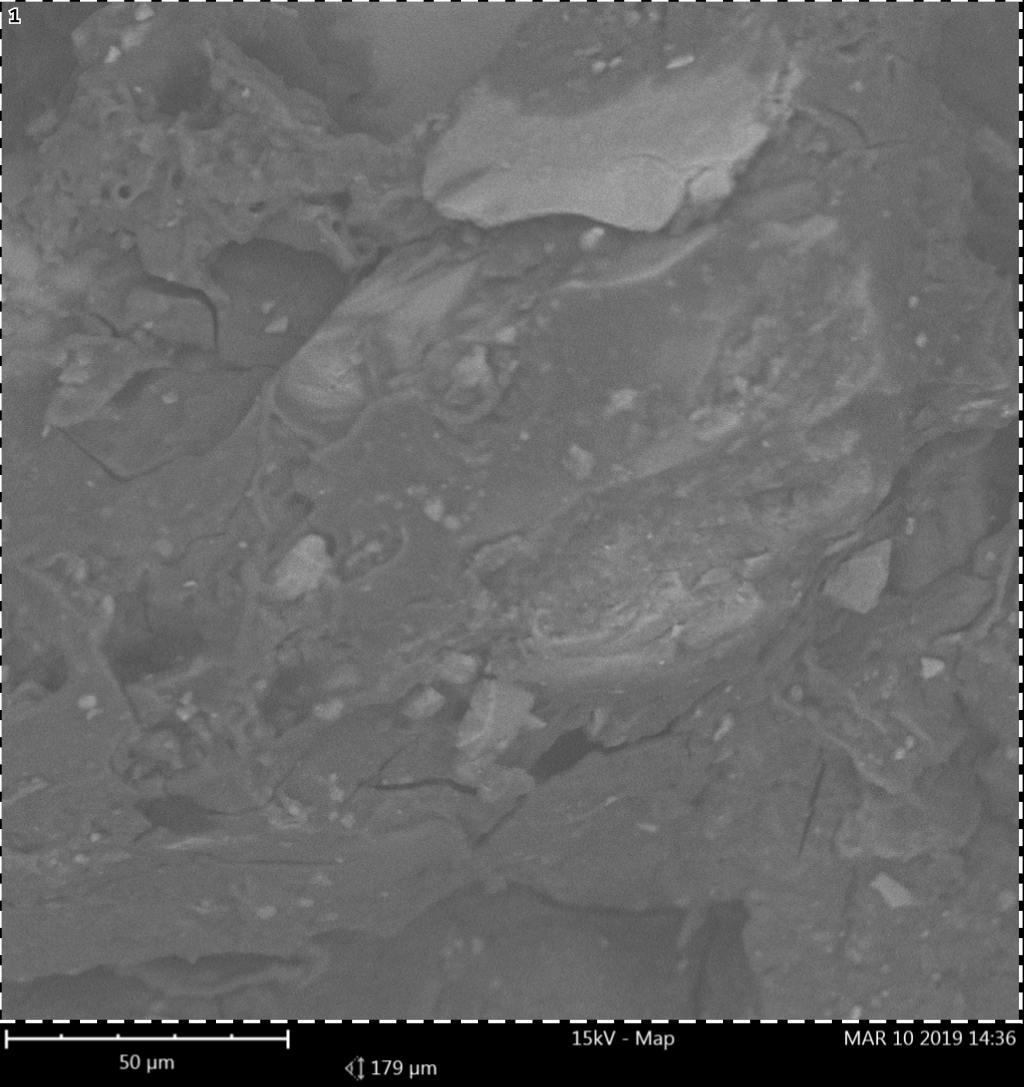


Acid pretreated 300 microns Hot water pretreated 425 microns


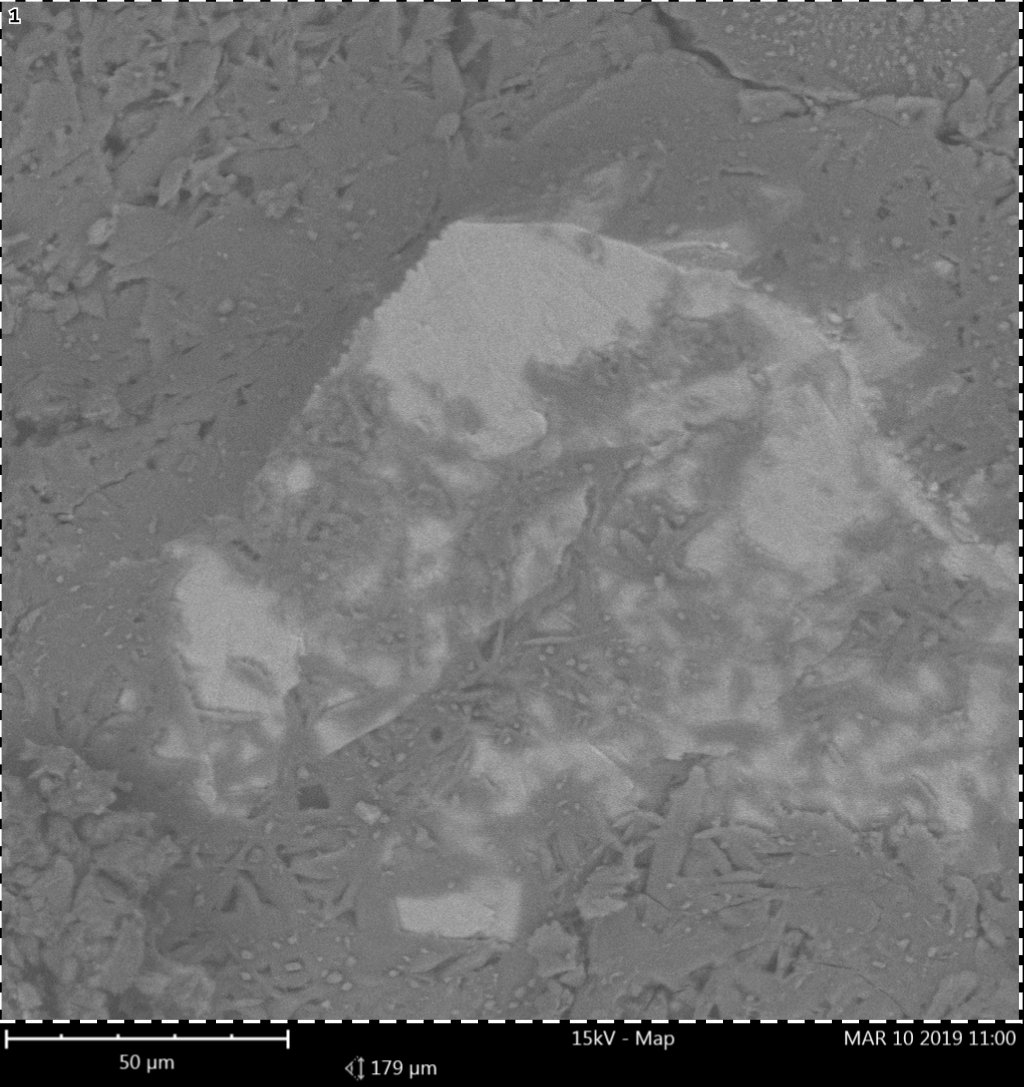

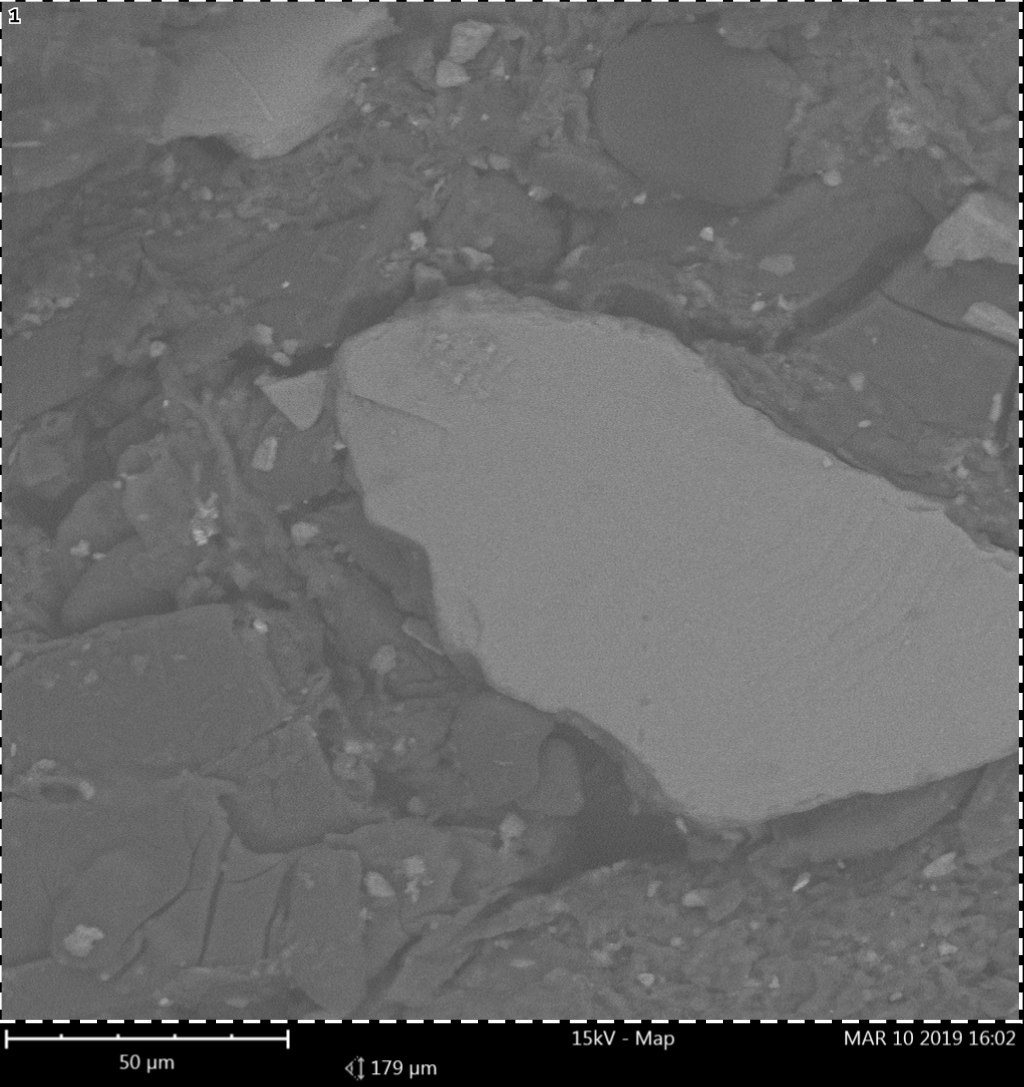


Alkali pretreated Hot water pretreated

Figure S19 : SEM of pretreated cassava peels plus yam peels biomass (300 microns)

Figure S20 : SEM of pretreated all biomasses mixed (300 microns)


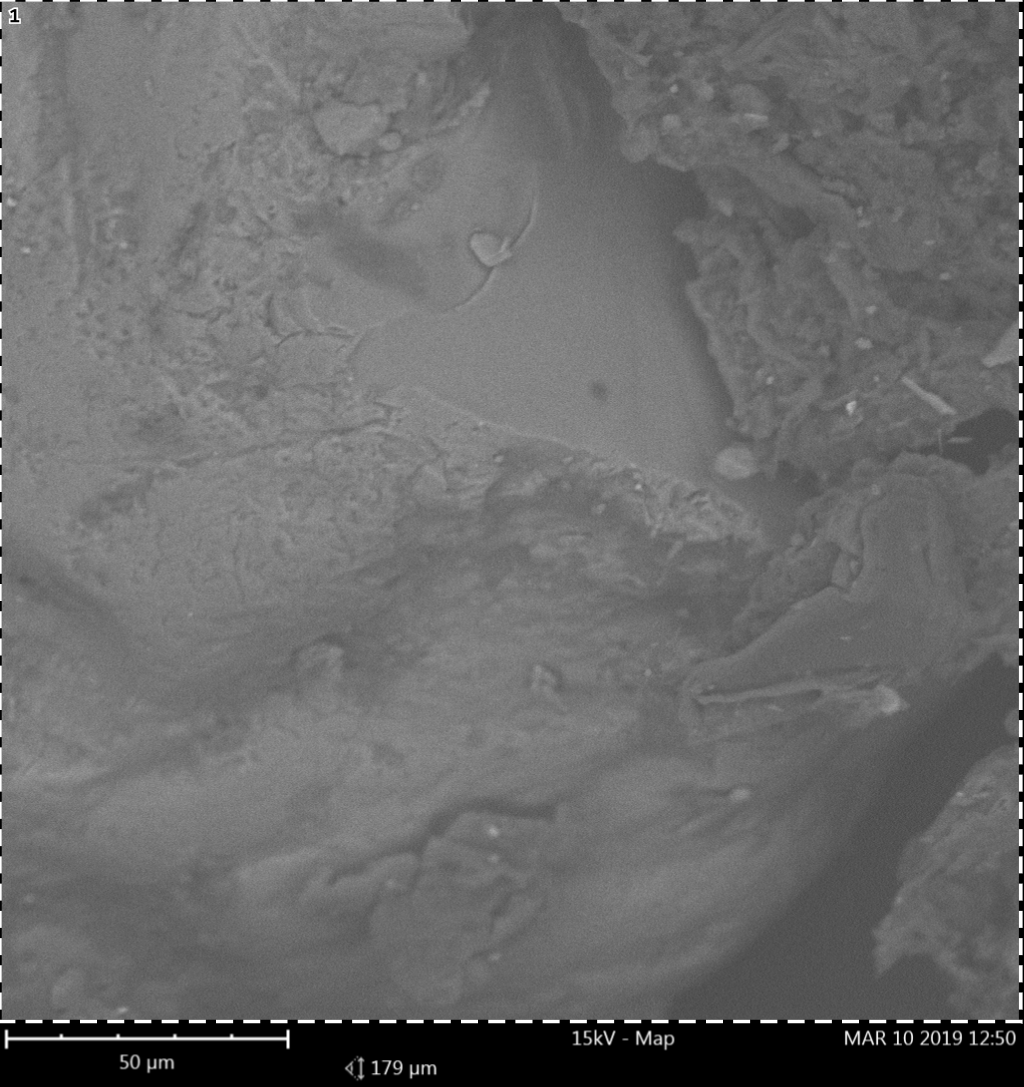

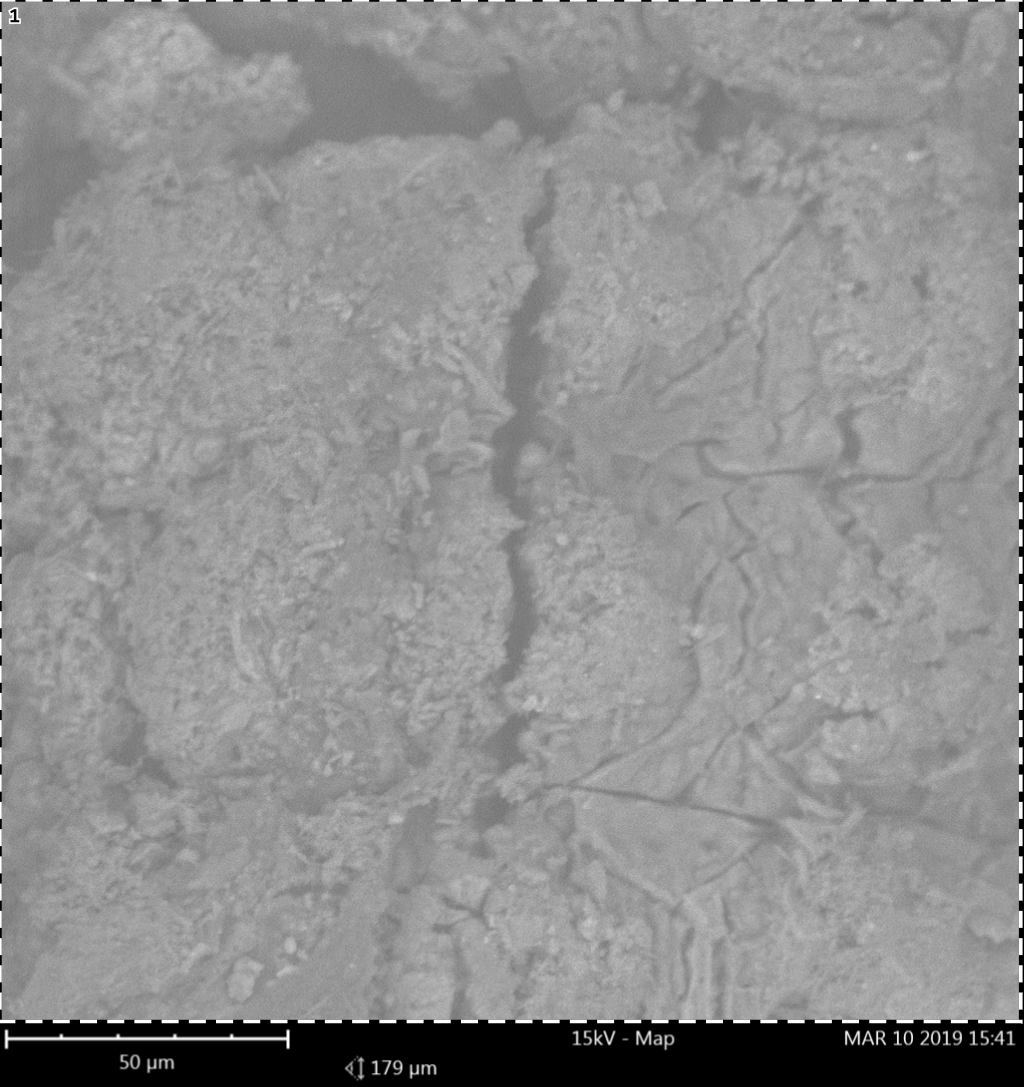


Acid pretreated Alkali pretreated


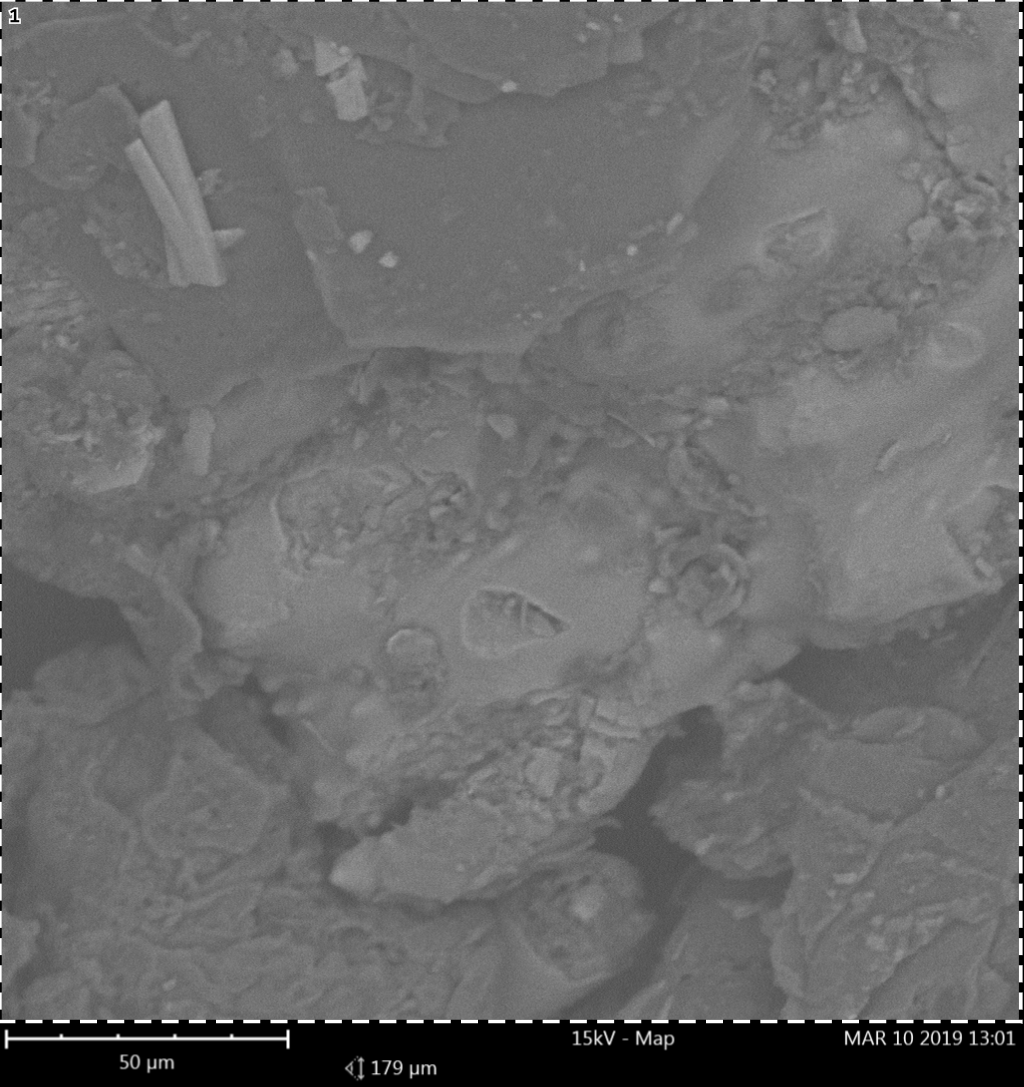


Hot water pretreated

Figure S21 : XRD of cassava peels biomass


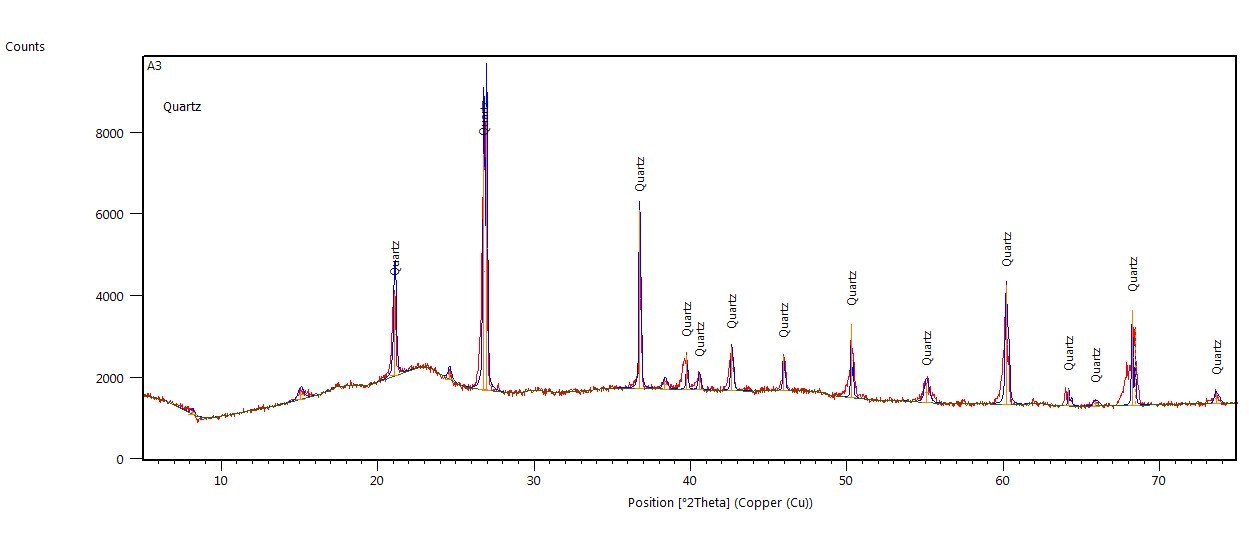

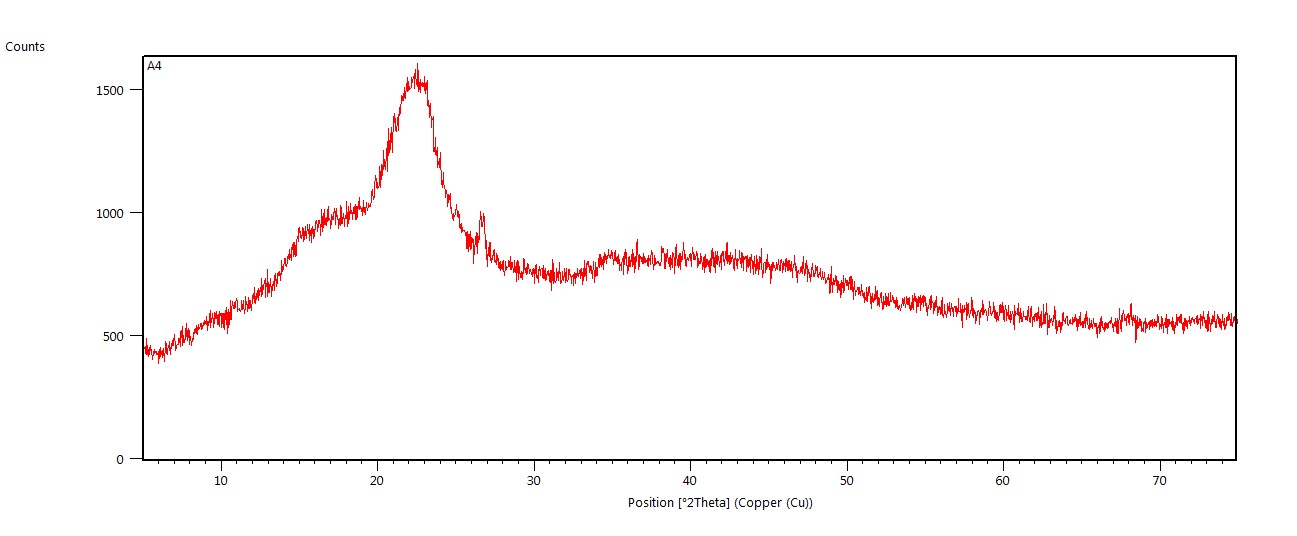


Raw cassava peels biomass 300 micron Raw cassava peels biomass 425 micron


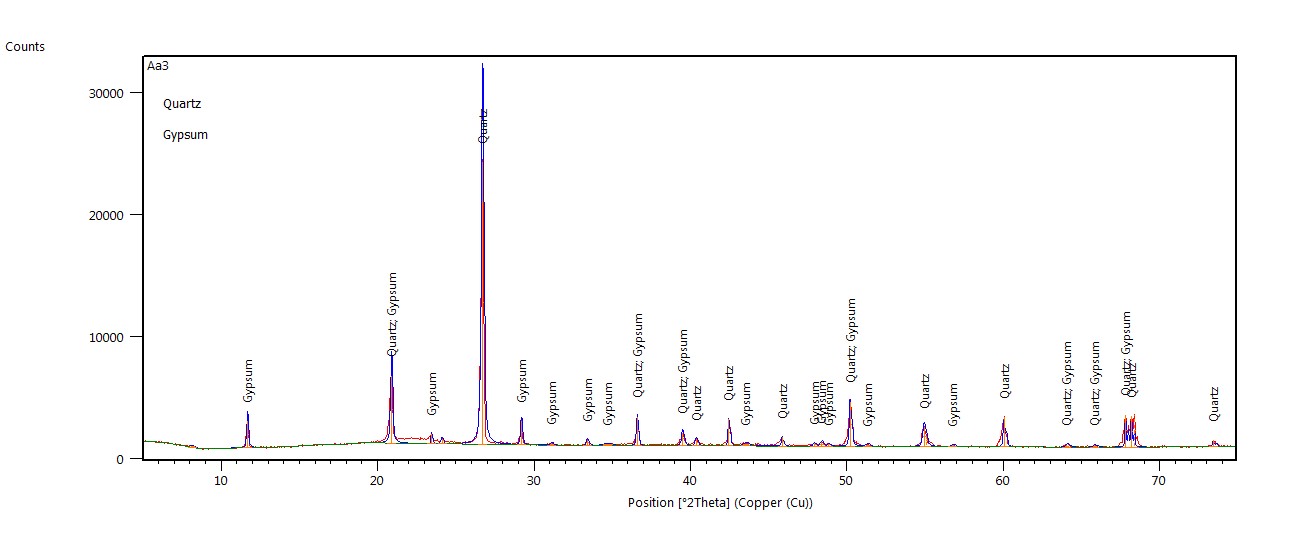


Acid pretreated 300 micron cassava peels biomass Acid pretreated 425 micron cassava peels biomass

Alkali pretreated 300 micron cassava peels biomass Hot water pretreated 300 micron cassava peels biomass

Figure S22 : XRD patterns of raw and pretreated corn cobs biomass

Raw corn cobs 300 microns Raw corn cobs 425 microns

Acid pretreated corn cobs 300 microns Acid pretreated corn cobs 425 microns

Alkali pretreated 300 microns Hot water pretreated 300 microns

Figure S23 : XRD of raw and pretreated rice husks

Raw rice husks biomass 300 microns Raw rice husks biomass 425 microns

Acid pretreated rice husks biomass 300 microns Acid pretreated rice husks biomass 425 microns

Alkali pretreated rice husks biomass 300 microns Hot water pretreated rice husks biomass 300 microns

Figure S24 : XRD of raw and pretreated sugar cane bagasse

Raw sugar cane biomass 300 microns

Alkali pretreated sugar cane biomass 300 microns Acid pretreated sugar cane biomass 300 microns

Hot water pretreated 300 microns

Figure S25 : XRD of raw and pretreated Yam peels biomass

Raw yam peels biomass 300 microns Raw yam peels biomass 425 microns

Acid pretreated yam peels biomass 300 microns Hot water pretreated yam peels biomass 300 microns

Figure S26 : XRD diffractograms of All biomasses

All biomasses mixed acid pretreated All biomasses mixed alkali pretreated

All biomasses mixed hot water pretreated
